# Dual Acid Responses of Stable Luminescent Diphenylpyridylmethyl Radicals With Additional Pyridyl Groups

**DOI:** 10.1002/chem.71095

**Published:** 2026-05-06

**Authors:** Yohei Hattori, Kojiro Nakagawa, Kingo Uchida, Gwénaël Rapenne

**Affiliations:** ^1^ Division of Materials Science Nara Institute of Science and Technology Ikoma Japan; ^2^ Materials Chemistry Course Faculty of Advanced Science and Technology Ryukoku University Seta Otsu Shiga Japan; ^3^ Université de Toulouse CEMES‐CNRS Toulouse France

**Keywords:** acid–base reactions, donor–acceptor systems, luminescence, radicals, triarylmethanes

## Abstract

Polychlorinated diphenylpyridylmethyl radicals, exemplified by (3,5‐dichloro‐4‐pyridyl)bis(2,4,6‐trichlorophenyl)methyl radical (PyBTM), have garnered significant attention as stable radical emitters. Due to their halogenated pyridyl groups, these radicals also exhibit weak basicity and acid‐responsive properties. To further explore and expand the acid‐responsive behavior of PyBTM, we substituted additional pyridyl groups (3‐pyridyl, 4‐pyridyl, and 2‐methoxy‐3‐pyridyl) for chlorine atoms at the *para*‐positions relative to the methyl radical center. This modification yielded six novel, stable luminescent radicals. Spectroscopic analysis revealed distinct changes in absorption and emission profiles upon titration with a Brønsted acid (*p*‐toluenesulfonic acid), a Lewis acid (tris(pentafluorophenyl)borane), and metal cations (Ag^+^, Zn^2+^, Cu^2+^). Complementary ^1^H NMR and DFT studies suggested that the 3‐pyridyl and 4‐pyridyl substituents act as stronger bases compared to PyBTM, whereas the basicity of the 2‐methoxy‐3‐pyridyl is attenuated. The observed optical changes, including the emergence of new absorption bands and emission quenching, were rationalized using TD‐DFT calculations.

## Graphical abstract

To expand the acid‐responsive behavior of diphenylpyridylmethyl stable luminescent radicals, additional pyridyl groups were introduced. Dual acid responsiveness was observed, involving both the newly incorporated pyridyl groups and the PyBTM moieties. In the 3‐ and 4‐pyridyl derivatives, the added pyridyl groups preferentially reacted with acids, whereas the 2‐methoxy‐3‐pyridyl substituent exhibited significantly weaker basicity.

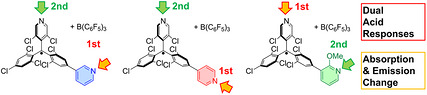

## 1 Introduction

Luminescent radicals represent a unique class of emissive materials characterized by a doublet spin‐multiplicity state [1, 2, 3]. Their fluorescence originates from the lowest doublet excited state via transition to the doublet ground state. Unlike closed‐shell molecules, where singlet‐triplet intersystem crossing often reduces emissive efficiency, luminescent radicals avoid this pathway, making them highly promising candidates for organic light‐emitting diode (OLED) emitters [4, 5, 6]. Beyond their applications in optoelectronics, these paramagnetic emissive species have also been explored for fundamental spin physics studies [7, 8], magnetoluminescence [9, 10, 11], and spin‐optical interfaces [12, 13, 14]. In addition, extensions to diradicals and multiradical systems have recently attracted attention as one approach to further diversify the properties of luminescent open‐shell materials [15, 16, 17].

The ability to modulate the photophysical properties of luminescent radicals through external stimuli offers a compelling avenue to unlock their full potential. For example, the use of open‐shell electronic structures in fluorescent probes offers advantages in the use in heavy‐atom environments [18, 19], near‐infrared emission [20, 21], and compatibility with MR imaging [22] compared to conventional closed‐shell systems.

**FIGURE 1:**
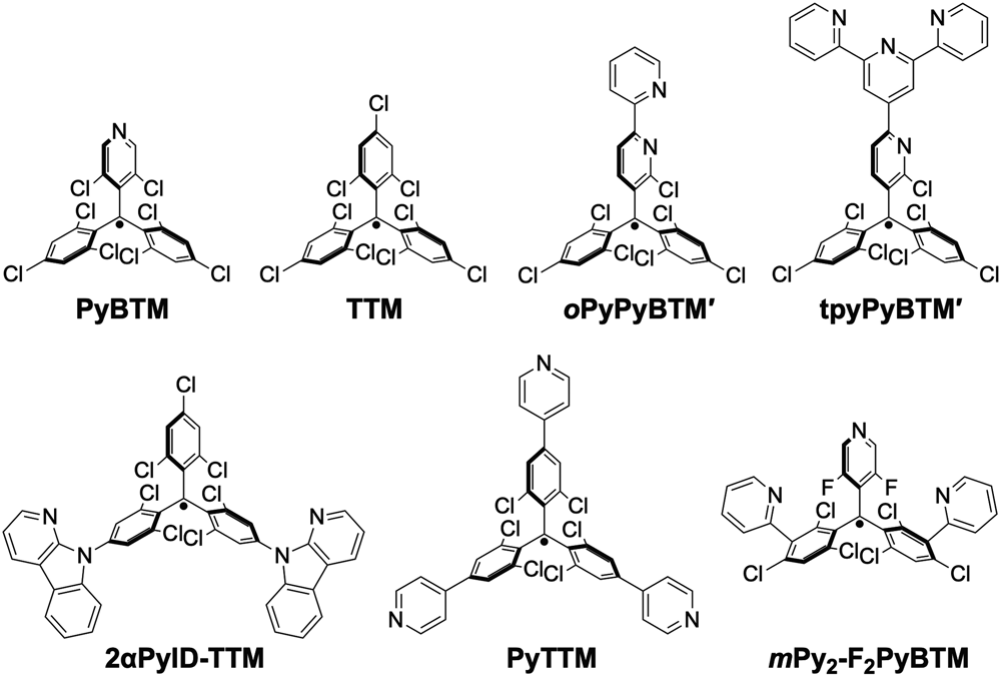
Structures of PyBTM, TTM, and previously reported pyridine‐added triarylmethyl radicals.

Among these radicals, the (3,5‐dichloro‐4‐pyridyl)bis(2,4,6‐trichlorophenyl)methyl radical (PyBTM, Figure 1) [23] stands out as a pyridine‐containing triarylmethyl radical, exhibiting exceptional stability, 70‐fold greater than tris(2,4,6‐trichlorophenyl)methyl radical (TTM) [24] under 370 nm UV irradiation in dichloromethane. PyBTM also demonstrates responsiveness to protons and Lewis acids.

However, the basicity of its 3,5‐dichloropyridyl moiety is significantly attenuated by the electronegative chlorine substituents, necessitating over 100 equivalents of *p*‐toluenesulfonic acid (TsOH) for complete protonations [23]. To enhance binding affinity, [2‐chloro‐6‐(2‐pyridyl)‐3‐pyridyl]bis(2,4,6‐trichlorophenyl)methyl radical (*o*PyPyBTM′) and [2‐chloro‐6‐(4′‐2,2′:6′,2′′‐terpyridyl)‐3‐pyridyl]bis(2,4,6‐trichlorophenyl)methyl radical (tpyPyBTM′) were designed and synthesized. These molecules exhibit near‐stoichiometric (1:1) responses to acids and metal cations, respectively [18]. Notably, the nitrogen atom in the (2‐chloro‐3‐pyridyl)bis(2,4,6‐trichlorophenyl)methyl radical (PyBTM′) framework appears inactive as a base, likely due to steric constraints. Acid‐ or metal cation‐responsive behavior has also been reported for *α*‐ or *δ*‐carboline‐functionalized TTMs (2αPyID‐TTM and 2δPyID‐TTM) [25] and pyridine‐modified TTM (PyTTM) [26].

**FIGURE 2:**
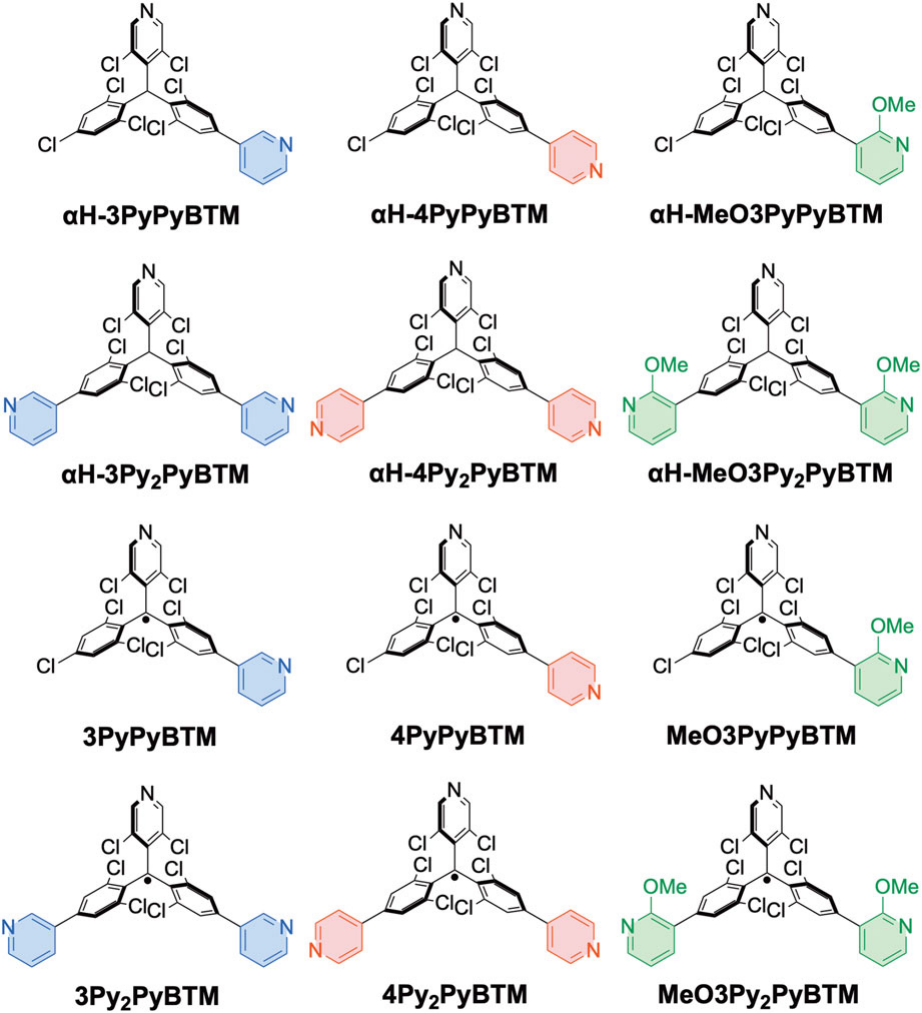
The series of novel PyBTM radicals with pyridyl donors and their precursors.

In this study, we introduced pyridyl derivatives at the *para*‐positions of PyBTM (Figure 2). Previously, we demonstrated that pyridyl groups substituted at the *meta*‐positions significantly quench the luminescence of the *m*Py_2_‐F_2_PyBTM radical [27]. Here, we investigate the competitive acid responsiveness between the newly introduced pyridyl groups and the 3,5‐dichloropyridyl moiety. The dynamic switching of the radicals’ excitation behavior is elucidated through a combination of experimental observations and DFT calculations.

## 2 Results and Discussion

### 2.1 Preparation of Pyridyl‐Substituted PyBTM Radicals

Pyridyl‐adducts were prepared from (3,5‐dichloro‐4‐pyridyl)bis(2,4,6‐trichlorophenyl)methane (αH‐PyBTM) and pyridylboronic acids using a micellar Suzuki–Miyaura coupling reaction previously employed for the preparation of phenyl‐adduct PyPBTM [28] (see Supporting Information for experimental details). Mono‐ and di‐adducts bearing 3‐pyridyl, 4‐pyridyl, and 2‐methoxy‐3‐pyridyl groups were synthesized and separated by column chromatography. The precursors, αH‐3PyPyBTM, αH‐3Py_2_PyBTM, αH‐4PyPyBTM, αH‐4Py_2_PyBTM, αH‐MeO3PyPyBTM, and αH‐MeO3Py_2_PyBTM were obtained in yields of 33%, 4.3%, 15%, 1.1%, 31%, and 20%, respectively, and were characterized by ^1^H NMR, ^13^C NMR, and HR‐MS. The corresponding radicals were then generated via a deprotonation and oxidation process, and six new radicals, 3PyPyBTM, 3Py_2_PyBTM, 4PyPyBTM, 4Py_2_PyBTM, MeO3PyPyBTM, and MeO3Py_2_PyBTM were obtained in yields of 89%, 48%, 77%, 42%, 81%, and 99%, respectively. These radicals were characterized by elemental analysis, and all of them showed ESR (EPR) signals at *g* = 2.003 (Figure S1). The presence of an *S* = 1/2 spin on each molecule was confirmed for all the radicals by comparing the double‐integrated signal intensity with that of a reference sample (TEMPO) in toluene.

### 2.2 Absorption and Emission Properties

**FIGURE 3:**
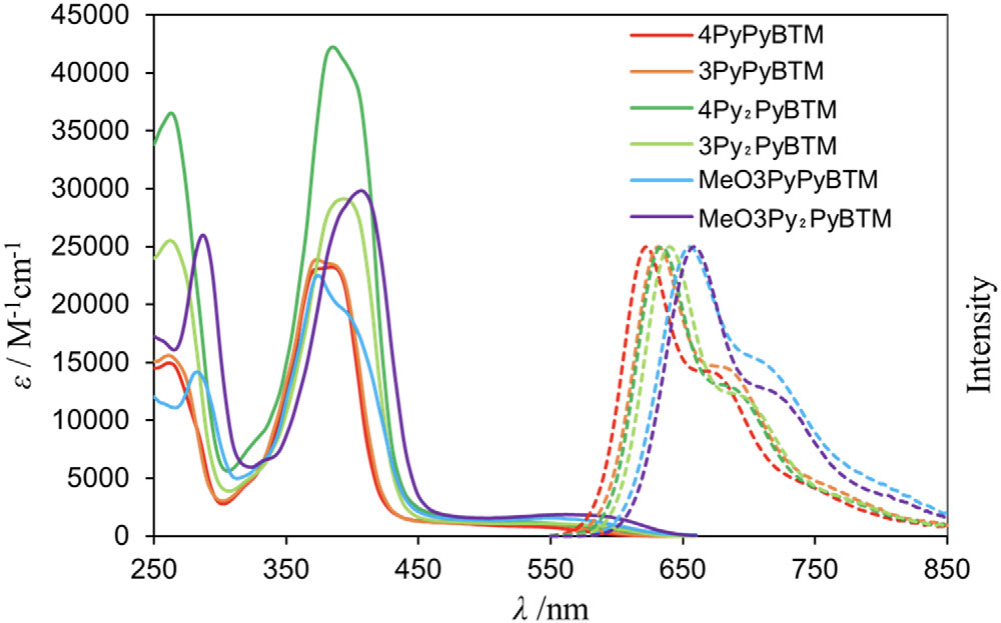
Absorption and emission spectra of 3PyPyBTM, 3Py_2_PyBTM, 4PyPyBTM, 4Py_2_PyBTM, MeO3PyPyBTM, and MeO3Py_2_PyBTM in chloroform.

Absorption and emission spectra of the radicals in chloroform are shown in Figure 3. The spectra feature strong absorption bands between 350 and 450 nm and weaker bands between 450 and 650 nm, consistent with the typical profile of triarylmethyl radicals. In general, the di‐adducts exhibited more intense absorption than their corresponding mono‐adducts. The emission maxima were observed at 622, 631, 634, 640, 655, and 658 nm for 4PyPyBTM, 3PyPyBTM, 4Py_2_PyBTM, 3Py_2_PyBTM, MeO3PyPyBTM, and MeO3Py_2_PyBTM, respectively. Relative to the emission maximum of PyPBTM (= Ph_2_PyBTM) at 650 nm [28], 4Py_2_PyBTM and 3Py_2_PyBTM displayed blue‐shifted emission, while MeO3Py_2_PyBTM showed red‐shifted emission. The observed emission wavelengths order reflects the electron‐donating strength of the donor groups (4‐pyridyl < 3‐pyridyl < phenyl < 2‐methoxy‐3‐pyridyl). Additionally, the introduction of two donor groups resulted in a slight bathochromic shift in the emission wavelength.

The absolute photoluminescence quantum yields (PLQYs) and fluorescence lifetimes are summarized in Table 1. In chloroform, the PLQYs of these radicals were modestly higher than that of PyBTM (*Φ*_f_ = 3%) [23], but lower than that of PyPBTM (*Φ*_f_ = 8.6%). Notably, emission efficiency improved slightly in polar solvents such as dichloromethane and acetone (Tables S1–S4), a trend consistent with systems in which the electron‐donating capacity of the substituent is limited [29]. Radicals bearing methoxypyridyl groups exhibited marginally higher PLQYs, primarily due to increased radiative rate constants (*k*_f_). However, this enhancement was partially offset by a concurrent rise in non‐radiative decay rate constants (*k*_nr_). The larger *k*_f_ likely arises from the superior donor ability of the methoxypyridyl group, whereas the larger *k*_nr_ can be explained by the smaller excitation energies and the energy‐gap law. Opposite effects, namely smaller *k*_f_ and *k*_nr_, were observed for the 4‐pyridyl adducts.

**TABLE 1:** Photophysical parameters of the emission of the radicals in chloroform.

	*Φ*_f_ (%)	*τ*/ns	*k*_f_/10^7^ s^−1^	*k*_nr_/10^7^ s^−1^
3PyPyBTM	3.6	9.1	0.40	10.0
3Py_2_PyBTM	4.9	10.2	0.48	9.0
4PyPyBTM	3.5	14.6	0.24	6.6
4Py_2_PyBTM	4.9	16.0	0.31	5.9
MeO3PyPyBTM	4.6	6.2	0.70	15.4
MeO3Py_2_PyBTM	5.3	6.8	0.78	13.9

**FIGURE 4:**
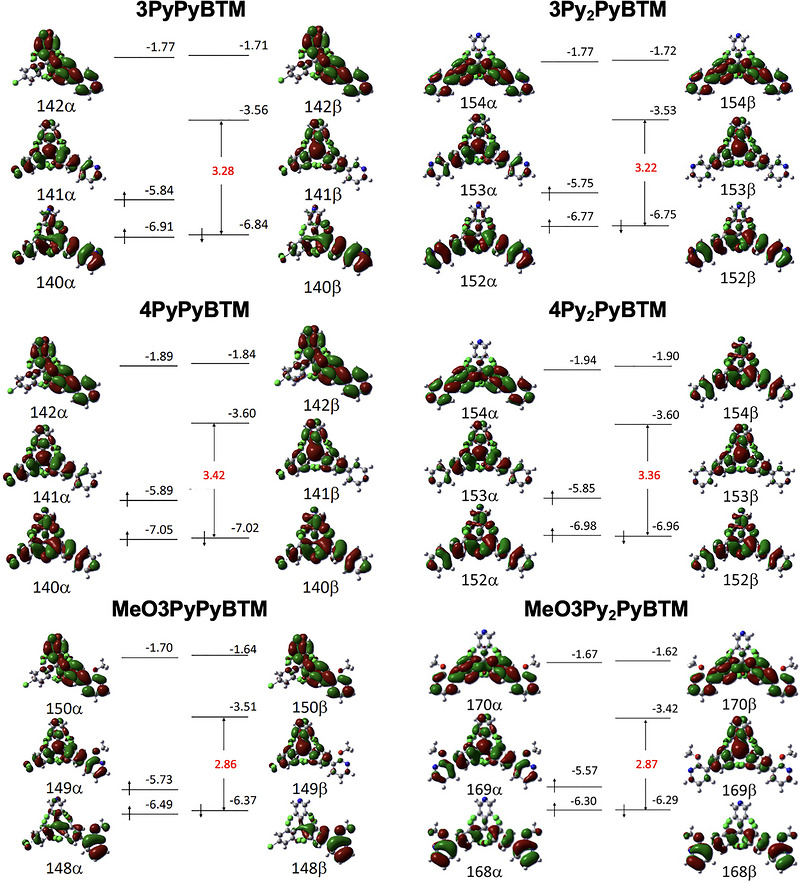
DFT‐calculated frontier orbitals of the radicals in chloroform at the UB3LYP/6‐31G(d,p) level. Orbital energies are shown in eV.

The geometries of the radicals were optimized using DFT calculations at the UB3LYP/6‐31G(d,p) level, with the solvent effect of chloroform accounted for via a polarizable continuum model (PCM) [30, 31]. Subsequent TD‐DFT calculations were performed to analyze the excitation properties of these molecules. The calculated frontier orbitals of the radicals are presented in Figure 4. Given that the smallest energy gaps between occupied and unoccupied spin orbitals correspond to the β‐HOMO–LUMO transitions, these radicals can be classified as donor–radical–acceptor systems. In this framework, the β‐HOMOs mainly distributed on the added pyridyl groups interact with the β‐LUMOs localized on the PyBTM moiety. This electronic configuration rationalizes the higher radiative rate constants (k_f_) observed for these radicals compared to PyBTM [32].

While the energy levels of the radical‐centered β‐LUMO orbitals remain consistent at −3.5 ± 0.1 eV, the radicals featuring methoxypyridyl groups exhibit β‐HOMO orbitals elevated by over 0.38 eV compared to the other radicals. Consequently, the TD‐DFT‐calculated first excitation energies for these methoxypyridyl‐substituted radicals are lower (see Supporting Information), resulting in red‐shifted absorption and emission profiles. The oscillator strengths follow the order: (MeO3Py_2_PyBTM > MeO3PyPyBTM > 3Py_2_PyBTM > 3PyPyBTM > 4Py_2_PyBTM > 4PyPyBTM), which closely mirrors the trend in absorbance at 550 nm. Additionally, strong absorptions arising from α‐HOMO–LUMO transitions are predicted at 400 nm, 405 nm, 399 nm, 408 nm, 402 nm, and 427 nm for 3PyPyBTM, 3Py_2_PyBTM, 4PyPyBTM, 4Py_2_PyBTM, MeO3PyPyBTM, and MeO3Py_2_PyBTM, respectively. These shifts correspond to the evolution of intense absorption bands upon incorporation of the second pyridyl group.

### 2.3 ^1^H NMR Titrations

To investigate the acid reactivity of each functional group in the radicals, we performed titrations of the precursors αH‐3PyPyBTM, αH‐4PyPyBTM, and  αH‐MeO3PyPyBTM with TsOH in chloroform‐d_1_ (For the detailed procedure, see Section I.4 of the SI). For reference, titration of αH‐PyBTM with TsOH revealed gradual downfield shifts of the protons on the 3,5‐dichloropyridyl and the αH proton (Figure S2). After the addition of 1.5 eq., these shifts slowed but persisted due to the weak basicity of the 3,5‐dichloropyridine moiety. The protonated species αH‐PyBTMH^+^ likely exhibits downfield shifts as a result of deshielding, with averaged shifts observed during intermediate stages of the titration.

**FIGURE 5:**
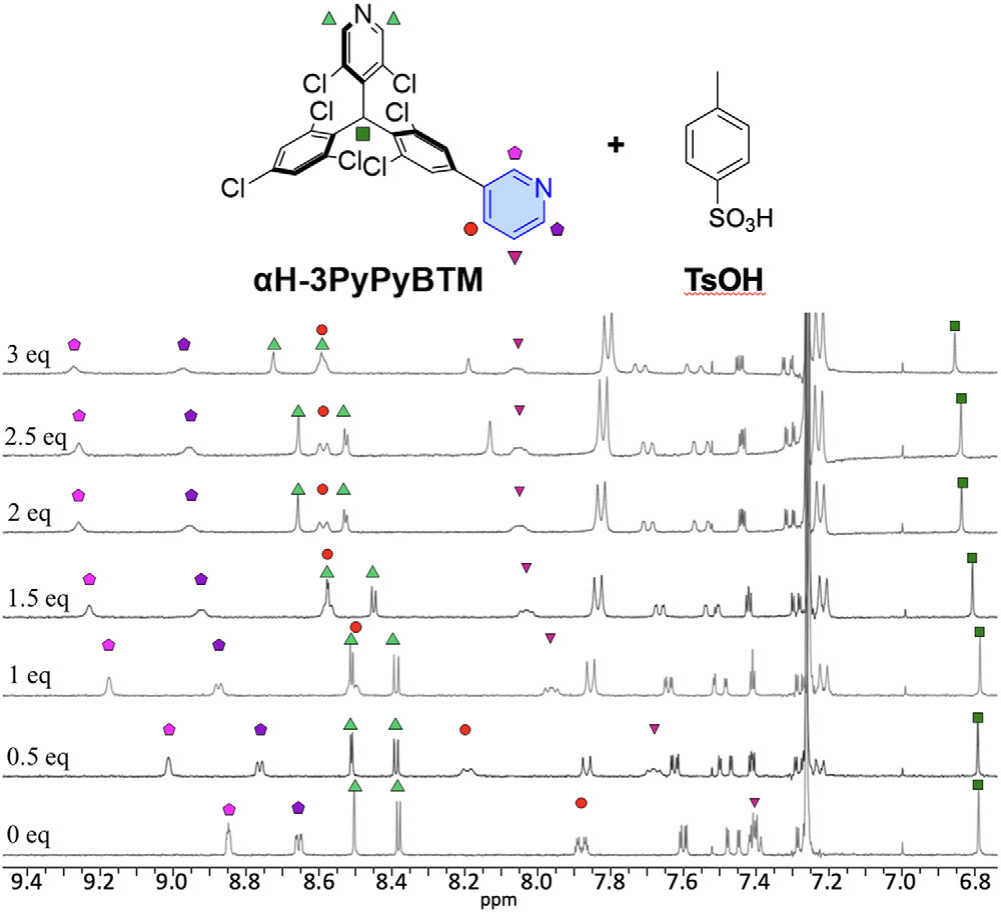
^1^H NMR spectral changes of αH‐3PyPyBTM during the addition of TsOH.

In the case of αH‐3PyPyBTM, the ^1^H NMR signals also shifted progressively downfield (Figure 5). Upon addition of up to 1.5 eq. of TsOH, the protons on the 3‐pyridyl donor experienced significant shifts. After 1 eq., downfield shifts of the protons on the 3,5‐dichloropyridyl group and the αH proton began, although these shifts were less pronounced than those observed for αH‐PyBTM, likely due to the increased difficulty of the second protonation. A similar trend was observed for αH‐4PyPyBTM (Figure S3).

In contrast, αH‐MeO3PyPyBTM exhibited simultaneous downfield shifts of the protons on the 2‐methoxy‐3‐pyridyl, the 3,5‐dichloropyridyl groups, and the αH proton (Figures S4 and S5). While the presence of an electron‐donating methoxy group might intuitively suggest enhanced basicity, its adjacent position likely introduces steric hindrance, thereby hindering protonation at the pyridyl site.

To compare the reactivity of Brønsted and Lewis acids, we employed tris(pentafluorophenyl)borane (B(C_6_F_5_)_3_) [33]. In the case of αH‐3PyPyBTM, the ^1^H NMR signals of the unprotonated compound remained unchanged upon addition of B(C_6_F_5_)_3_, but new signals corresponding to the adduct emerged (Figure S6). This behavior suggests that the dissociation rate of the αH‐3PyPyBTM−B(C_6_F_5_)_3_ complex is slow on the ^1^H NMR timescale. Notably, some signals shifted upfield, reflecting the neutral character of B(C_6_F_5_)_3_, in contrast to the downfield shifts observed upon protonation. As in the Brønsted acid titration, the reaction initially targeted the 3‐pyridyl donor, with subsequent involvement of the 3,5‐dichloropyridyl group after the addition of 1 eq. The reaction profile of αH‐4PyPyBTM closely mirrored that of αH‐3PyPyBTM (Figure S7).

**FIGURE 6:**
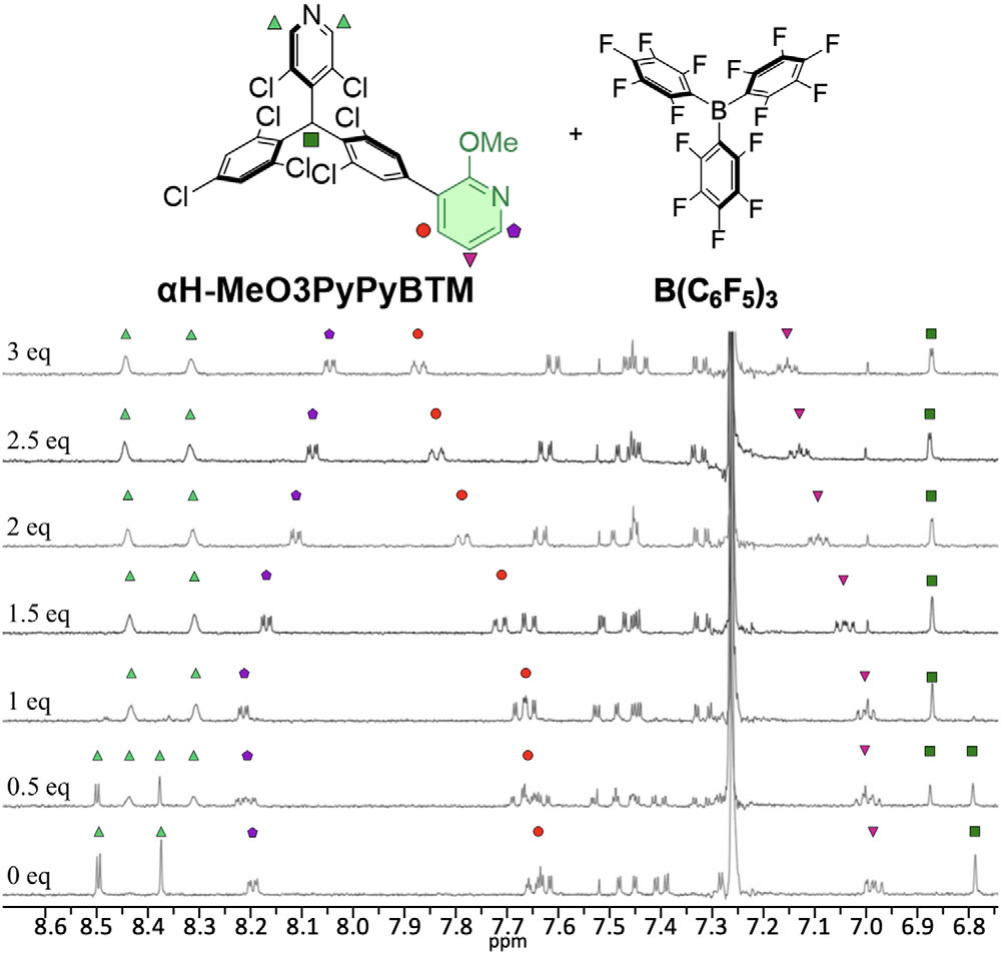
^1^H NMR spectral changes of αH‐MeO3PyPyBTM during the addition of B(C_6_F_5_)_3_.

In the case of αH‐MeO3PyPyBTM, the 3,5‐dichloropyridyl group reacted preferentially (Figure 6). Following the addition of 1 eq. of B(C_6_F_5_)_3_, the signals of the 2‐methoxy‐3‐pyridyl protons exhibited both downfield and upfield shifts. These shifts likely reflect a rapid dissociation equilibrium between the 2‐methoxy‐3‐pyridyl group and B(C_6_F_5_)_3_, driven by steric repulsion.

Ideally, we would perform similar experiments with the radicals; however, they are not suitable for NMR measurements. Therefore, we use the results of the precursors as an indirect estimate of the behavior of the radicals, assuming that a neutral hydrogen atom does not affect the basicity of the pyridines.

### 2.4 UV‐Vis and Fluorescence Titration

**FIGURE 7:**
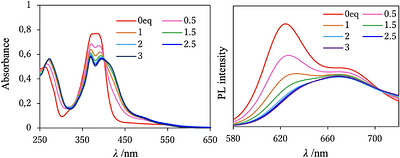
Absorption and emission changes of 4PyPyBTM (*λ*_ex_ = 372 nm) during the addition of TsOH in chloroform.

The addition of TsOH to the radical in chloroform induced distinct changes in both the absorption and emission spectra. In the case of 4PyPyBTM, the addition of up to 1.5 eq. of TsOH resulted in a significant decrease in absorption at ∼375 nm, a pronounced increase at ∼425 nm, and a marked reduction in emission at ∼625 nm (Figure 7). Based on the ^1^H NMR titration results of αH‐4PyPyBTM, these spectral shifts correspond to protonation of the 4‐pyridyl donor. Notably, these changes were reversible upon the addition of triethylamine (Figure S8). After the addition of 1.5 eq. of TsOH, minor spectral changes persisted, likely due to the protonation of the remaining 4‐pyridyl donor and the 3,5‐dichloropyridyl group. While it is established that over 100 eq. of TsOH are required for complete protonation of the 3,5‐dichloropyridyl moiety [23], the pyridyl‐substituted radicals underwent photodegradation upon the addition of excess TsOH. Consequently, the reaction was no longer reversible after neutralization with Et_3_N. Accordingly, further spectral changes upon additional TsOH addition are not discussed.

In the case of 3PyPyBTM, similar changes were observed, although the profiles differed slightly (Figure S9). The absorption changes were less pronounced, and the emission quenching occurred more uniformly across the wavelength range. In the case of MeO3PyPyBTM, the addition of TsOH induced only minor spectral changes (Figure S10), consistent with the weak basicity of the 2‐methoxy‐3‐pyridyl group, as confirmed by the ^1^H NMR titration of αH‐MeO3PyPyBTM. When TsOH was added to the di‐adducts, similar changes to those of the mono‐adducts were observed (Figure S11). In the case of 3Py_2_PyBTM and 4Py_2_PyBTM, the spectral shifts can be attributed to the initial protonation of the two pyridyl donors, followed by protonation at the 3,5‐dichloropyridyl group. Similar to MeO3PyPyBTM, the spectral changes in MeO3Py_2_PyBTM were minimal.

**FIGURE 8:**
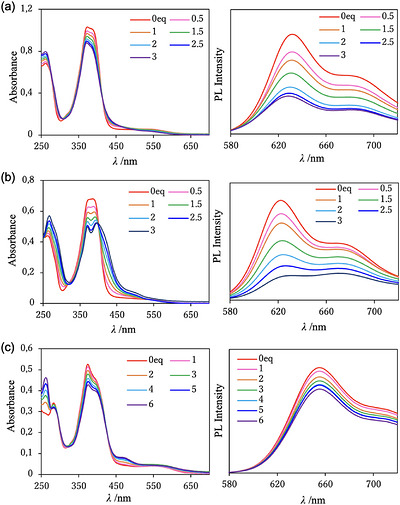
Absorption and emission changes of (a) 3PyPyBTM (*λ*_ex_ = 373 nm), (b) 4PyPyBTM (*λ*_ex_ = 372 nm), and (c) MeO3PyPyBTM (*λ*_ex_ = 374 nm) during the addition of B(C_6_F_5_)_3_ in chloroform.

The spectral changes induced by the Lewis acid were more pronounced than those caused by the Brønsted acid (Figures 8 and S12). For 3PyPyBTM and 4PyPyBTM, substantial spectral shifts persisted even after the addition of 1.5 eq., gradually altering the spectral profiles up to at least 3 eq. In the case of MeO3PyPyBTM, distinct spectral changes were evident up to at least 6 eq. and were reversible upon the addition of 10 eq. of Et_3_N (Figure S13). These observations can be attributed to the strong affinity of B(C_6_F_5_)_3_ for the 3,5‐dichloropyridyl group.

Stimuli responsiveness was also observed with metal cations. Previous studies have reported fluorescence enhancement when monovalent group 11 transition metals (Au^+^ [34], Ag^+^) [35] are directly coordinated with PyBTM. When silver(I) trifluoromethanesulfonate (AgOTf) was added to 4PyPyBTM in chloroform, the absorption at ∼375 nm decreased, absorption at ∼450 nm increased, and emission decreased (Figure S14). The changes were slightly smaller for 3PyPyBTM and minimal for MeO3PyPyBTM. Overall, the spectral modifications induced by AgOTf closely resembled those caused by TsOH. In the case of 3Py_2_PyBTM and 4Py_2_PyBTM, the addition of 3 eq. of AgOTf resulted in slight redshifts of the absorption maxima and more significant emission quenching (Figure S15). This is likely due to the coordination of Ag^+^ to both pyridyl donors. The changes in MeO3Py_2_PyBTM were minor except in the UV region, consistent with the behavior of MeO3PyPyBTM.

**FIGURE 9:**
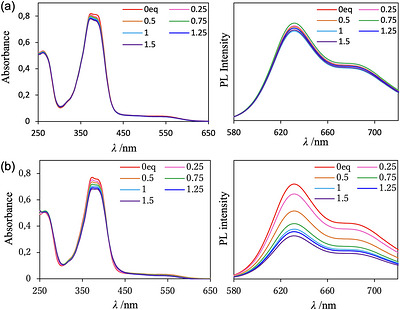
Absorption and emission changes of 3PyPyBTM (*λ*_ex_ = 373 nm) by addition of (a) Zn(OTf)_2_ and (b) Cu^II^(OTf)_2_ in chloroform.

The addition of the divalent metal salt zinc(II) trifluoromethanesulfonate (Zn(OTf)_2_) to the radicals in chloroform induced only minor spectral changes. In contrast, the introduction of copper(II) trifluoromethanesulfonate (Cu(OTf)_2_) resulted in spectral alterations similar to those observed with AgOTf (Figures 9, S16 and S17). Notably, a smaller stoichiometric amount of the copper salt was sufficient to achieve near‐complete spectral changes, likely due to the dicationic nature of Cu^2^^+^. The divalent copper cation exhibits a stronger coordination field, enhanced by the Jahn–Teller effect, and forms complexes with maximal stability according to the Irving–Williams series. Consequently, the difference between Zn^2^^+^ and Cu^2^^+^ proved critical for their interactions with the pyridyl donors in these radicals. For radicals featuring methoxypyridyl donors, the observed spectral changes were similarly modest. This behavior aligns with the properties of PyBTM complexes with divalent metal ions, which tend to dissociate in organic solvents, precluding detailed solution‐phase characterization [36]. While these reactions were largely reversible upon the addition of excess Et_3_N, the process appeared to involve complex side reactions, including the formation of (Et_3_N)Cu(Pyridyl radicals) species.

### 2.5 DFT Calculations

To model the acid‐radical adducts, we optimized the structures of TsOH‐ and BH_3_‐ bound 3PyPyBTM, 4PyPyBTM, and  MeO3PyPyBTM using DFT calculations at the UB3LYP/6‐31G(d,p) level in chloroform. Preliminary calculations of cationic species such as 3PyPyBTMH^+^ yielded excessively red‐shifted absorption spectra, suggesting that these models poorly represent the actual solution‐phase species. Although the BH_3_ model does not capture the steric effects of B(C_6_F_5_)_3_ and only partially reflects its electronic effect, it still enables a qualitative discussion of pyridine coordination to boron.

Acids can coordinate either to the PyBTM (3,5‐dichloropyridyl) nitrogen or to the nitrogen atoms of the added pyridyl groups. We compared the optimized energies of both coordination isomers and calculated the acid–base reaction energies (Tables S5 and S6). For 3PyPyBTM and 4PyPyBTM with TsOH, the Gibbs free energy changes (Δ*G*) were calculated to be −6.6 and −8.7 kJ mol^−1^, respectively, when protonation occurred at the added pyridyl groups, and +5.0 and +3.1 kJ mol^−1^ when protonation involved the PyBTM nitrogens. While N─H bond formation contributes favorably to the enthalpy, most of this stabilization is offset by entropic effects at room temperature. Nevertheless, the calculations successfully reproduced the experimental preference for protonation at the simpler pyridyl groups over the 3,5‐dichloropyridyl moiety.

In contrast, the Gibbs free energy change for the reaction of MeO3PyPyBTM with TsOH at the added methoxypyridyl group was calculated to be Δ*G* = +15.8 kJ mol^−1^, which is larger than that at the PyBTM (Δ*G* = +3.4 kJ mol^−1^). Although this value may be slightly overestimated based on the ^1^H NMR results, the calculations successfully capture the relatively weak basicity of the 2‐methoxy‐3‐pyridyl group and the corresponding minimal spectral changes observed experimentally.

The differences among the pyridyl groups were more pronounced in the BH_3_ models. The calculated Gibbs free energy changes for the reactions were approximately −90 kJ mol^−1^ for the simple pyridyl groups, around −80 kJ mol^−1^ for the PyBTM 3,5‐dichloropyridyl groups, and −50 kJ mol^−1^ for the 2‐methoxypyridyl group. Although these values are likely more negative than those for the experimentally used B(C_6_F_5_)_3_, they effectively explain the preferred reaction sites.

The frontier orbitals of the TsOH‐bound compounds are shown in Figure S18. In some cases, the β‐HOMO orbitals were localized on TsOH, likely due to the dissociation equilibrium between TsO^−^ and a proton, which raises the energy of these orbitals. Meanwhile, the β‐LUMO orbitals centered on PyBTM were stabilized by 0.1−0.2 eV.

The TD‐DFT calculations rationalize the spectral changes observed for 4PyPyBTM (see Supporting Information). Initially, TsOH protonates the added pyridyl group. In this adduct, the excitation with the largest oscillator strength, which is primarily derived from the α‐HOMO–LUMO transition, is predicted at 429 nm. The oscillator strengths of longer wavelength absorptions (461 and 454 nm) also increase due to mixing with the α‐HOMO–LUMO transition. This effect arises from the lowering of the α‐LUMO energy upon pyridine protonation, which reduces the gap between the α‐HOMO–LUMO and β‐HOMO–LUMO transitions. The α‐HOMO–LUMO+1 transition around 370 nm, which is largely independent of the added pyridyl group, does not shift upon protonation; however, its oscillator strength is slightly reduced. These excited states account for the absorption spectral changes observed up to the addition of 1.0 eq. addition of TsOH (Figure 7). In contrast, when TsOH protonates the PyBTM moiety, a strong α‐HOMO–LUMO‐derived absorption is expected at 409 nm with minimal spectral shifting.

In the case of 3PyPyBTM, the excitation with the largest oscillator strength, primarily derived from the α‐HOMO–LUMO transition, is predicted at 418 nm. The decrease in the α‐LUMO energy is smaller due to the absence of resonance with the nitrogen atom in the *meta*‐position. The oscillator strengths of longer wavelength absorptions are only weakly affected owing to the larger gap between the α‐HOMO–LUMO and β‐HOMO–LUMO transitions. The decrease in the oscillator strength of the α‐HOMO–LUMO+1 transition is also smaller. Thus, the spectral changes accompanying the addition of TsOH (Figure S9) can be explained.

In the case of MeO3PyPyBTM, smaller spectral changes are also predicted by TD‐DFT calculations. However, the very small spectral changes observed experimentally (Figure S10) are better explained by an equilibrium shifted toward the original compound due to its weak basicity.

Although we were unable to optimize the D_1_ excited‐state structures in this study, insights into fluorescence behavior can be derived from the Franck–Condon excited states of the first excitations (Table S7). For 3PyPyBTM with protonation at the added pyridine, the oscillator strength of the first excitation (*f* = 0.0253) is smaller than that of unprotonated 3PyPyBTM (*f* = 0.0699). This excitation primarily involves a transition from the β‐HOMO‐3 (182β, Figure S18) to β‐LUMO (186β). Since the β‐HOMO‐3 is no longer localized on the pyridyl moiety due to protonation, the disruption of the donor–radical–acceptor system explains the emission quenching observed upon TsOH addition (Figure S9).

In the case of 4PyPyBTM with TsOH bound at added pyridine, the oscillator strength of the first excitations is significantly reduced (*f* = 0.0063), as this transition corresponds to charge transfer from TsOH to PyBTM. Excluding these intermolecular transitions, the third excited state, comprising β‐HOMO‐4→β‐LUMO, α‐HOMO→α‐LUMO, and β‐HOMO‐3→β‐LUMO transitions, has a small oscillator strength (*f* = 0.0301), consistent with the observed emission quenching (Figure 7).

The frontier orbitals of the BH_3_‐bound compounds are shown in Figure S19. Despite structural differences between BH_3_ and B(C_6_F_5_)_3_, the TD‐DFT results qualitatively explain absorption spectral changes such as redshifts. The TD‐DFT‐calculated α‐HOMO–LUMO transition is predicted at 419 nm for the BH_3_‐bound 4‐PyPyBTM. This value is longer than that of the corresponding transition at 405 nm for the BH_3_‐bound 3‐PyPyBTM coordinated at the added pyridyl group, accounting for the spectral difference between these isomers (Figure 8).

In the case of MeO3PyPyBTM, the reduced gap between the α‐HOMO–LUMO and β‐HOMO–LUMO transitions in the BH_3_‐bound MeO3‐PyPyBTM coordinated at the 3,5‐dichloropyridyl group accounts for the absorption increase around 450–500 nm, although only a slight shift in wavelength is reproduced, possibly due to the simplified nature of the BH_3_ models.

**FIGURE 10:**
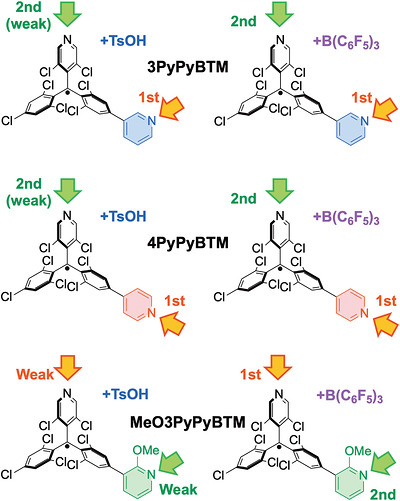
Summary of the dual acid responses of 3PyPyBTM, 4PyPyBTM, and MeO3PyPyBTM, based on NMR models, UV–vis titrations, and DFT calculations.

The dual acid reactivity with 3PyPyBTM, 4PyPyBTM, and MeO3PyPyBTM is summarized in Figure 10.

The emission quenching can be partially attributed to the reduced oscillator strengths of the first excited states of the compounds with BH_3_ coordinated at the added pyridyl groups (Table S7). Although the oscillator strengths are calculated to be larger for compounds with BH_3_ bound to the PyBTM moieties, this effect may be offset by redshifts of the first excitations, resulting in faster internal conversion according to the energy‐gap law.

## 3 Conclusion

In summary, pyridyl groups were introduced to diphenylpyridylmethyl radicals, and six new stable luminescent radicals were synthesized and characterized. Dual acid responsiveness was observed, involving both the newly incorporated pyridyl groups and the PyBTM moieties. In the 3‐pyridyl and 4‐pyridyl derivatives, the additional pyridyl groups preferentially reacted with acids, whereas the 2‐methoxy‐3‐pyridyl substituent exhibited significantly weaker basicity. Upon titration with the Lewis acid B(C_6_F_5_)_3_, the dual acid responses were clearly evident from the spectral changes. Notably, only the 2‐methoxy‐3‐pyridyl derivatives initially reacted at the PyBTM moiety. These radicals also displayed responsiveness to metal cations. The similarity in coordination behavior between Ag^+^ and H^+^, as well as the stronger coordination ability of Cu^2+^ compared to Zn^2+^, was inferred from spectroscopic changes.

This fundamental study provides new insights into switching the luminescent properties of these radicals and may be useful for sensing applications. Further quantitative studies are required to evaluate their sensing performance, and future efforts will focus on improving the photostability and responsiveness of these radicals.

## Conflicts of Interest

The authors declare no conflict of interest.

## Supporting information



Supporting Information available with ^1^H‐NMR, ^13^C‐NMR, HR‐MS, and DFT calculations data. **Supporting File**: chem71095‐sup‐0001‐SuppMat.pdf.

## Data Availability

The data that support the findings of this study are available in the Supporting Information of this article.
