# Supplementary material for: Dual Acid Responses of Stable Luminescent Diphenylpyridylmethyl Radicals With Additional Pyridyl Groups
Source: Chemistry. 2026 May 6;32(31):e71095. doi: 10.1002/chem.71095 (PMC13485194; doi:10.1002/chem.71095)

Supporting Information

Dual Acid Responses of Stable Luminescent Diphenylpyridylmethyl Radicals with Additional Pyridyl Groups

Yohei Hattori,*[a] Kojiro Nakagawa,[a] Kingo Uchida,[b] and Gwénaël Rapenne*[a,c]

[a] Division of Materials Science, Nara Institute of Science and Technology, 8916–5 Takayama, Ikoma, 630–0192, Japan

[b] Materials Chemistry Course, Faculty of Advanced Science and Technology, Ryukoku University, Seta, Otsu, Shiga 520-2194, Japan

[c] Université de Toulouse, CEMES-CNRS, 29, rue Marvig, 31055 Toulouse, France

Content

I. Experimental section S2

1. General methods and materials S2

2. Synthesis of precursors with pyridine donors using pyridylboronic acid S3

3. Synthesis of radicals with pyridine donor S5

4. ^{1}H NMR titration method S7

Figure S1. ESR spectra S8

Figures S2-S7. ^{1}H NMR spectra S9

Figures S8-S17. UV-Vis and fluorescence spectra. S12

Figures S18-S19. DFT-calculated frontier orbitals. S19

Tables S1-S4 Photophysical properties in various solvents S21

Tables S5-S7 DFT calculated energies S22

II. Cartesian coordinates of the optimized geometries by DFT calculation S24

III. Results of TD-DFT calculations S42

IV. NMR spectra S85

V. HR-MS spectra S91

I. EXPERIMENTAL SECTION

1. General methods and materials

All reagents and solvents were used without further purification, unless otherwise stated. Et_3N, Celite, anhydrous THF, anhydrous Et_2O, $CHCl_3$, ethyl acetate, hexane, acetone, toluene, and I_2 were purchased from Fujifilm Wako Pure Chemical Corporation. 4-Pyridylboronic acid, 3-pyridylboronic acid, 2-methoxy-3-pyridylboronic acid, 4-pyridylboronic acid pinacol ester, and 3-pyridylboronic acid pinacol ester were purchased from Tokyo Chemical Industry Co., Ltd. CH_2Cl_2, Et_2O, $Na_2S_2O_3 \cdot 5H_2O$, and sodium sulfate were purchased from Nacalai Tesque, Inc. ($Pd(dtbpf)Cl_2$) was purchased from Combi-Blocks, Inc. $CDCl_3$ was purchased from Eurisotop. The starting material, (3,5-dichloro-4-pyridyl)bis(2,4,6-trichlorophenyl)methane (αH-PyBTM) was prepared according to literature procedure.[23] Silica gel column chromatography and thin-layer (TLC) chromatography were performed using CHROMATOREX PSQ 60B and Merck silica gel 60 (F254) TLC plates, respectively. ^1H and ^{13}C NMR spectra were recorded on a JEOL JNM-ECX400P (400 MHz for ^1H; 101 MHz for ^{13}C) at a constant temperature of 293 K. A residual solvent peak was used as an internal reference for ^1H and ^{13}C NMR measurements in $CDCl_3$. Chemical shifts (δ) are reported in ppm. Coupling constants (J) are given in Hz and the following abbreviations have been used to describe the signals: singlet (s); broad singlet (br. s); doublet (d); triplet (t); doublet of doublet (dd); multiplet (m). The absorption spectra (UV-vis) were obtained using Hitachi U-3310 spectrometer in chloroform solutions at room temperature in 1.0 cm quartz cells (λ in nm and ε in $M^{-1}cm^{-1}$). Fluorescence spectra were monitored on Hitachi F-7100 and F-7000 fluorescence spectrophotometer. EI mass spectrometry was performed using JEOL JMS-700. MALDI-TOF mass spectrometry was performed using JEOL JMS-S300 spectrometer. ESR spectra were recorded with a JEOL JES-FA100N spectrometer with X-band microwave. Sample solutions were charged in a 2.5mmϕ sample tube. Magnetic field was calibrated with the Mn^{2+}/MgO standard. Absolute luminescence quantum yields and fluorescence lifetimes were measured using Hamamatsu Photonics Quantaurus QY C11347 and Quantaurus Tau C11367, respectively. Elemental analysis was conducted using Perkin-Elmer 2400 II CHNS/O. High-resolution mass spectrometry was conducted using JEOL JMS S-3000 MALDI-TOF MS spectrometer. DFT calculations were performed using Gaussian 16 Rev. C.02.

2. Synthesis of precursors with pyridine donors using pyridylboronic acid

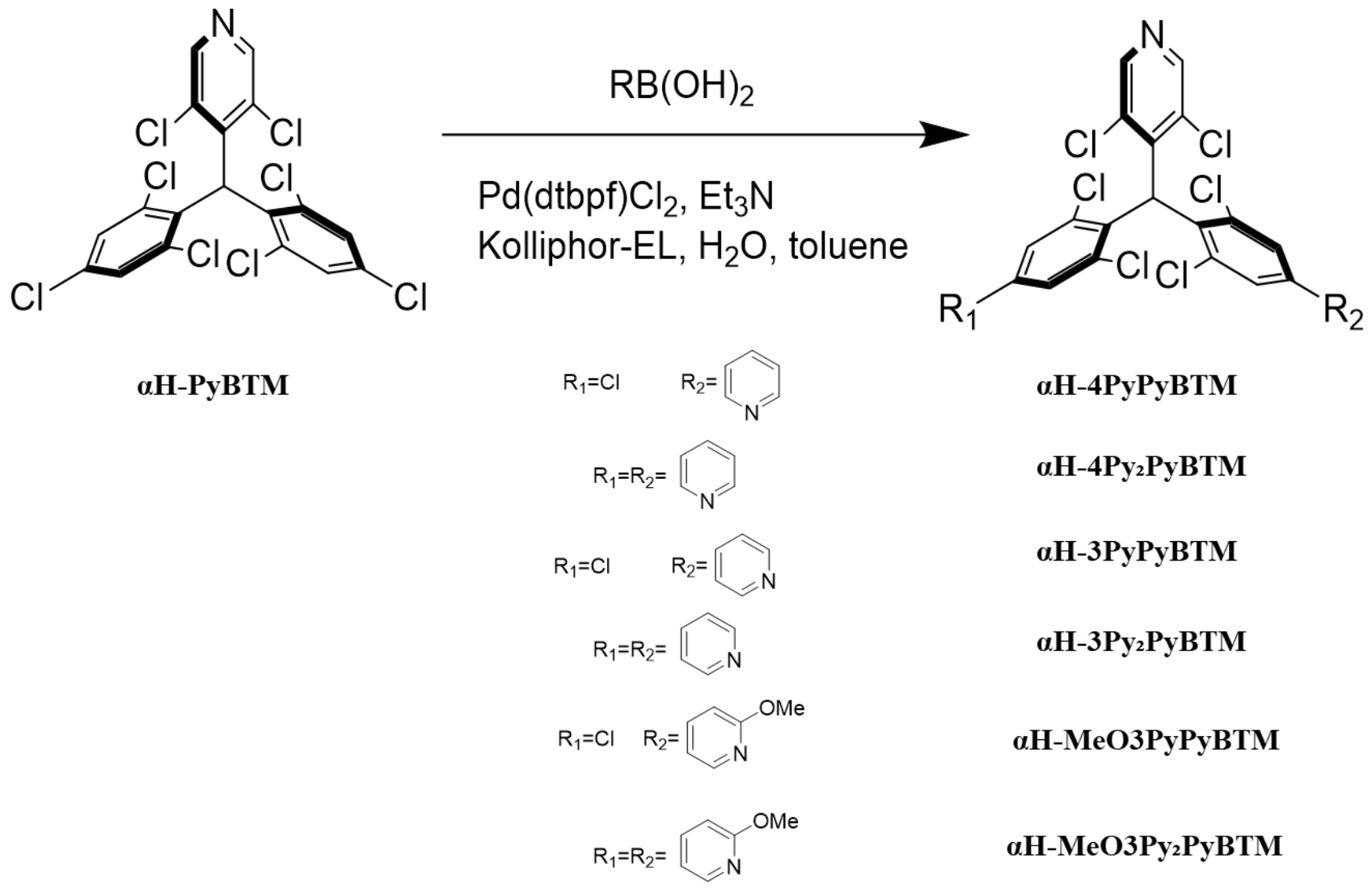

αH-4PyPyBTM and **αH-4Py$_2$PyBTM:** αH-PyBTM (520.9 mg, 1.0 mmol), 4-pyridylboronic acid (491.7 mg, 4.0 mmol), and Pd(dtbpf)Cl_2 (52.1 mg, 0.08 mmol) were placed in a Schlenk flask under an argon atmosphere. Then, a degassed emulsion (3.5 mL) of Kolliphor EL 2 wt% mixed with water and toluene in a 9:1 ratio was added, and the mixture was heated to 70 °C. Finally, degassed triethylamine (0.85 mL) was added, and the reaction mixture was heated overnight at 70 °C. The reaction mixture was cooled to room temperature, filtered through a celite using chloroform solvent, and the solvent was evaporated. The product was purified by silica gel column chromatography ($CHCl_3$) and the product recrystallized from CH_2Cl_2 and hexane to yield αH-4PyPyBTM (82.4 mg, 0.146 mmol, 15%) and αH-4Py$_2$PyBTM (6.5 mg, 0.011 mmol, 1.1%).

αH-4PyPyBTM: HRMS (MALDI-TOF MS positive mode) m/z: $[MH]^+$ calcd for $C_{23}H_{12}Cl_7N_2^+$ 560.8815; found 559.8819. ^{1}H and ^{13}C NMR (below).

αH-4Py$_2$PyBTM: HRMS (MALDI-TOF MS positive mode) *m/z*: $[MH]^+$ calcd for $C_{28}H_{16}Cl_6N_3^+$ 603.9470; found 603.9462. ^{1}H and ^{13}C NMR (below).

αH-3PyPyBTM and **αH-3Py$_2$PyBTM:** αH-PyBTM (520.9 mg, 1.0 mmol), 3-pyridylboronic acid (491.8 mg, 4.0 mmol), and Pd(dtbpf)Cl_2 (52.1 mg, 0.08 mmol) were placed in a Schlenk flask under an argon atmosphere. Then, a degassed emulsion (3.5 mL) of Kolliphor EL 2 wt% mixed with water and toluene in a 9:1 ratio was added, and the

mixture was heated to 70 °C. Finally, degassed triethylamine (0.85 mL) was added, and the reaction mixture was heated overnight at 70 °C. The reaction mixture was cooled to room temperature, filtered through a celite using chloroform solvent, and the solvent was evaporated. The product was purified by silica gel column chromatography ($CHCl_3$) and the product recrystallized from CH_2Cl_2 and hexane to yield αH-3PyPyBTM (184 mg, 0.327 mmol, 33%) and αH-3Py_2PyBTM (26.2 mg, 0.043 mmol, 4.3%).

αH-3PyPyBTM: HRMS (MALDI-TOF MS positive mode) m/z: $[MH]^+$ calcd for $C_{23}H_{12}Cl_7N_2^+$ 560.8815; found 559.8817. ^1H and ^{13}C NMR (below).

αH-3Py₂PyBTM: HRMS (MALDI-TOF MS positive mode) *m*/*z*: $[MH]^+$ calcd for $C_{28}H_{16}Cl_6N_3^+$ 603.9470; found 603.9471. ^1H and ^{13}C NMR (below).

αH-MeO3PyPyBTM and **αH-MeO3Py_2PyBTM:** αH-PyBTM (376.8 mg, 0.724 mmol), 2-methoxy-3-pyridylboronic acid (458.7 mg, 3 mmol), and Pd(dtbpf)Cl_2 (39.1 mg, 0.06 mmol) were placed in a Schlenk flask under an argon atmosphere. Then, a degassed emulsion (2.7 mL) of Kolliphor EL 2 wt% mixed with water and toluene in a 9:1 ratio was added, and the mixture was heated to 70 °C. Finally, degassed triethylamine (0.65 mL) was added, and the reaction mixture was heated overnight at 70 °C. The reaction mixture was cooled to room temperature, filtered through a celite using chloroform solvent, and the solvent was evaporated. The product was purified by silica gel column chromatography ($CHCl_3$). Subsequently, recrystallization of the product from CH_2Cl_2 and hexane yielded αH-MeO3PyPyBTM (crude yield 229.7 mg) and αH-MeO3Py_2PyBTM (95.4 mg, 0.143 mmol, 20%). Since impurities were still detected in αH-MeO3PyPyBTM, it was further purified by silica gel column chromatography and the product recrystallized from CH_2Cl_2 and hexane to yield 131.3 mg (0.221 mmol, 31%).

αH-MeO3PyPyBTM: HRMS (MALDI-TOF MS positive mode) m/z: $[MH]^+$ calcd for $C_{24}H_{14}Cl_7N_2O^+$ 590.8920; found 590.8922. ^1H and ^{13}C NMR (below).

αH-MeO3Py_2PyBTM: HRMS (MALDI-TOF MS positive mode) *m*/*z*: $[MH]^+$ calcd for $C_{30}H_{20}Cl_6N_3O_2^+$ 663.9681; found 663.9689. ^1H and ^{13}C NMR (below).

3. Synthesis of radicals with pyridine donors

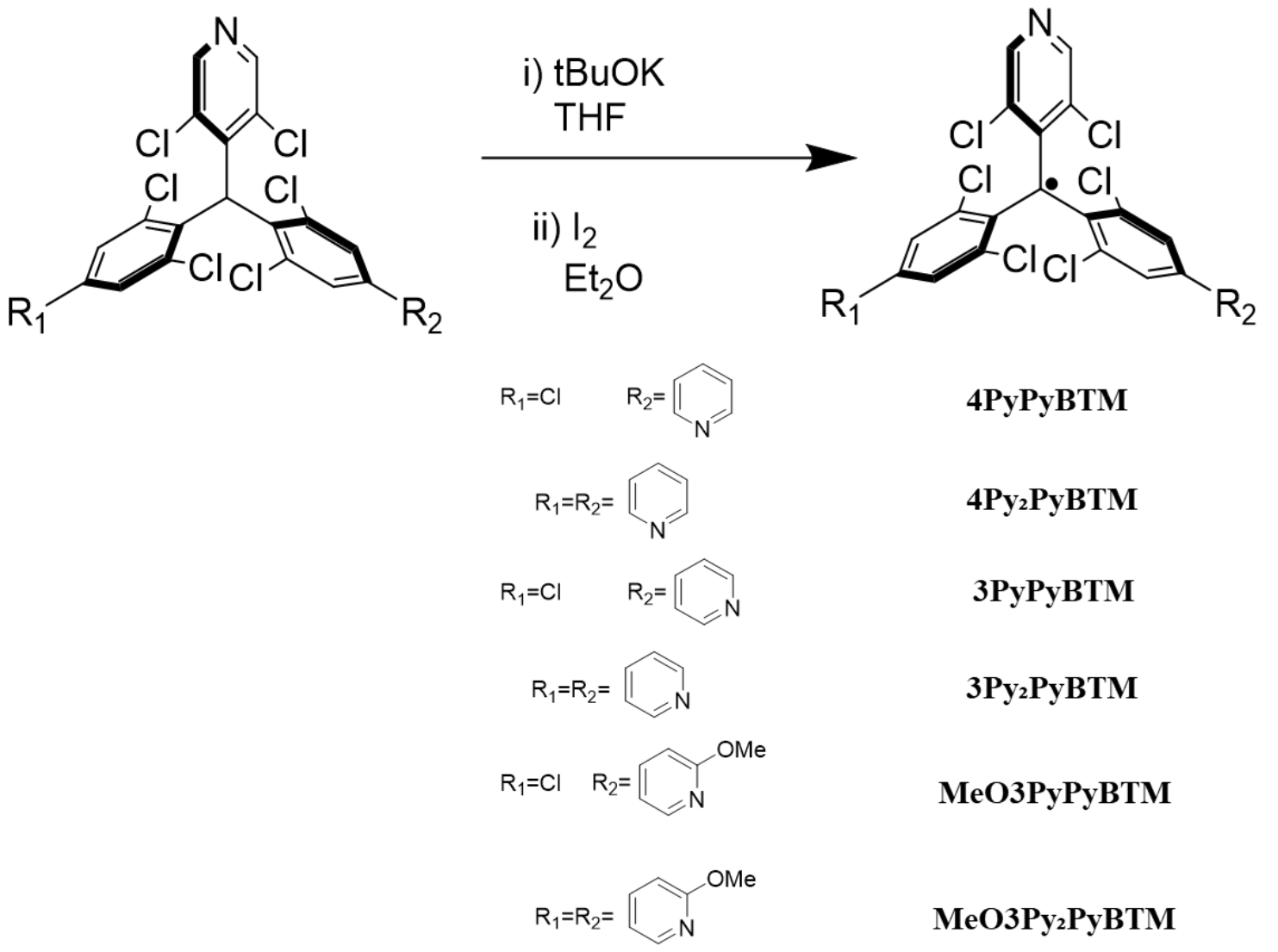

4PyPyBTM: Under an argon atmosphere, **αH-4PyPyBTM** (112.7 mg, 0.2 mmol) was dissolved in anhydrous THF (8.0 mL) and added to 0.3 mL of 1 M *t*BuOK in THF. The entire vessel was kept in the dark and stirred overnight. Then I_2 (304.5 mg, 1.22 mmol) dissolved in anhydrous Et_2O (16 mL) was added dropwise and the reaction kept in the dark and stirred for an additional 2.25 hours. After stirring, the I_2 in excess was reduced by washing the solution with $Na_2S_2O_3$ aq. The aqueous layer was then extracted with Et_2O, and the organic layer was dried with sodium sulfate. The resulting red solution was filtered, evaporated and purified by silica gel column chromatography ($CHCl_3$). Subsequent recrystallization of the product from CH_2Cl_2 and hexane yielded 4PyPyBTM (86.4 mg, 0.154 mmol, 77%). HRMS (MALDI-TOF MS positive mode) *m/z*: $[MH]^+$ calcd for $C_{23}H_{11}Cl_7N_2^+$ 559.8742; found 559.8736. Elemental analysis calcd for $C_{23}H_{10}Cl_7N_2$: C 49.11, H 1.79, N 4.98; found: C 49.20, H 1.49, N 4.95.

4Py₂PyBTM: Under an argon atmosphere, **αH-4Py₂PyBTM** (17.0 mg, 0.030 mmol) was dissolved in anhydrous THF (1.1 mL) and added to 0.1 mL of 1 M *t*BuOK in THF. The entire vessel was kept in the dark and stirred overnight. Then I_2 (41.0 mg, 0.16 mmol), dissolved in dry Et_2O (2.2 mL) was added dropwise and the reaction kept in the dark and stirred for an additional 2.25 hours. After stirring, the I_2 in excess was reduced by washing the solution with $Na_2S_2O_3$ aq. The aqueous layer was then extracted with Et_2O, and the organic layer was dried over sodium sulfate. The resulting red solution was filtered, evaporated and purified by silica gel column chromatography ($CHCl_3$). Subsequent recrystallization of the product from H_2O and acetone yielded **4Py₂PyBTM** (7.1 mg, 0.013 mmol, 42%).

HRMS (MALDI-TOF MS positive mode) *m/z*: $[MH]^+$ calcd for $C_{28}H_{15}Cl_6N_3^+$ 602.9392; found 602.9392. Elemental analysis calcd for $C_{28}H_{14}Cl_6N_3$: C 55.58, H 2.33, N 6.94; found: C 55.51, H 2.32, N 6.86.

3PyPyBTM: Under an argon atmosphere, **αH-3PyPyBTM** (112.7 mg, 0.2 mmol) was dissolved in anhydrous THF (8.0 mL), and added to 0.4 mL of 1 M *t*BuOK in THF. The entire vessel was kept in the dark and stirred overnight. Then I_2 (304.5 mg, 1.22 mmol) dissolved in anhydrous Et_2O (12 mL) was added dropwise and the reaction kept in the dark and stirred for an additional 2.25 hours. After stirring, the I_2 in excess was reduced by washing the solution with $Na_2S_2O_3$ aq. The aqueous layer was then extracted with Et_2O, and the organic layer was dried with sodium sulfate. The resulting red solution was filtered, evaporated and purified by silica gel column chromatography ($CHCl_3$). Subsequent recrystallization of the product from CH_2Cl_2 and hexane yielded 3PyPyBTM (100.1 mg, 0.178 mmol, 89 %). HRMS (MALDI-TOF MS positive mode) *m/z*: $[MH]^+$ calcd for $C_{23}H_{11}Cl_7N_2^+$ 559.8742; found 559.8738. Elemental analysis calcd for $C_{23}H_{10}Cl_7N_2$: C 49.11, H 1.79, N 4.98; found: C 49.14, H 1.66, N 4.92.

3Py₂PyBTM: Under an argon atmosphere, αH-3PyPyBTM(19.0mg, 0.03 mmol) was dissolved in anhydrous THF (1.2 mL), and added to 0.1 mL of 1 M *t*BuOK in THF. The entire vessel was kept in the dark and stirred overnight. Then I_2 (46.0mg, 0.18 mmol) dissolved in anhydrous Et_2O (2.5 mL) was added dropwise and the reaction kept in the dark and stirred for an additional 2.25 hours. After stirring, the I_2 in excess was reduced by washing the solution with $Na_2S_2O_3$ aq. The aqueous layer was then extracted with Et_2O, and the organic layer was dried with sodium sulfate. The resulting red solution was filtered, evaporated and purified by silica gel column chromatography ($CHCl_3$). Subsequent recrystallization of the product from H_2O and acetone yielded $3Py_2PyBTM$ (8.7 mg, 0.014 mmol, 48 %). HRMS (MALDI-TOF MS positive mode) *m/z*: $[MH]^+$ calcd for $C_{28}H_{15}Cl_6N_3^+$ 602.9397; found 602.9383. Elemental analysis calcd for $C_{28}H_{14}Cl_6N_3$: C 55.58, H 2.33, N 6.94; found: C 55.93, H 2.03, N 6.87.

MeO3PyPyBTM: Under an argon atmosphere, αH-MeO3PyPyBTM (41.7 mg,0.07 mmol) was dissolved in anhydrous THF (2.5 mL), and added to 0.14 mL of 1 M *t*BuOK in THF. The entire vessel was kept in the dark and stirred overnight. Then I_2 (103.0 mg, 0.41 mmol) dissolved in anhydrous Et_2O (5.6 mL) was added dropwise and the reaction kept in the dark and stirred for an additional 2.25 hours. After stirring, the I_2 in excess was reduced by

washing the solution with $Na_2S_2O_3$ aq. The aqueous layer was then extracted with Et_2O, and the organic layer was dried with sodium sulfate. The resulting red solution was filtered, evaporated and purified by silica gel column chromatography ($CHCl_3$). Subsequent recrystallization of the product from H_2O and acetone yielded 3PyPyBTM (33.7 mg, 0.057 mmol, 81 %). HRMS (MALDI-TOF MS positive mode) *m/z*: $[M]^+$ calcd for $C_{24}H_{12}Cl_7N_2O^+$588.8769; found 588.8770. Elemental analysis calcd for $C_{24}H_{12}Cl_7N_2O$: C 48.65, H 2.04, N 4.73; found: C 48.82, H 1.83, N 4.80.

MeO3Py₂PyBTM: Under an argon atmosphere, αH-MeO3Py$_2$PyBTM (46.6 mg,0.07 mmol) was dissolved in anhydrous THF (2.8 mL), and added to 0.14 mL of 1 M *t*BuOK in THF. The entire vessel was kept in the dark and stirred overnight. Then I_2 (103 mg,0.41 mmol) dissolved in anhydrous Et_2O (9.0 mL) was added dropwise and the reaction kept in the dark and stirred for an additional 2.25 hours. After stirring, the I_2 in excess was reduced by washing the solution with $Na_2S_2O_3$ aq. The aqueous layer was then extracted with Et_2O, and the organic layer was dried with sodium sulfate. The resulting red solution was filtered, evaporated and purified by silica gel column chromatography ($CHCl_3$). Subsequent recrystallization of the product from CH_2Cl_2 and hexane yielded MeO3Py$_2$PyBTM (46.0 mg, 0.069 mmol).; HRMS (MALDI-TOF MS positive mode) *m/z*: $[M]^+$ calcd for $C_{30}H_{18}Cl_6N_3O_2^+$ 661.9530; found 661.9521.; Elemental analysis calcd for $C_{30}H_{18}Cl_6N_3O_2$: C 54.17, H 2.73, N 6.32; found: C 53.98, H 2.56, N 6.10.

4. ^{1}H NMR titration method

1. Precursor (*ca.* 2×10^{-6} mol) was dissolved in 600 μL of $CDCl_3$ and ^{1}H NMR was measured.
2. An acid (0.5 equiv.) in methanol was added and solvent in the ^{1}H NMR was evaporated in vacuo.
3. The residue was dissolved in 600 μL of $CDCl_3$ and ^{1}H NMR was measured.

Steps 2 and 3 were repeated.

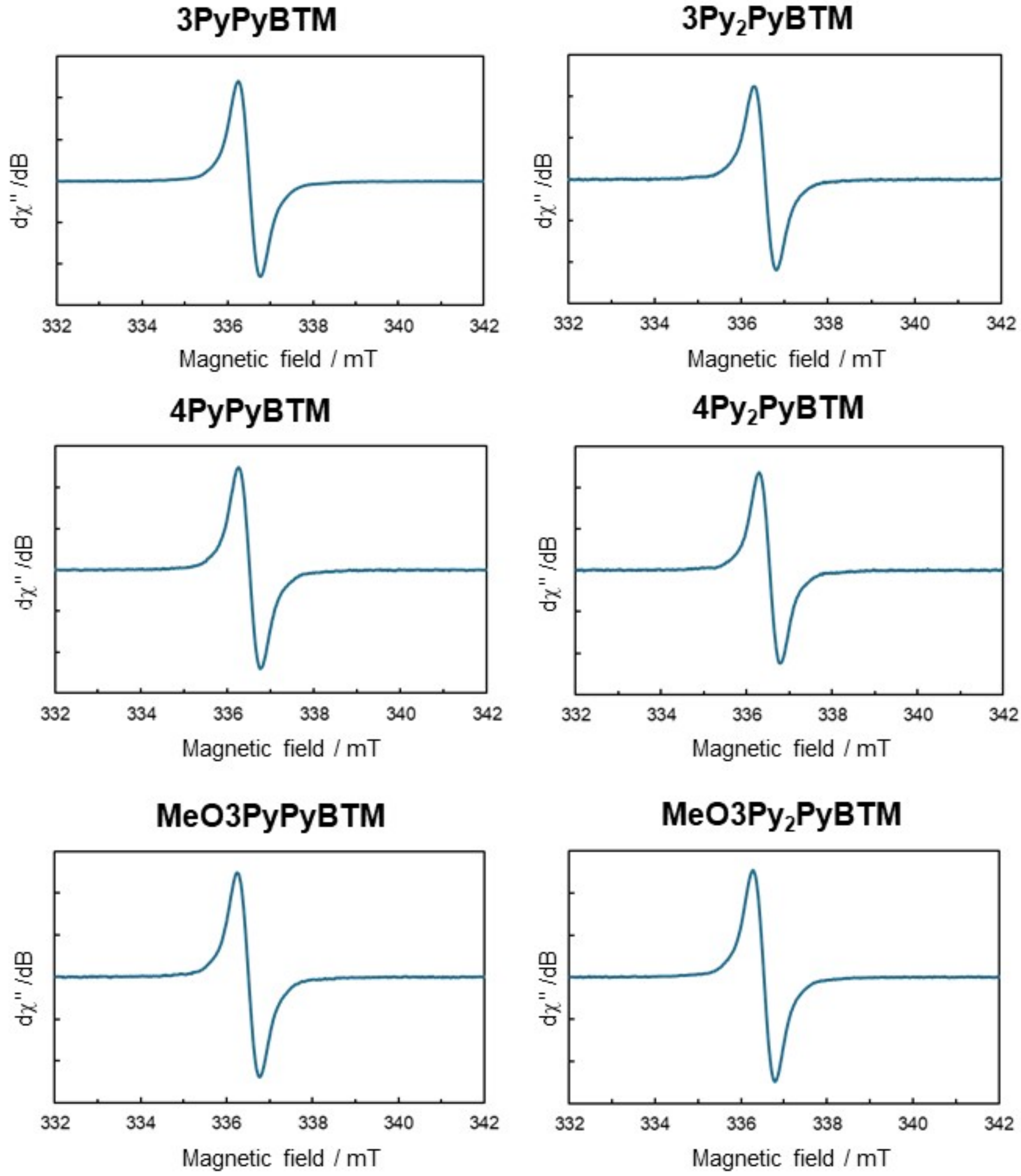

Figure S1. ESR spectra of the radicals in toluene. The *g* factor of the radical was *g* = 2.003. Spin concentration of the radicals in toluene (2.5−6.7×10−5 M) was estimated by comparing the value of twice-integration of the signal intensity with that of the reference sample (TEMPO in toluene; 4.0×10^{-5} M). The existence of *S* = 1/2 spin on one radical molecule was confirmed.

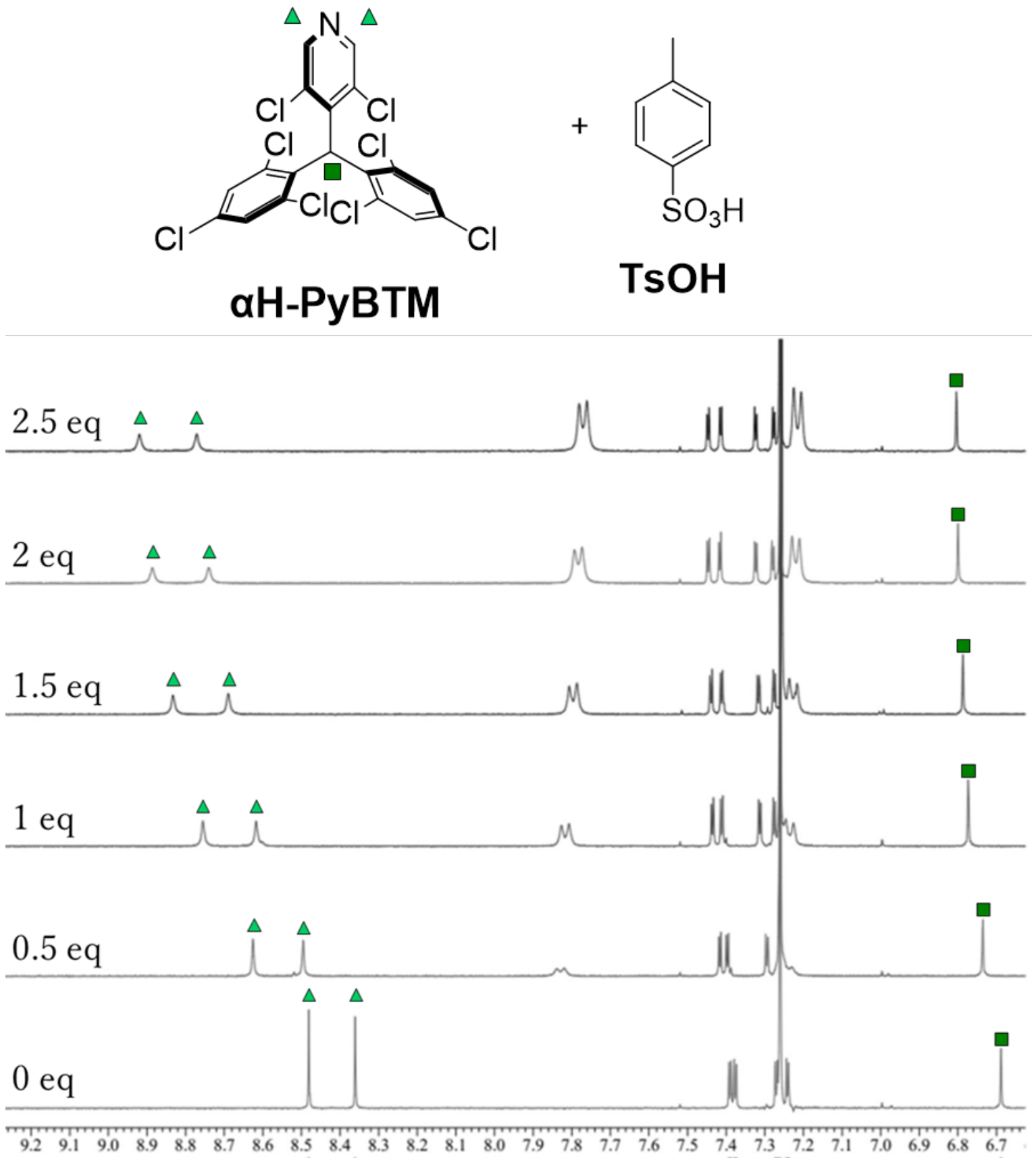

Figure S2. ^{1}H NMR spectral changes of αH-PyBTM by addition of TsOH.

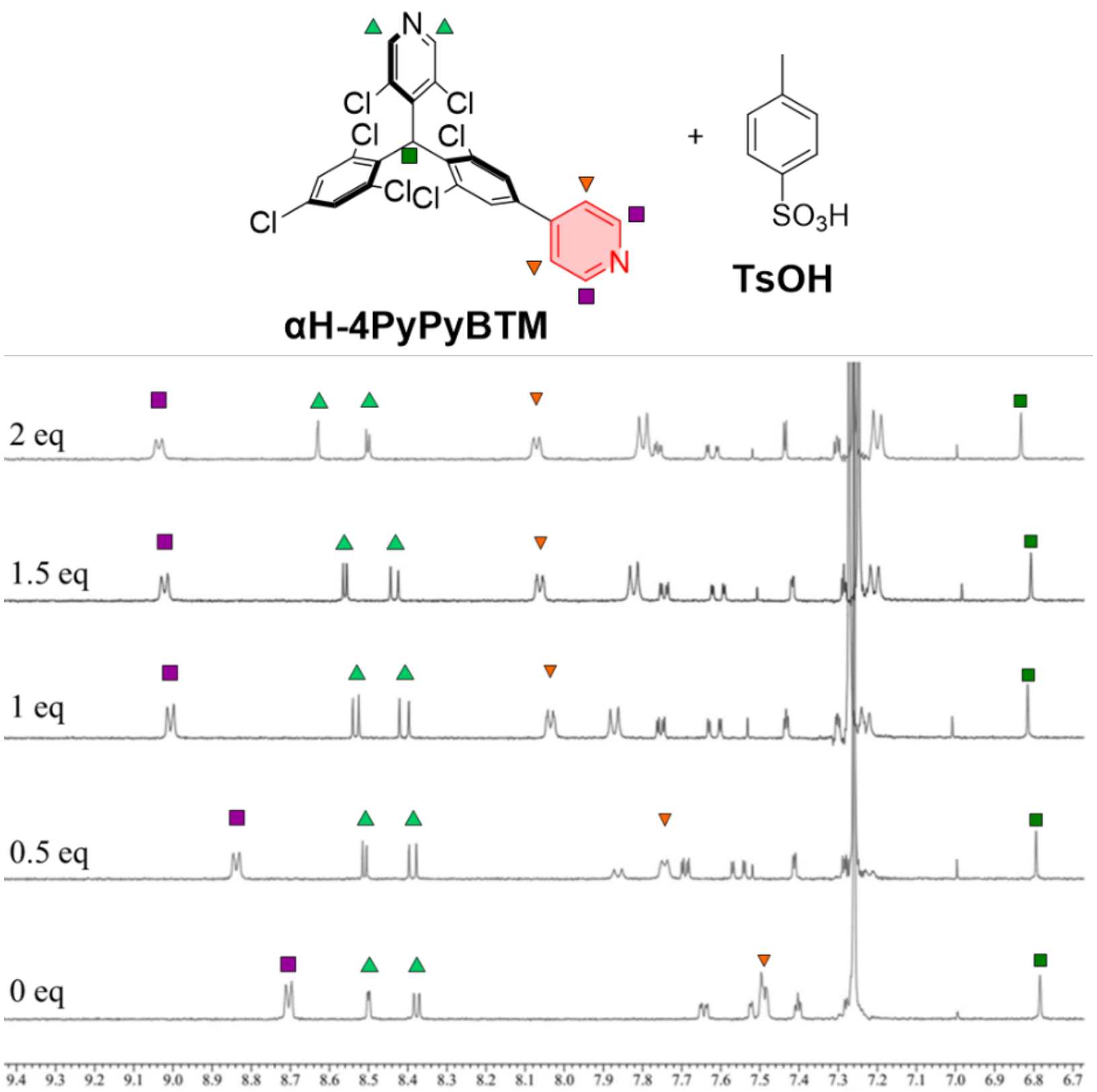

Figure S3. ^{1}H NMR spectral changes of αH-4PyPyBTM by addition of TsOH.

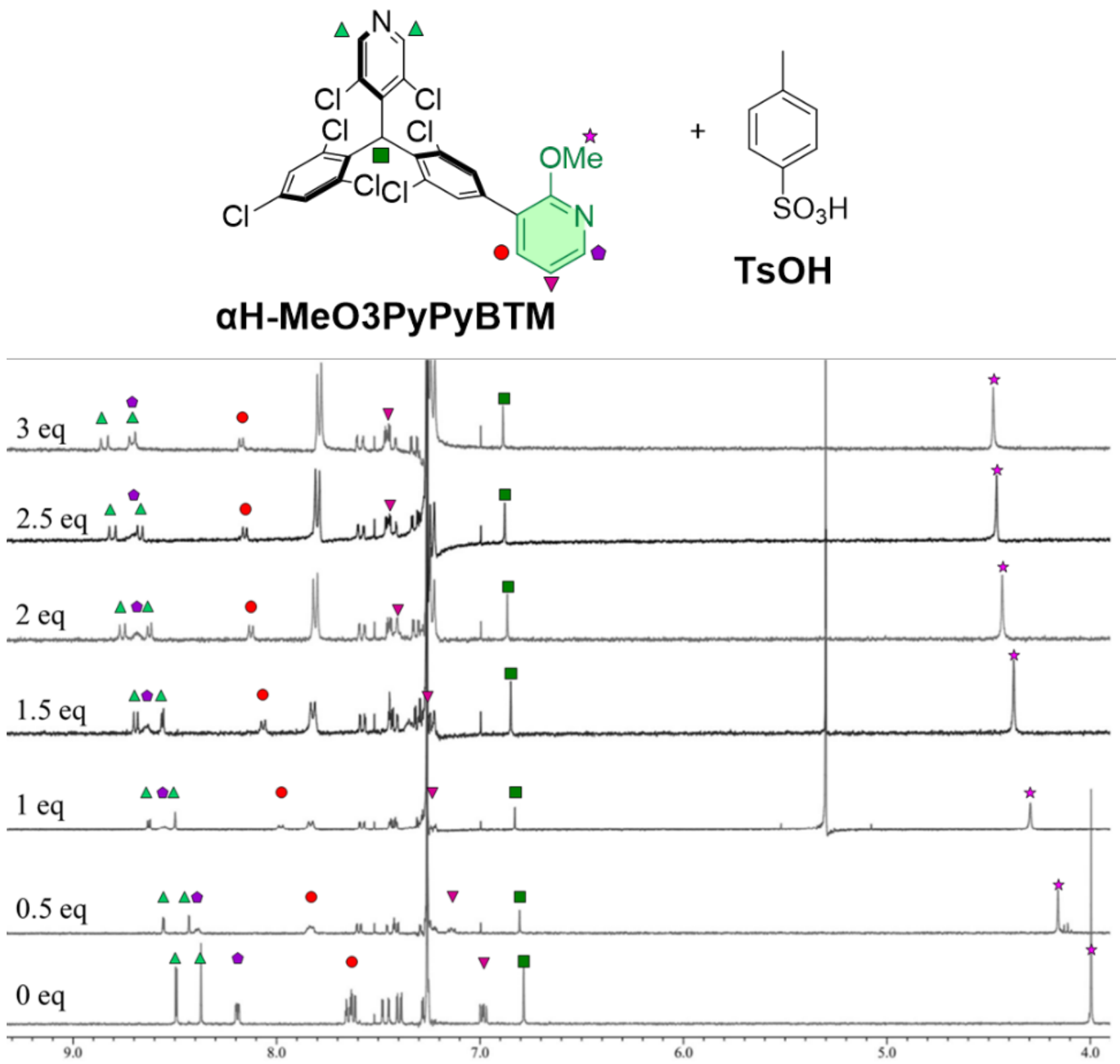

Figure S4. ^{1}H NMR spectral changes of αH-MeO3PyPyBTM by addition of TsOH.

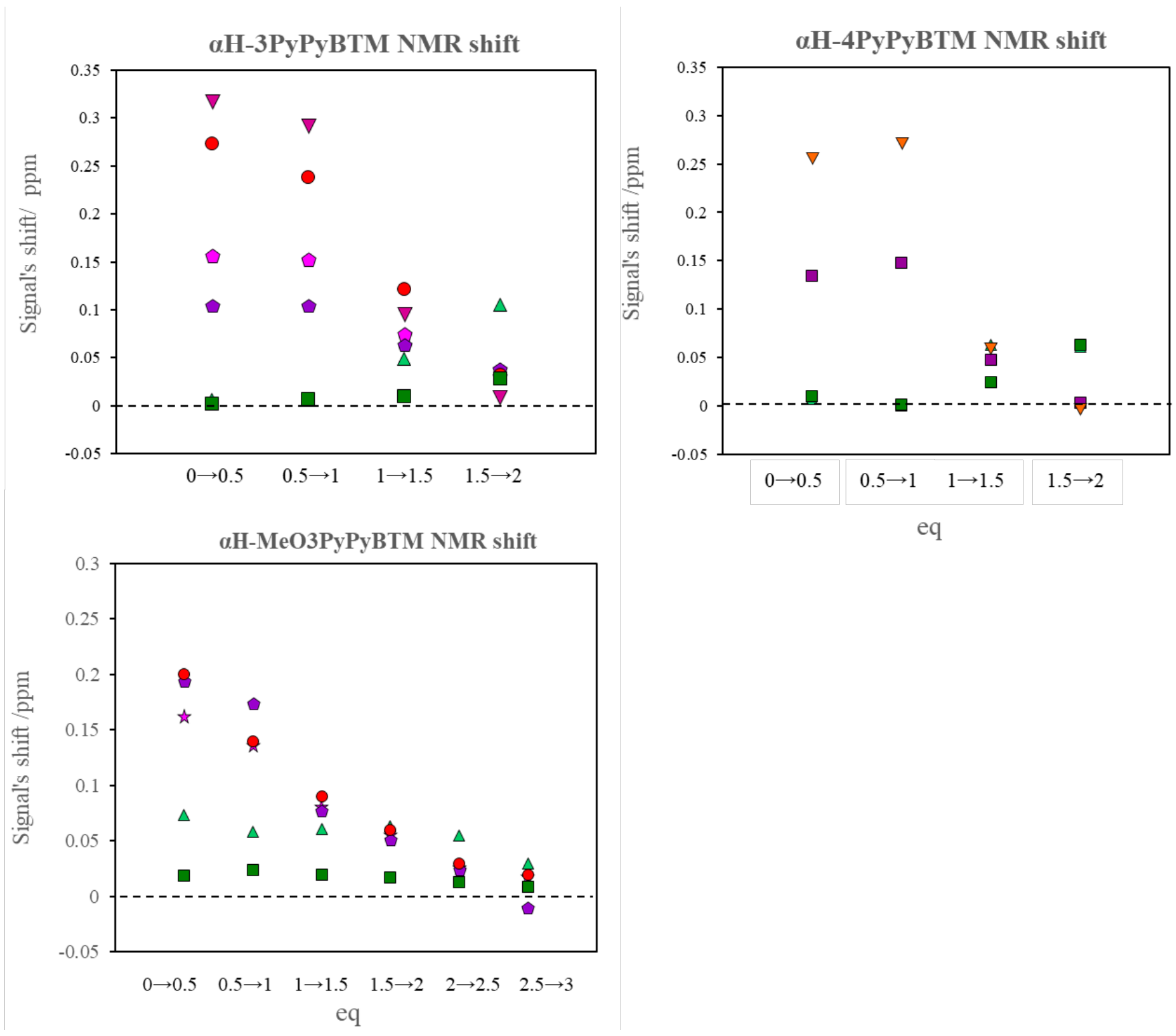

Figure S5. Values of signal's shifts between two spectra.

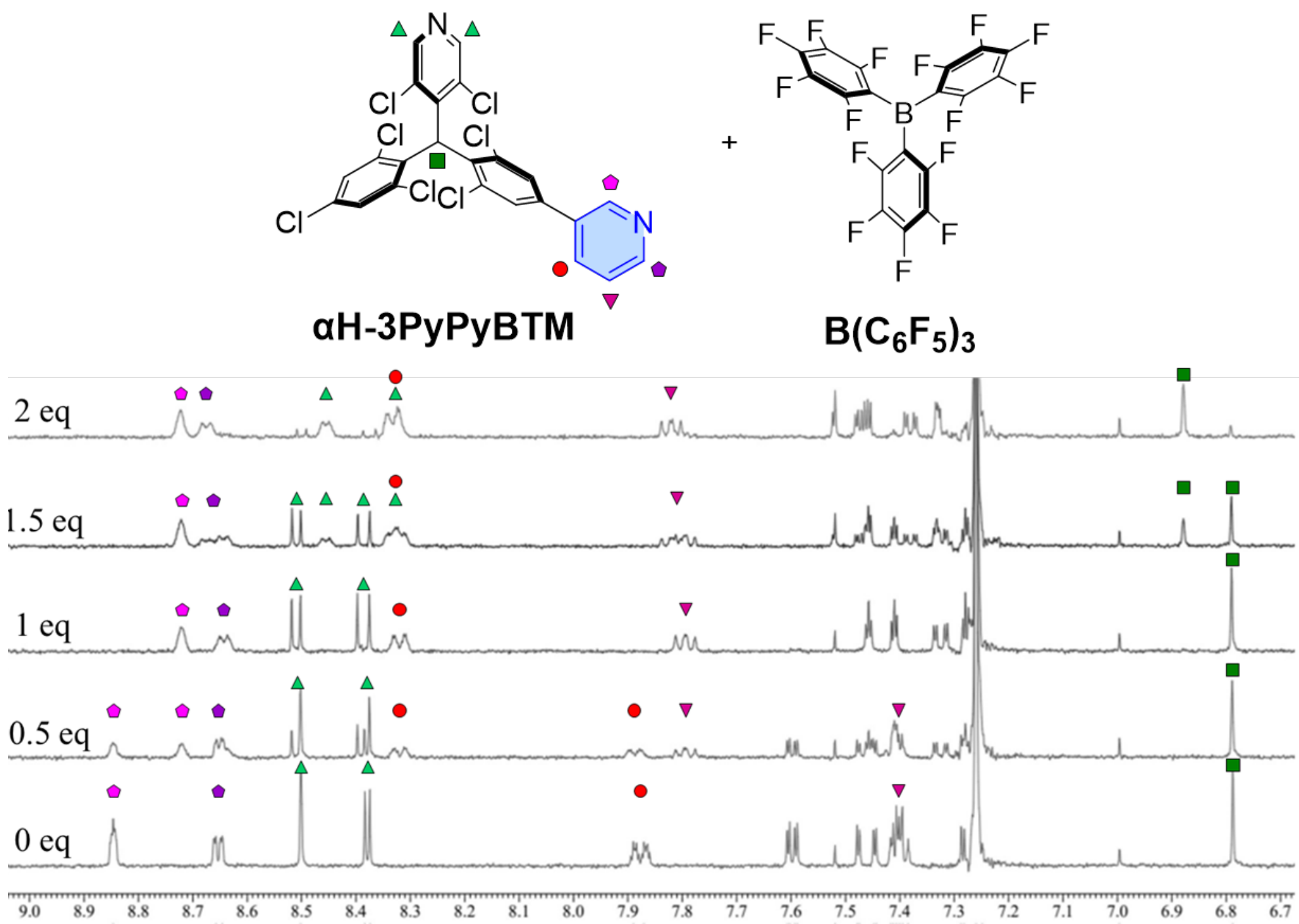

Figure S6. ^{1}H NMR spectral changes of αH-3PyPyBTM by addition of $B(C_6F_5)_3$.

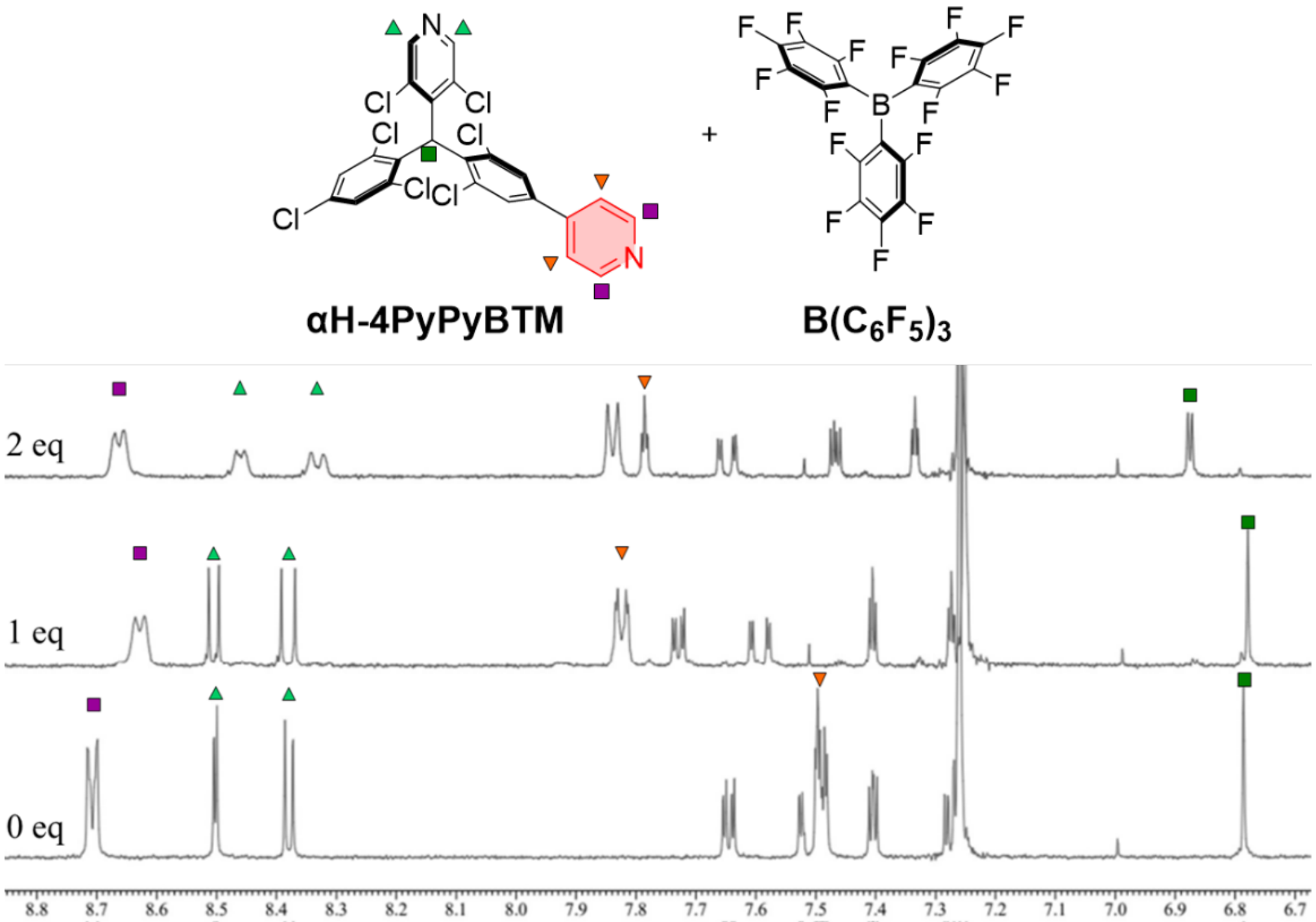

Figure S7. ^{1}H NMR spectral changes of αH-4PyPyBTM by addition of $B(C_6F_5)_3$.

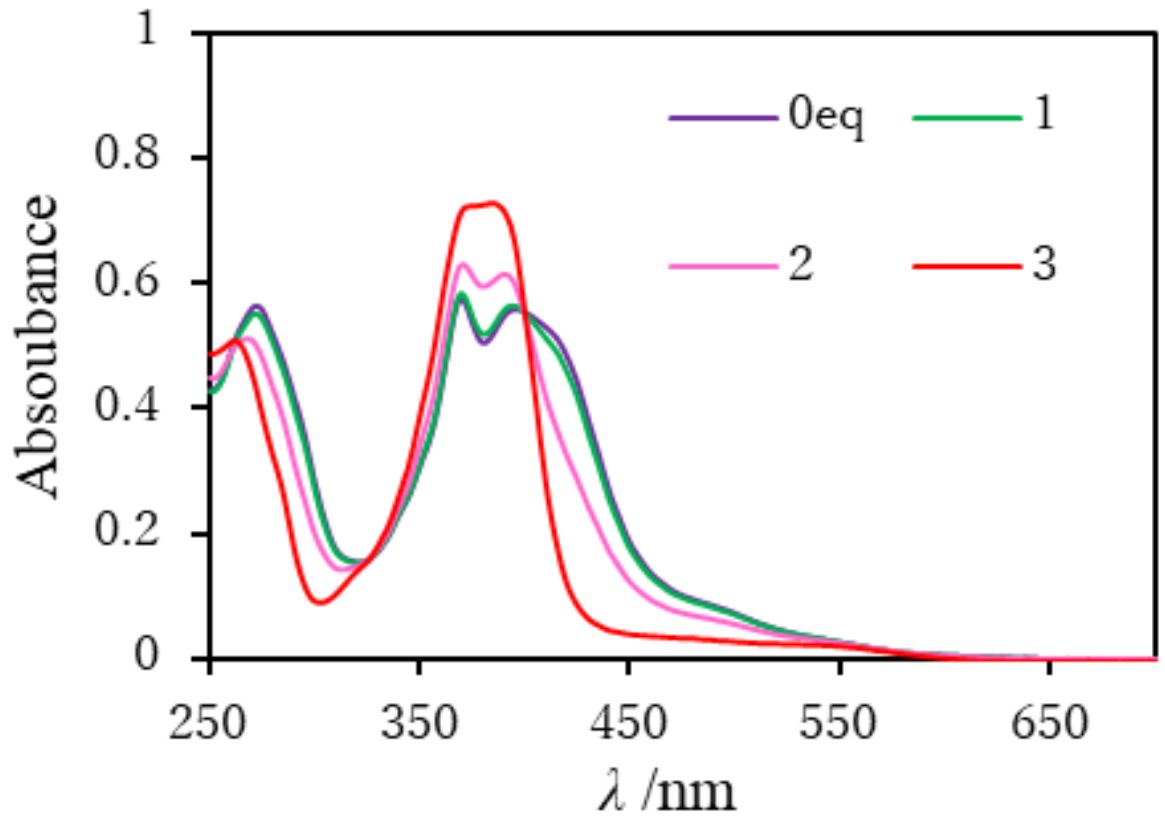

Figure S8. Absorption changes of 4PyPyBTM by addition of Et_3N after TsOH titration in chloroform.

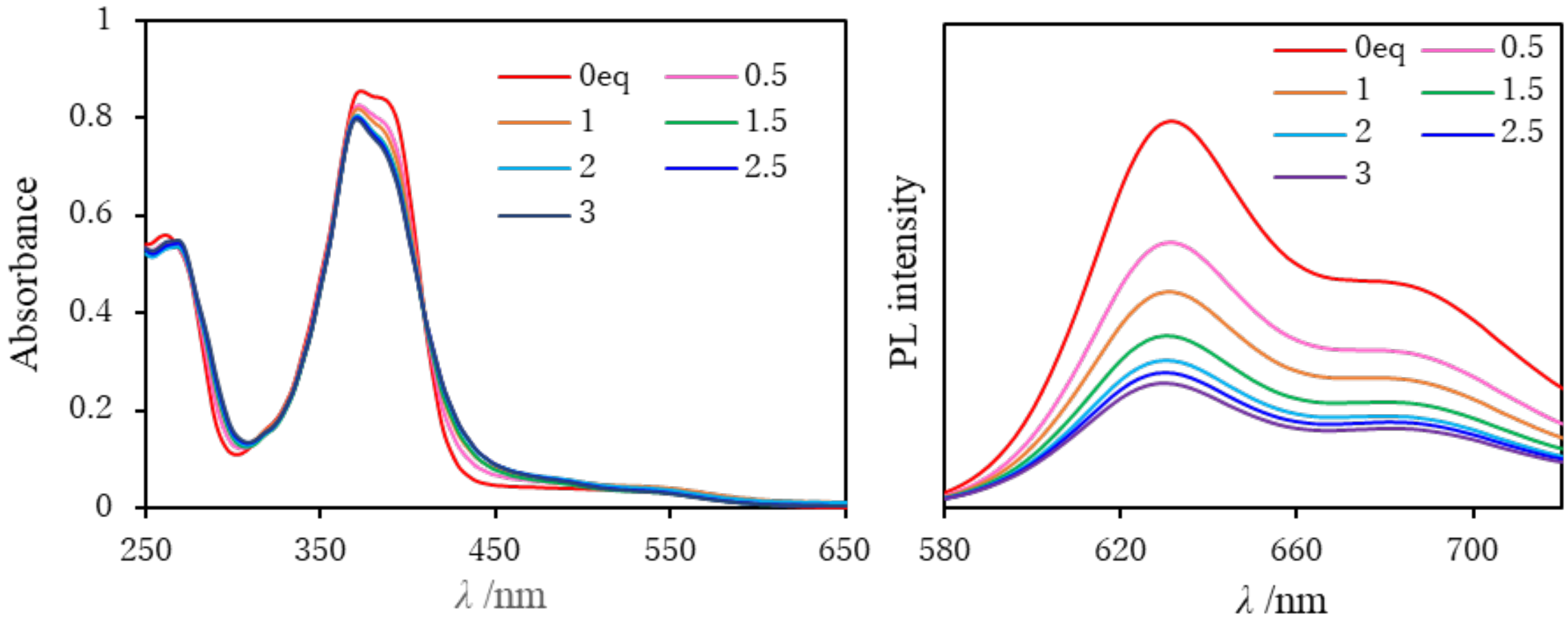

Figure S9. Absorption and emission changes of 3PyPyBTM (λ_{ex} = 373 nm) by addition of TsOH in chloroform.

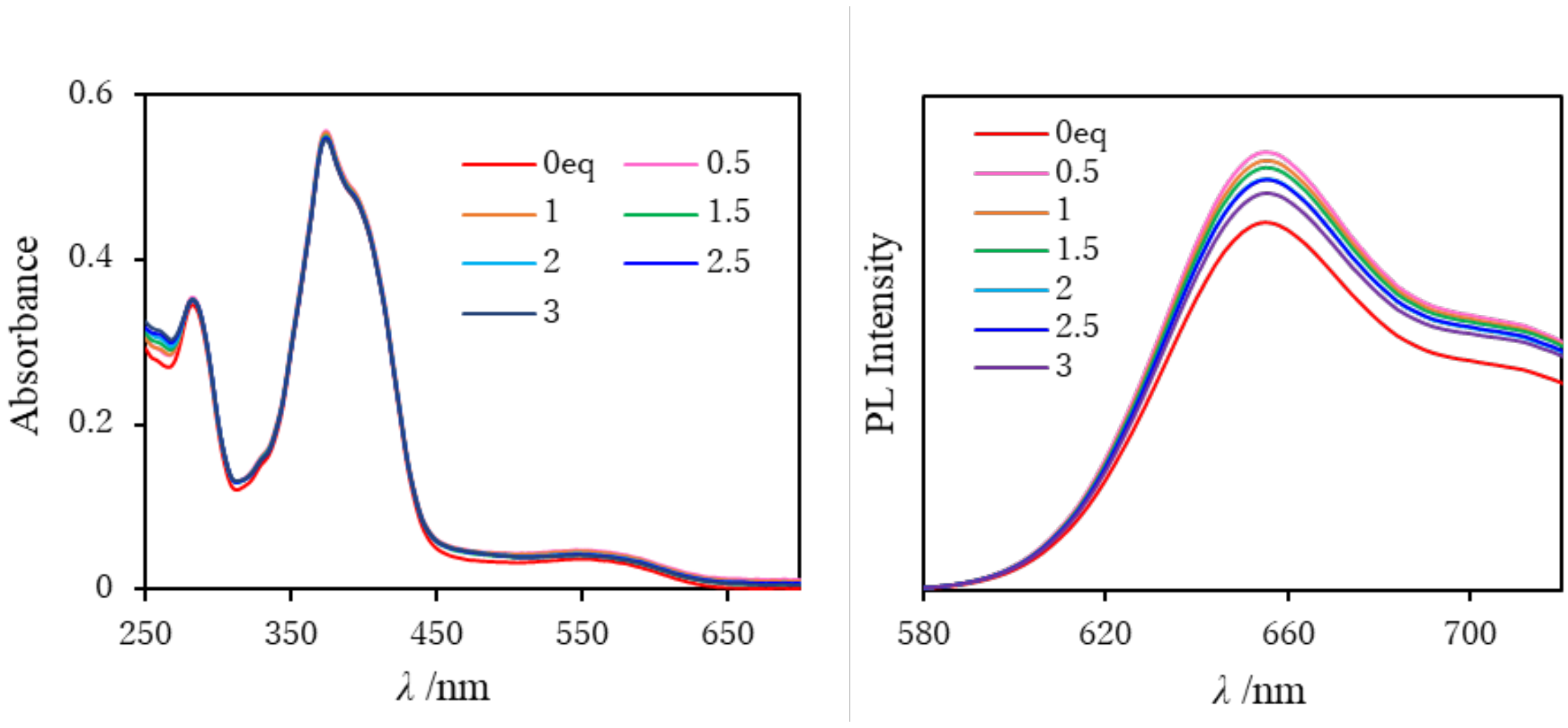

Figure S10. Absorption and emission changes of MeO3PyPyBTM (λ_{ex} = 374 nm) by addition of TsOH in chloroform.

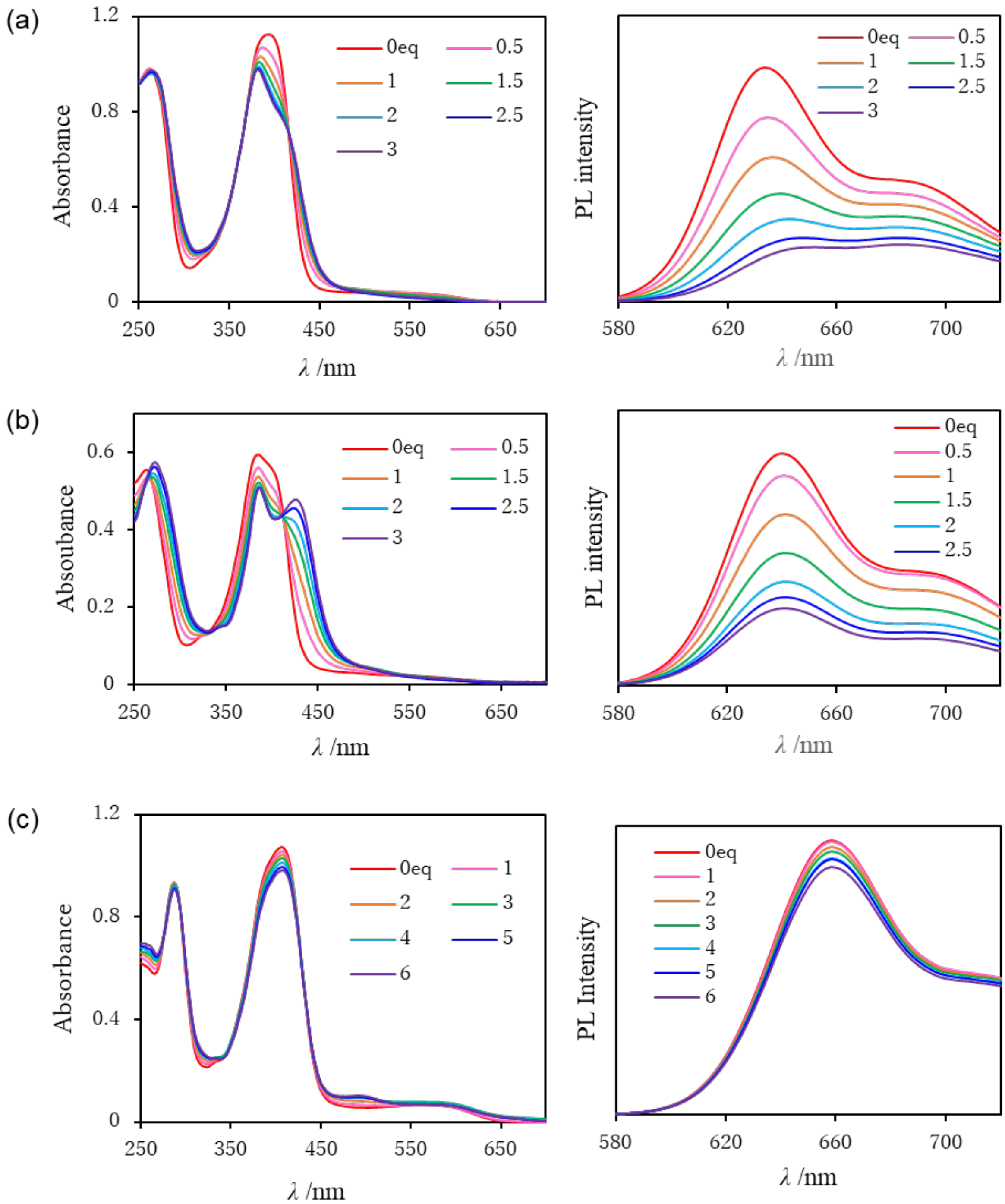

Figure S11. Absorption and emission changes of (a) $3Py_2PyBTM$ (λ_{ex} = 394 nm), (b) $4Py_2PyBTM$(λ_{ex} = 385 nm), and (c) $MeO3Py_2PyBTM$ (λ_{ex} = 394 nm) by addition of TsOH in chloroform.

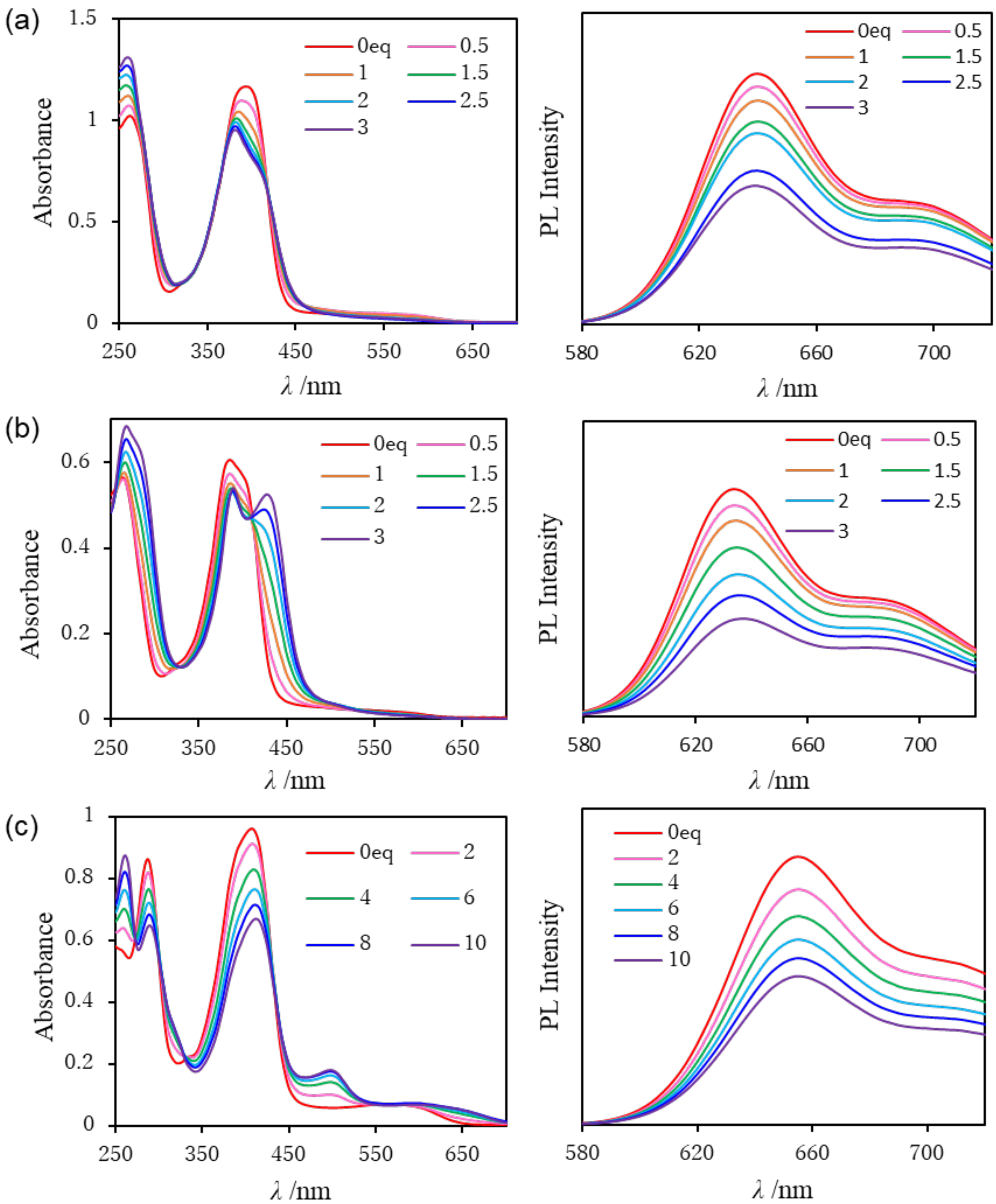

Figure S12. Absorption and emission changes of (a) 3Py$_2$PyBTM (λ_{ex} = 394 nm), (b) 4Py$_2$PyBTM (λ_{ex} = 385 nm), and (c) MeO3Py$_2$PyBTM (λ_{ex} = 394 nm) by addition of B(C_6F_5)$_3$ in chloroform.

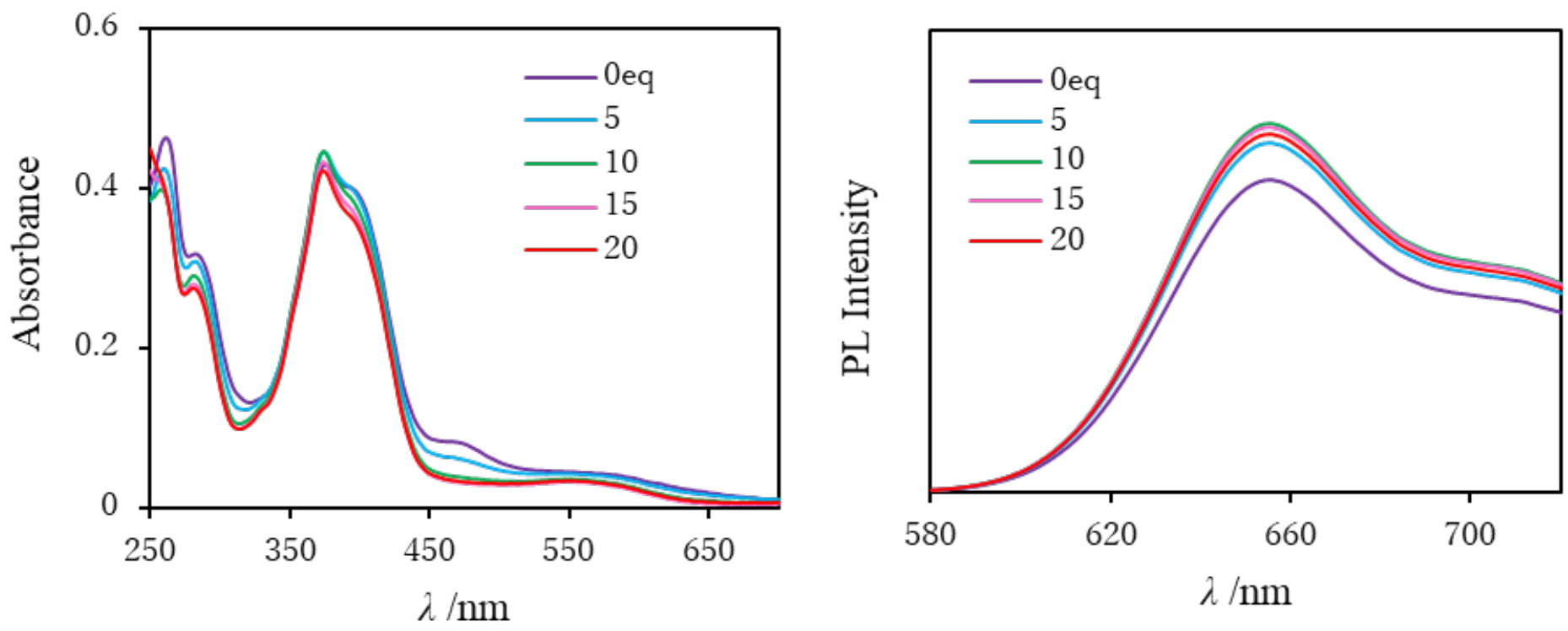

Figure S13. Absorption and emission changes of MeO3Py$_2$PyBTM by addition of Et_3N after $B(C_6F_5)_3$ titration in chloroform.

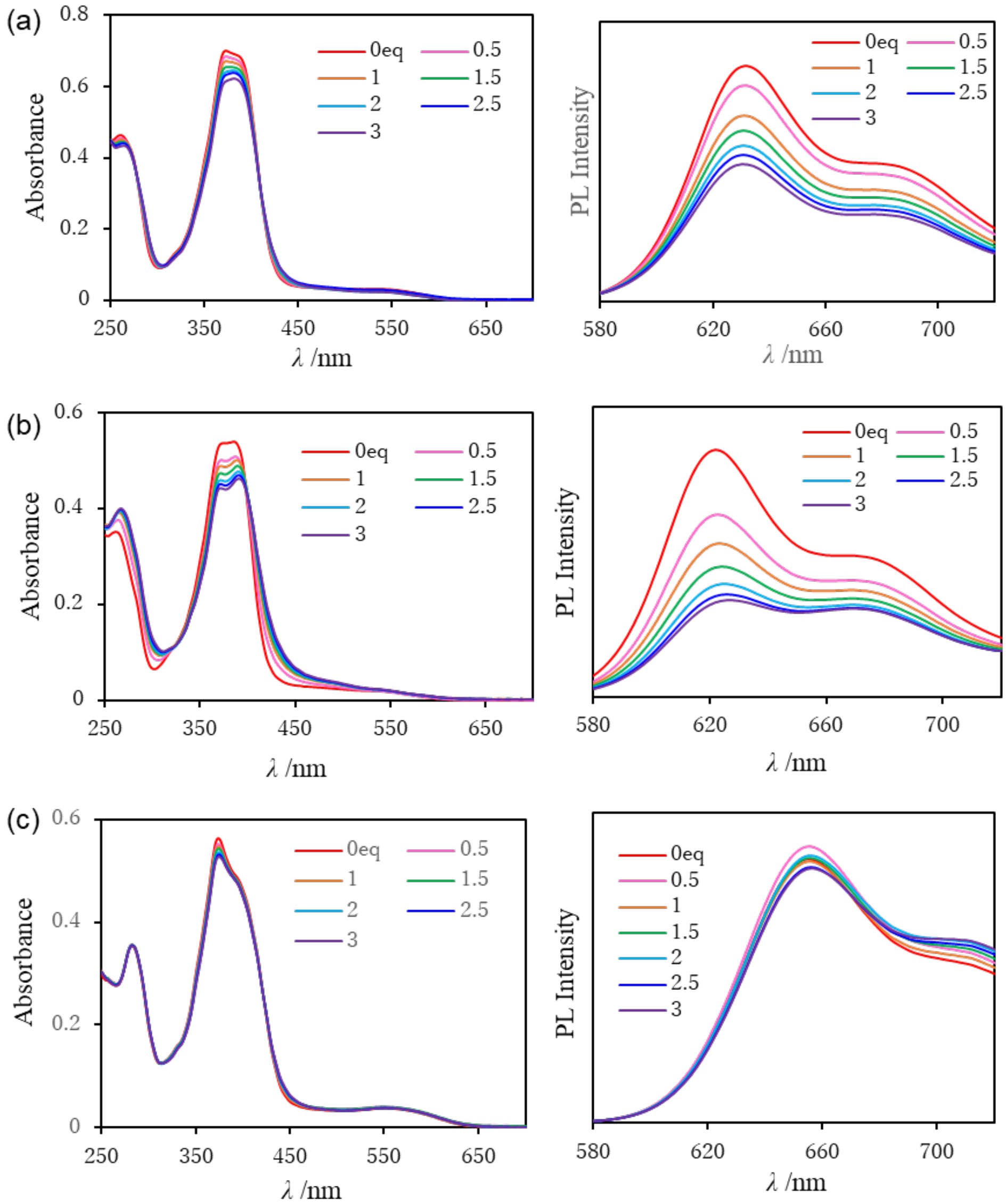

Figure S14. Absorption and emission changes of (a) 3PyPyBTM (λ_{ex} = 373 nm), (b) 4PyPyBTM (λ_{ex} = 372 nm), and (c) MeO3PyPyBTM (λ_{ex} = 374 nm) by addition of AgOTf in chloroform.

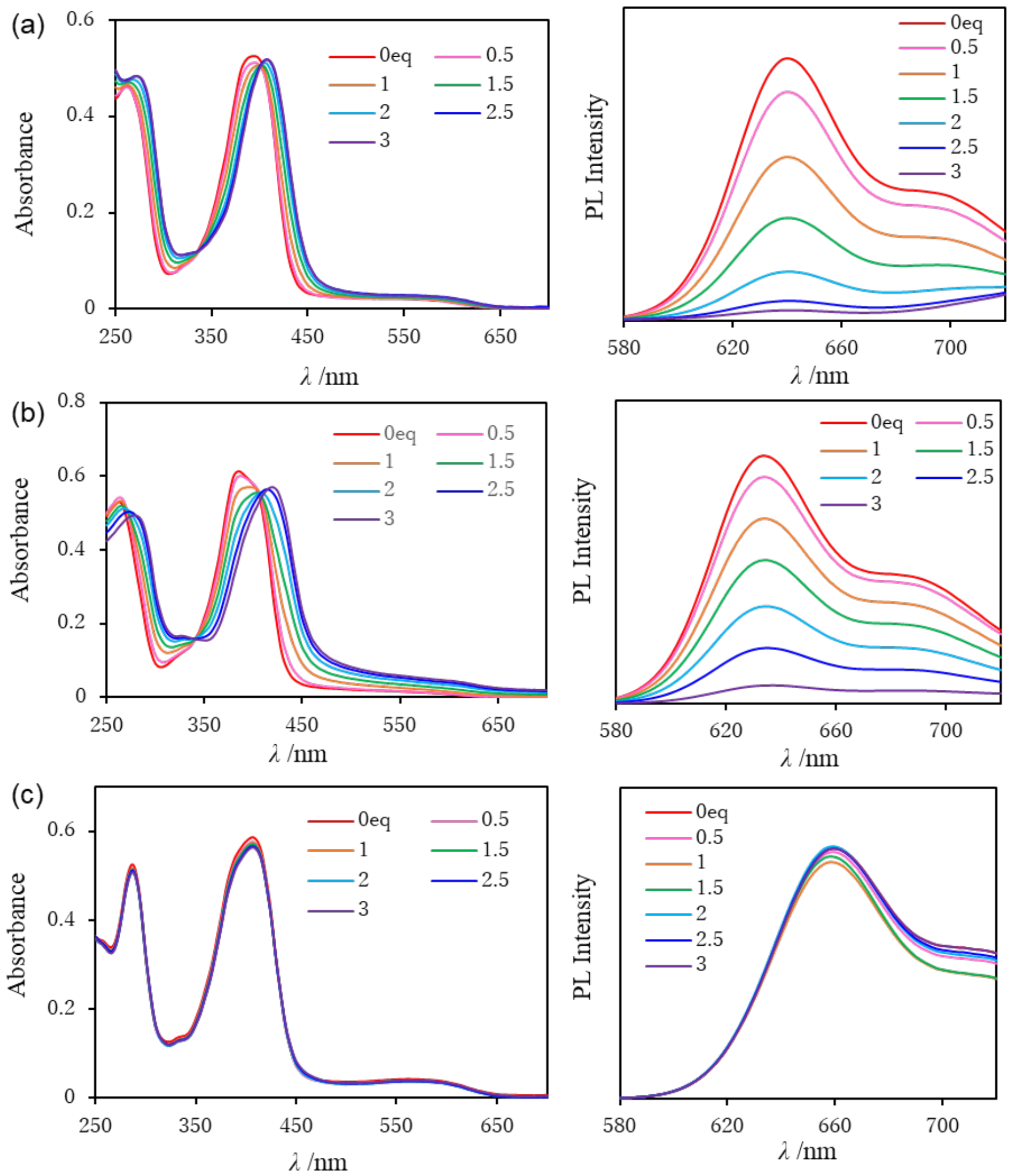

Figure S15. Absorption and emission changes of (a) $3Py_2PyBTM$ (λ_{ex} = 394 nm), (b) $4Py_2PyBTM$ (λ_{ex} = 385 nm), and (c) $MeO3Py_2PyBTM$ (λ_{ex} = 394 nm) by addition of AgOTf in chloroform.

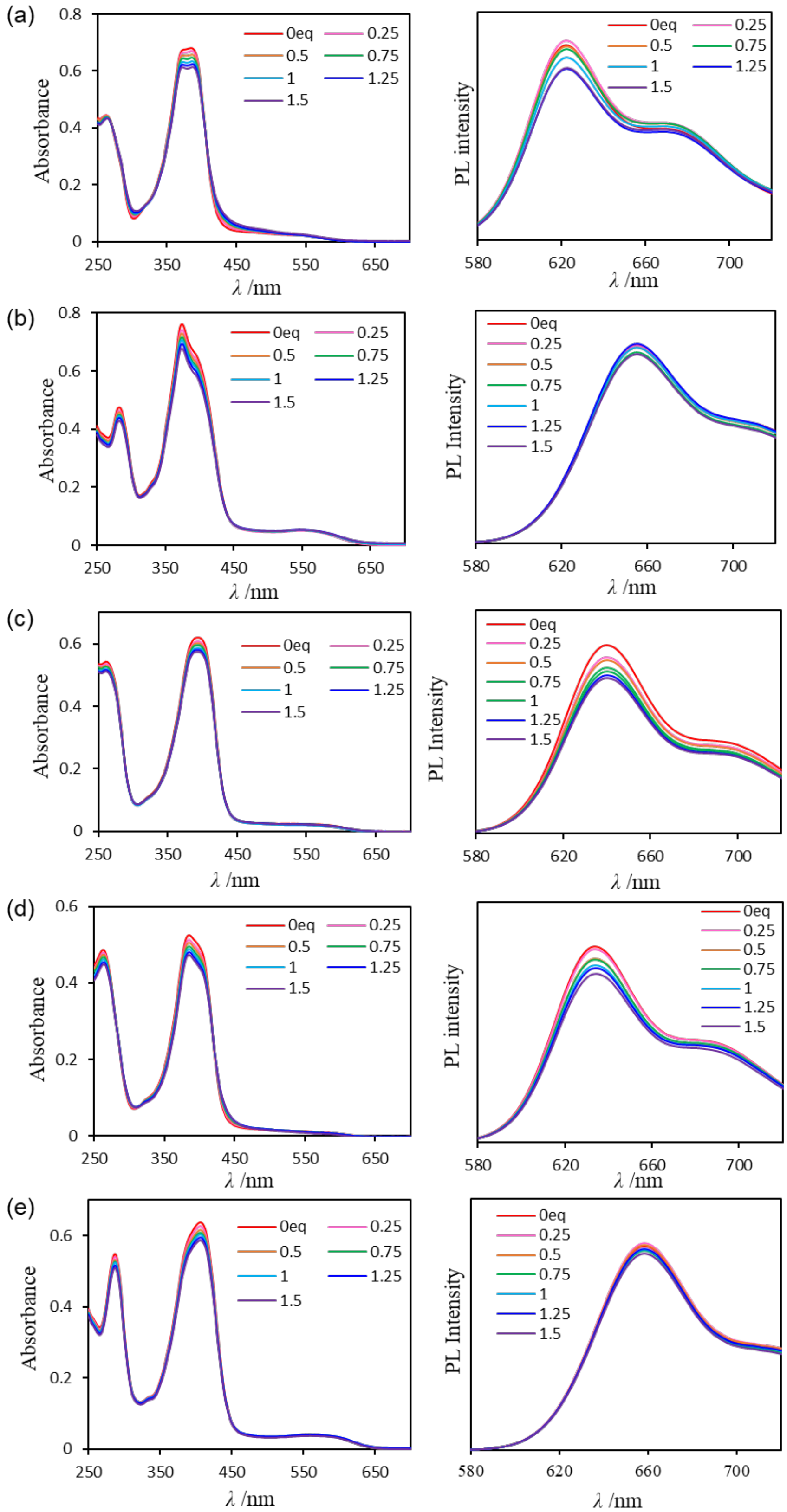

Figure S16. Absorption and emission changes of (a) 4PyPyBTM (λ_{ex} = 372 nm), (b) MeO3PyPyBTM (λ_{ex} = 374 nm), (c) 3Py$_2$PyBTM (λ_{ex} = 394 nm), (d) 4Py$_2$PyBTM (λ_{ex} = 385 nm), and (e) MeO3Py$_2$PyBTM (λ_{ex} = 394 nm) by addition of $Zn(OTf)_2$ in chloroform.

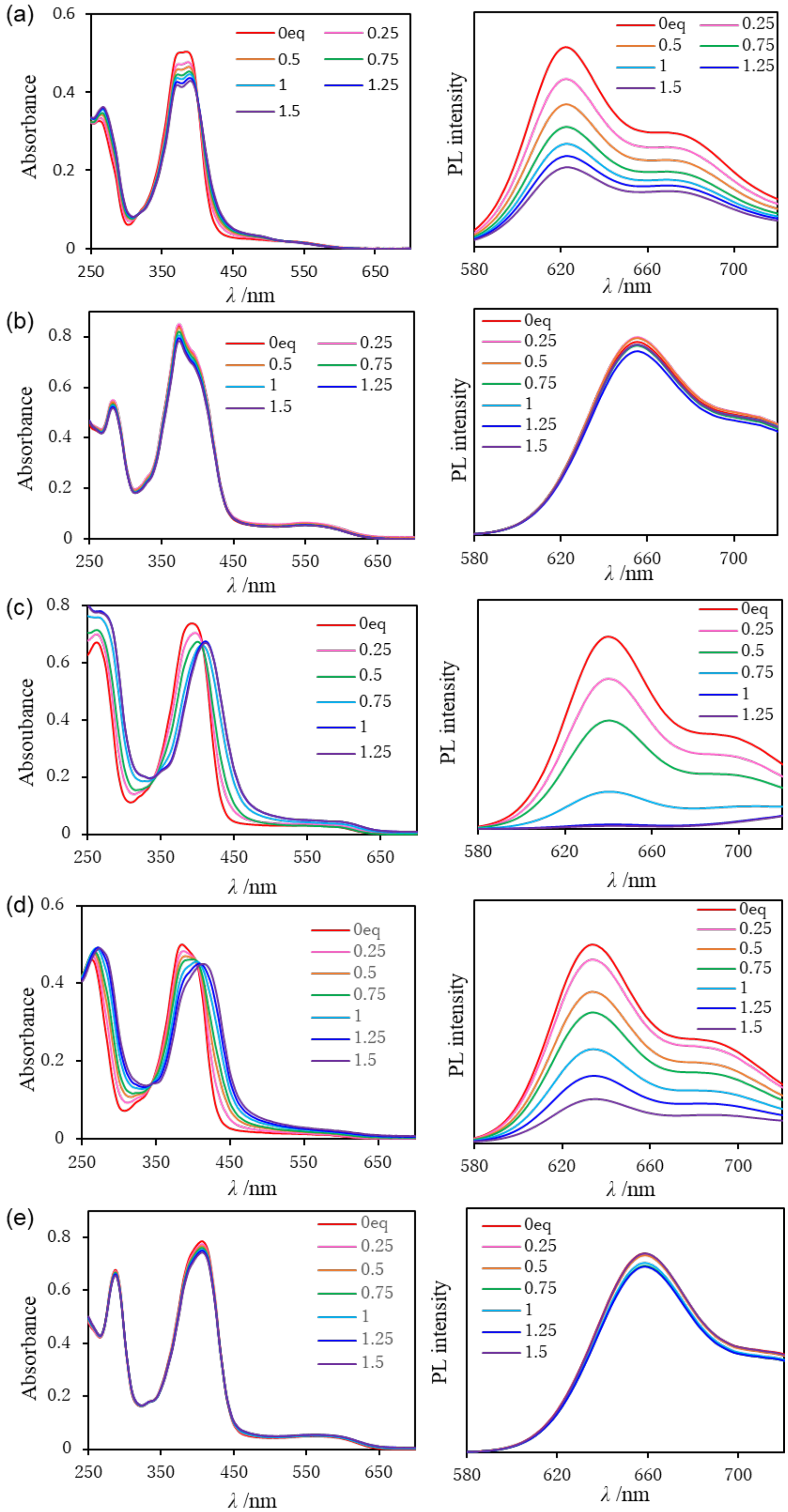

Figure S17. Absorption and emission changes of (a) 4PyPyBTM (λ_{ex} = 372 nm), (b) MeO3PyPyBTM (λ_{ex} = 374 nm), (c) 3Py$_2$PyBTM (λ_{ex} = 394 nm), (d) 4Py$_2$PyBTM (λ_{ex} = 385 nm), and (e) MeO3Py$_2$PyBTM (λ_{ex} = 394 nm) by addition of Cu^{II}(OTf)$_2$ in chloroform.

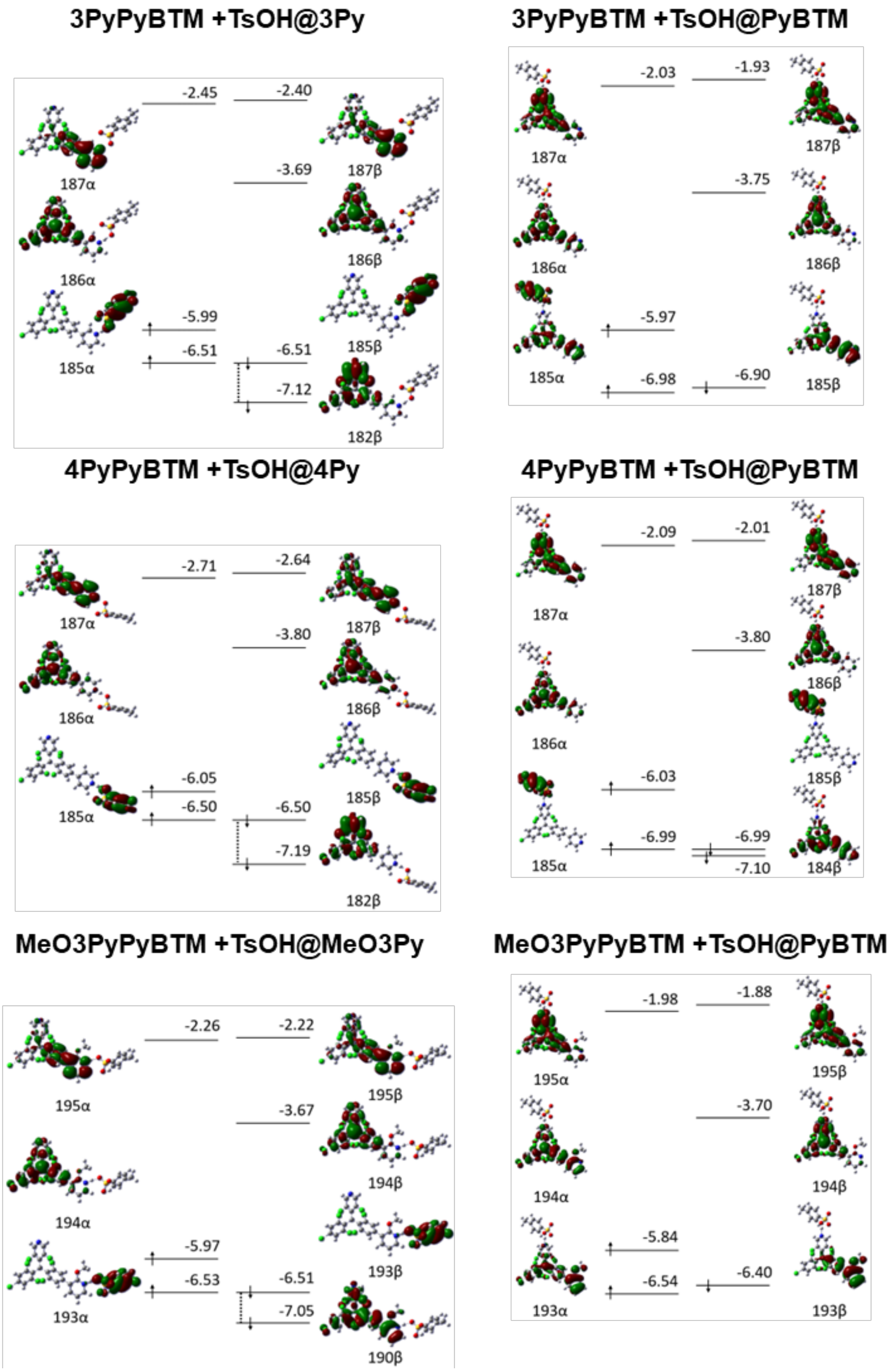

Figure S18. DFT calculated frontier orbitals of the radicals with TsOH optimized by DFT calculations at the UB3LYP/6-31G(d,p) level. The solvent effect of chloroform was considered using a polarizable continuum model (PCM). Orbital energies are shown in eV.

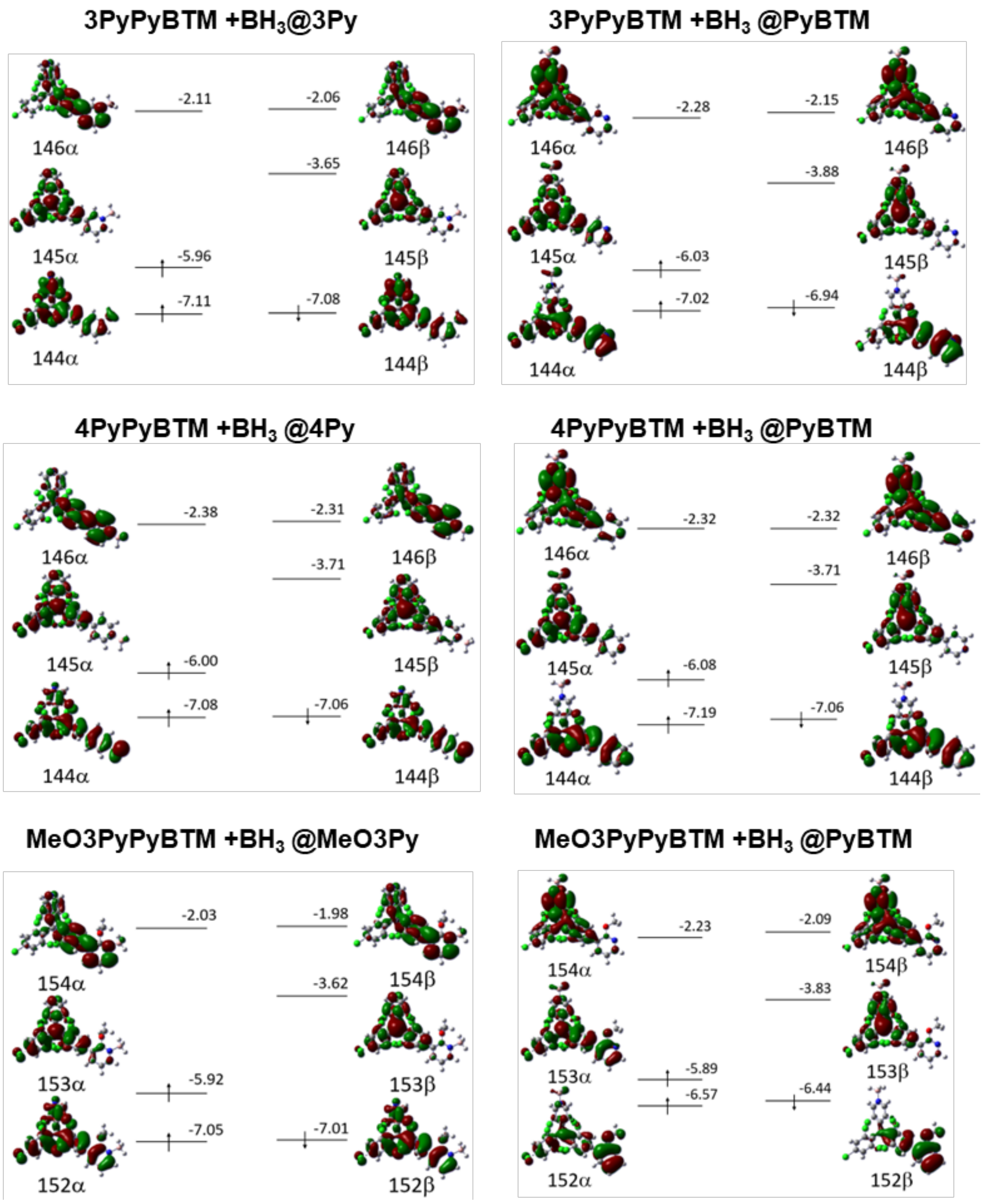

Figure S19. DFT calculated frontier orbitals of the radicals with BH_3 optimized by DFT calculations at the UB3LYP/6-31G(d,p) level. The solvent effect of chloroform was considered using a polarizable continuum model (PCM). Orbital energies are shown in eV.

Table S1 PLQYs of the radicals (Φ_f /%) in various solvents

	Cyclohexane	Toluene	$CHCl_3$	CH_2Cl_2	Acetone
**4PyPyBTM**	2.6	2.2	3.5	4.1	3.8
**4Py$_2$PyBTM**	3.6	4.7	4.9	5.4	5.4
**3PyPyBTM**	2.3	3.7	3.6	4.3	4.9
**3Py$_2$PyBTM**	3.2	5.5	4.9	5.5	6.0
**MeO3PyPyBTM**	2.1	4.5	4.6	6.1	6.3
**MeO3Py$_2$PyBTM**	2.4	5.4	5.3	6.5	6.8

Table S2 Fluorescence lifetimes of the radicals (τ /ns) in various solvents

	Cyclohexane	Toluene	$CHCl_3$	CH_2Cl_2	Acetone
**4PyPyBTM**	13.0	7.4	14.6	15.0	13.7
**4Py$_2$PyBTM**	14.5	12.0	16.0	16.2	15.4
**3PyPyBTM**	7.8	7.9	9.1	9.6	9.8
**3Py$_2$PyBTM**	8.7	9.8	10.2	10.5	10.6
**MeO3PyPyBTM**	4.5	6.1	6.2	7.0	7.0
**MeO3Py$_2$PyBTM**	5.2	6.8	6.8	7.3	7.5

Table S3 Fluorescence rate constants of the radicals (k_f /10^7 s^{-1}) in various solvents

	Cyclohexane	Toluene	$CHCl_3$	CH_2Cl_2	Acetone
**4PyPyBTM**	0.20	0.30	0.24	0.27	0.28
**4Py$_2$PyBTM**	0.25	0.39	0.31	0.33	0.35
**3PyPyBTM**	0.29	0.47	0.40	0.45	0.50
**3Py$_2$PyBTM**	0.37	0.56	0.48	0.52	0.57
**MeO3PyPyBTM**	0.47	0.74	0.74	0.87	0.90
**MeO3Py$_2$PyBTM**	0.46	0.79	0.78	0.89	0.91

Table S4 Non-radiative decay rate constants of the radicals (k_{nr} /10^7 s^{-1}) in various solvents

	Cyclohexane	Toluene	$CHCl_3$	CH_2Cl_2	Acetone
**4PyPyBTM**	7.5	13.2	6.6	6.4	7.0
**4Py$_2$PyBTM**	6.6	7.9	5.9	5.8	6.1
**3PyPyBTM**	12.5	12.2	10.6	10.0	9.7
**3Py$_2$PyBTM**	11.1	9.6	9.3	9.0	8.9
**MeO3PyPyBTM**	21.8	15.7	15.4	13.4	13.4
**MeO3Py$_2$PyBTM**	18.8	13.9	13.9	12.8	12.4

Table S5 DFT-calculated electron energies using UB3LYP/6-31G(d,p) in chloroform (Hartree)

	Energy	Enthalpy	Gibbs energy
**3PyPyBTM**	-4213.308007	-4213.012238	-4213.104407
**4PyPyBTM**	-4213.308058	-4213.012287	-4213.104460
**MeO3PyPyBTM**	-4327.844470	-4327.513529	-4327.611471
**TsOH**	-895.384757	-895.231101	-895.282565
**BH_3**	-26.615830	-26.585644	-26.607025
**3PyPyBTM+TsOH@3Py**	-5108.716641	-5108.265003	-5108.389471
**3PyPyBTM+TsOH@PyBTM**	-5108.711096	-5108.260073	-5108.385079
**4PyPyBTM+TsOH@4Py**	-5108.717983	-5108.266124	-5108.390357
**4PyPyBTM+TsOH@PyBTM**	-5108.711117	-5108.260217	-5108.385856
**MeO3PyPyBTM+TsOH@MeO3Py**	-5223.246348	-5222.759334	-5222.888027
**MeO3PyPyBTM+TsOH@PyBTM**	-5223.247750	-5222.761626	-5222.892756
**3PyPyBTM+BH_3@3Py**	-4239.979702	-4239.648991	-4239.746489
**3PyPyBTM+ BH_3@PyBTM**	-4239.974981	-4239.644426	-4239.741717
**4PyPyBTM+ BH_3@4Py**	-4239.981037	-4239.650287	-4239.747623
**4PyPyBTM+ BH_3@PyBTM**	-4239.974924	-4239.644386	-4239.741731
**MeO3PyPyBTM+ BH_3@MeO3Py**	-4354.499603	-4354.133920	-4354.237617
**MeO3PyPyBTM+ BH_3@PyBTM**	-4354.511712	-4354.145949	-4354.249549

Table S6 DFT-calculated acid-base reaction energies using UB3LYP/6-31G(d,p) in chloroform

	ΔE (Hartree)	ΔH (Hartree)	ΔG (Hartree)	ΔE (kJ/mol)	ΔH (kJ/mol)	ΔG (kJ/mol)
**3PyPyBTM+TsOH@3Py**	-0.023877	-0.021664	-0.002499	-62.7	-56.9	-6.6
**3PyPyBTM+TsOH@PyBTM**	-0.018332	-0.016734	0.001893	-48.1	-43.9	5.0
**4PyPyBTM+TsOH@4Py**	-0.025168	-0.022736	-0.003332	-66.1	-59.7	-8.7
**4PyPyBTM+TsOH@PyBTM**	-0.018302	-0.016829	0.001169	-48.1	-44.2	3.1
**MeO3PyPyBTM+TsOH@MeO3Py**	-0.017121	-0.014704	0.006009	-45.0	-38.6	15.8
**MeO3PyPyBTM+TsOH@PyBTM**	-0.018523	-0.016996	0.001280	-48.6	-44.6	3.4
**3PyPyBTM+BH_3@3Py**	-0.055865	-0.051109	-0.035057	-146.7	-134.2	-92.0
**3PyPyBTM+ BH_3@PyBTM**	-0.051144	-0.046544	-0.030285	-134.3	-122.2	-79.5
**4PyPyBTM+ BH_3@4Py**	-0.057149	-0.052356	-0.036138	-150.0	-137.5	-94.9
**4PyPyBTM+ BH_3@PyBTM**	-0.051036	-0.046455	-0.030246	-134.0	-122.0	-79.4
**MeO3PyPyBTM+ BH_3@MeO3Py**	-0.039303	-0.034747	-0.019121	-103.2	-91.2	-50.2
**MeO3PyPyBTM+ BH_3@PyBTM**	-0.051412	-0.046776	-0.031053	-135.0	-122.8	-81.5

Table S7 TD-DFT-calculated first excited states using UB3LYP/6-31G(d,p) in chloroform

	E_1 /eV	λ_1 /nm	f_1	<S^2>		
**3PyPyBTM**	2.4208	512.16	0.0699	0.938	140B ->141B	0.91149
**3Py$_2$PyBTM**	2.3723	522.62	0.0904	0.937	152B ->153B	0.92478
**4PyPyBTM**	2.4755	500.84	0.0483	0.955	140B ->141B	0.92048
**4Py$_2$PyBTM**	2.4267	510.92	0.0592	0.960	152B ->153B	0.92431
**MeO3PyPyBTM**	2.2227	557.82	0.0990	0.889	148B ->149B	0.92503
**MeO3Py$_2$PyBTM**	2.1980	564.08	0.1375	0.897	168B ->169B	0.92713
**3PyPyBTM+TsOH@3Py**	2.4940	497.14	0.0253	0.969	182B ->186B	0.80331
**3PyPyBTM+TsOH@PyBTM**	2.3322	531.63	0.0877	0.897	185B ->186B	0.92293
**4PyPyBTM+TsOH@4Py**	2.4524	505.57	0.0063	0.930	185B ->186B	0.75227
**4PyPyBTM+TsOH@PyBTM**	2.4067	515.16	0.0651	0.916	184B ->186B	0.92669
**MeO3PyPyBTM+TsOH@MeO3Py**	2.4746	501.03	0.0449	0.974	190B ->194B	0.89479
**MeO3PyPyBTM+TsOH@PyBTM**	2.0978	591.01	0.1107	0.856	193B ->194B	0.94333
**3PyPyBTM+BH$_3$@3Py**	2.4914	497.65	0.0397	0.963	144B ->145B	0.89416
**3PyPyBTM+ BH$_3$@PyBTM**	2.2713	545.86	0.0944	0.877	144B ->145B	0.92960
**4PyPyBTM+ BH$_3$@4Py**	2.4585	504.30	0.0318	1.024	144B ->145B	0.81399
**4PyPyBTM+ BH$_3$@PyBTM**	2.3501	527.57	0.0716	0.893	144B ->145B	0.93673
**MeO3PyPyBTM+ BH$_3$@MeO3Py**	2.4775	500.45	0.0494	0.952	152B ->153B	0.91649
**MeO3PyPyBTM+ BH$_3$@PyBTM**	2.0181	614.38	0.1139	0.842	152B ->153B	0.95130

II. Cartesian coordinates of the optimized geometries by DFT calculation (UB3LYP/6-31G(d,p)) in $CHCl_3$

3PyPyBTM

Standard orientation:

Center Number	Atomic Number	Atomic Type	Coordinates (Angstroms) X	Y	Z
1	6	0	6.809272	-2.171853	0.230690
2	1	0	7.227500	-3.156173	0.412343
3	6	0	5.433688	-1.965060	0.243736
4	6	0	5.869315	0.337419	-0.251343
5	6	0	4.930001	-0.679690	-0.006403
6	6	0	3.478130	-0.394042	-0.005839
7	6	0	2.924185	0.540485	-0.891475
8	6	0	2.614203	-1.049582	0.881900
9	1	0	3.547151	1.036324	-1.625660
10	6	0	1.561578	0.803423	-0.881181
11	6	0	1.253703	-0.775960	0.879193
12	1	0	3.005724	-1.747202	1.612249
13	17	0	0.984182	1.909525	-2.119754
14	6	0	0.652336	0.163833	0.001519
15	17	0	0.306832	-1.586080	2.119732
16	6	0	-0.790849	0.450971	0.005767
17	6	0	-1.263382	1.848051	0.024287
18	6	0	-1.769668	-0.652125	-0.008089
19	6	0	-0.760935	2.824221	0.918846
20	6	0	-2.264146	2.341521	-0.847449
21	6	0	-2.858766	-0.720890	0.898525
22	6	0	-1.695956	-1.727342	-0.930913
23	6	0	-1.234791	4.134632	0.904605
24	17	0	0.417099	2.433411	2.156041
25	6	0	-2.686353	3.668347	-0.791911
26	17	0	-2.970100	1.349492	-2.107919
27	17	0	-3.047138	0.469783	2.173729
28	6	0	-3.791713	-1.751987	0.894564
29	17	0	-0.473044	-1.758180	-2.188954
30	6	0	-2.610820	-2.774517	-0.956030
31	7	0	-2.183702	4.557909	0.066813
32	1	0	-0.832329	4.856824	1.609049
33	1	0	-3.451794	4.015608	-1.479894
34	6	0	-3.655809	-2.774893	-0.038102
35	1	0	-4.594864	-1.763226	1.619864
36	1	0	-2.517489	-3.561935	-1.692603
37	1	0	4.756465	-2.794712	0.421773
38	7	0	7.192573	0.151728	-0.270855
39	6	0	7.646253	-1.087711	-0.035689
40	1	0	8.725841	-1.218901	-0.061662
41	17	0	-4.817499	-4.083547	-0.056666
42	1	0	5.529226	1.357548	-0.417964

3Py$_2$PyBTM

Standard orientation:

Center Number	Atomic Number	Atomic Type	Coordinates (Angstroms) X	Y	Z
1	6	0	-6.377127	-3.958390	-0.276935
2	1	0	-6.462183	-5.021772	-0.473909
3	6	0	-5.138075	-3.325964	-0.284476
4	6	0	-6.279489	-1.287980	0.243793
5	6	0	-5.066835	-1.950982	-0.014678
6	6	0	-3.780416	-1.219532	-0.008274
7	6	0	-3.548920	-0.171315	0.892835
8	6	0	-2.755689	-1.553108	-0.904313
9	1	0	-4.294559	0.089752	1.633669
10	6	0	-2.340119	0.510564	0.889189
11	6	0	-1.552233	-0.861919	-0.894284
12	1	0	-2.907663	-2.327203	-1.646663
13	17	0	-2.140327	1.724000	2.145883
14	6	0	-1.277501	0.206502	-0.001084
15	17	0	-0.400271	-1.311837	-2.144659
16	6	0	-0.000002	0.937791	0.000001
17	6	0	0.000002	2.413044	0.000002
18	6	0	1.277496	0.206499	0.001084
19	6	0	-0.789772	3.189222	-0.882534
20	6	0	0.789783	3.189213	0.882539
21	6	0	2.340111	0.510557	-0.889194
22	6	0	1.552231	-0.861917	0.894290
23	6	0	-0.762896	4.581999	-0.847617
24	17	0	-1.779149	2.458614	-2.131249
25	6	0	0.762920	4.581990	0.847625
26	17	0	1.779158	2.458592	2.131248
27	17	0	2.140314	1.723987	-2.145891
28	6	0	3.548912	-0.171323	-0.892841
29	17	0	0.400275	-1.311827	2.144673
30	6	0	2.755688	-1.553106	0.904318
31	7	0	0.000015	5.275600	0.000005
32	1	0	-1.376771	5.146729	-1.543376
33	1	0	1.376802	5.146713	1.543384
34	6	0	3.780411	-1.219536	0.008272
35	1	0	4.294547	0.089739	-1.633679
36	1	0	2.907664	-2.327197	1.646671
37	1	0	-4.233497	-3.895705	-0.473548
38	1	0	-6.279759	-0.215232	0.426309
39	6	0	5.066830	-1.950984	0.014676
40	6	0	5.138074	-3.325963	0.284488
41	6	0	6.279482	-1.287984	-0.243813
42	6	0	6.377126	-3.958388	0.276944
43	1	0	4.233498	-3.895704	0.473573
44	1	0	6.279750	-0.215238	-0.426341
45	6	0	7.513789	-3.199586	-0.004080
46	1	0	6.462185	-5.021768	0.473929
47	1	0	8.496070	-3.666535	-0.026258
48	7	0	7.475576	-1.883869	-0.258478
49	7	0	-7.475583	-1.883866	0.258455
50	6	0	-7.513792	-3.199585	0.004071
51	1	0	-8.496074	-3.666535	0.026246

4PyPyBTM

Standard orientation:

Center Number	Atomic Number	Atomic Type	Coordinates (Angstroms) X	Y	Z
1	6	0	-3.647296	-2.783971	-0.042323
2	6	0	-3.787101	-1.762464	0.891374
3	6	0	-2.601893	-2.779148	-0.959821
4	1	0	-4.590452	-1.777314	1.616383
5	6	0	-2.857908	-0.728064	0.896781
6	6	0	-1.690612	-1.728948	-0.933279
7	1	0	-2.505538	-3.565577	-1.697056
8	17	0	-3.050872	0.460571	2.173020
9	6	0	-1.768691	-0.654857	-0.009453
10	17	0	-0.466937	-1.754109	-2.190521
11	6	0	-0.793943	0.451568	0.005897
12	6	0	-1.270625	1.847287	0.027233
13	6	0	0.650804	0.169370	0.000587
14	6	0	-2.272848	2.339087	-0.843734
15	6	0	-0.770734	2.823168	0.923416
16	6	0	1.556396	0.812673	-0.883077
17	6	0	1.255193	-0.767540	0.878982
18	6	0	-2.698615	3.664737	-0.785918
19	17	0	-2.976194	1.347086	-2.105391
20	6	0	-1.248296	4.132266	0.911464
21	17	0	0.408421	2.433176	2.159735
22	17	0	0.974062	1.916002	-2.121260
23	6	0	2.920511	0.557683	-0.890639
24	17	0	0.311932	-1.579772	2.119891
25	6	0	2.616691	-1.037758	0.879508
26	7	0	-2.198324	4.554155	0.074288
27	1	0	-3.465072	4.011070	-1.473220
28	1	0	-0.847997	4.854405	1.617187
29	6	0	3.476305	-0.376204	-0.006756
30	1	0	3.543243	1.059377	-1.621092
31	1	0	3.011367	-1.735932	1.607396
32	6	0	4.931761	-0.655544	-0.009215
33	6	0	5.433190	-1.937002	0.263467
34	6	0	5.870162	0.350325	-0.284100
35	6	0	6.810636	-2.145868	0.248398
36	1	0	4.765254	-2.768893	0.459785
37	1	0	5.555938	1.370197	-0.478547
38	1	0	7.208992	-3.137597	0.452504
39	6	0	7.227398	0.035852	-0.273272
40	1	0	7.963094	0.810590	-0.479123
41	7	0	7.710623	-1.187377	-0.013496
42	17	0	-4.804401	-4.096346	-0.062702

$4Py_2PyBTM$

Standard orientation:

Center Number	Atomic Number	Atomic Type	Coordinates (Angstroms) X	Y	Z
1	6	0	-6.368192	-3.953270	-0.299260
2	1	0	-6.427492	-5.016905	-0.520673
3	6	0	-5.130992	-3.312634	-0.309008
4	6	0	-6.277422	-1.296423	0.276404
5	6	0	-5.066637	-1.942623	-0.014241
6	6	0	-3.778151	-1.210316	-0.009635
7	6	0	-3.549277	-0.161579	0.890342
8	6	0	-2.754144	-1.546159	-0.904509
9	1	0	-4.297872	0.101548	1.627487
10	6	0	-2.339462	0.518347	0.890004
11	6	0	-1.551502	-0.853038	-0.895991
12	1	0	-2.905706	-2.321995	-1.644882
13	17	0	-2.139425	1.730757	2.146927
14	6	0	-1.277651	0.214142	-0.001216
15	17	0	-0.400147	-1.300149	-2.146763
16	6	0	0.000000	0.945885	-0.000006
17	6	0	0.000000	2.421265	-0.000000
18	6	0	1.277650	0.214141	0.001209
19	6	0	-0.789503	3.196944	-0.883072
20	6	0	0.789503	3.196939	0.883077
21	6	0	2.339464	0.518347	-0.890008
22	6	0	1.551500	-0.853040	0.895984
23	6	0	-0.762455	4.589782	-0.848167
24	17	0	-1.778236	2.465880	-2.131685
25	6	0	0.762452	4.589778	0.848181
26	17	0	1.778237	2.465869	2.131686
27	17	0	2.139428	1.730756	-2.146931
28	6	0	3.549279	-0.161579	-0.890343
29	17	0	0.400142	-1.300153	2.146753
30	6	0	2.754142	-1.546159	0.904505
31	7	0	-0.000002	5.283100	0.000010
32	1	0	-1.375801	5.154643	-1.544264
33	1	0	1.375797	5.154635	1.544283
34	6	0	3.778151	-1.210316	0.009634
35	1	0	4.297875	0.101549	-1.627486
36	1	0	2.905703	-2.321996	1.644878
37	1	0	-4.232152	-3.882669	-0.518456
38	1	0	-6.306673	-0.232983	0.488463
39	6	0	5.066638	-1.942622	0.014243
40	6	0	5.130992	-3.312634	0.309009
41	6	0	6.277424	-1.296421	-0.276398
42	6	0	6.368193	-3.953268	0.299264
43	1	0	4.232152	-3.882669	0.518454
44	1	0	6.306674	-0.232981	-0.488456
45	1	0	6.427492	-5.016903	0.520677
46	6	0	-7.461656	-2.030165	0.258788
47	1	0	-8.406389	-1.536462	0.476908
48	6	0	7.461658	-2.030163	-0.258779
49	1	0	8.406391	-1.536458	-0.476896
50	7	0	-7.527343	-3.339381	-0.022198
51	7	0	7.527344	-3.339379	0.022206

MeO3PyPyBTM

Standard orientation:

Center Number	Atomic Number	Atomic Type	Coordinates (Angstroms) X	Y	Z
1	6	0	6.173359	-2.922485	0.252182
2	1	0	6.430613	-3.967476	0.381443
3	6	0	4.842362	-2.500817	0.237612
4	6	0	5.622957	-0.268175	-0.101946
5	6	0	4.519849	-1.153140	0.058130
6	6	0	3.107581	-0.707024	0.056253
7	6	0	2.634234	0.286264	-0.813805
8	6	0	2.181756	-1.311789	0.919446
9	1	0	3.309111	0.768790	-1.506467
10	6	0	1.293576	0.640248	-0.819014
11	6	0	0.844462	-0.942480	0.903786
12	1	0	2.510272	-2.048607	1.642157
13	17	0	0.817145	1.810762	-2.044403
14	6	0	0.323653	0.051401	0.035124
15	17	0	-0.173820	-1.703006	2.120932
16	6	0	-1.094552	0.438602	0.021687
17	6	0	-1.472145	1.863911	0.049331
18	6	0	-2.147046	-0.594240	-0.020357
19	6	0	-0.918925	2.794544	0.962429
20	6	0	-2.425148	2.433372	-0.829626
21	6	0	-3.251184	-0.599900	0.870202
22	6	0	-2.135000	-1.658966	-0.957973
23	6	0	-1.303785	4.133828	0.957208
24	17	0	0.212556	2.312483	2.210660
25	6	0	-2.758609	3.784698	-0.764191
26	17	0	-3.178129	1.504871	-2.111620
27	17	0	-3.376431	0.585009	2.158806
28	6	0	-4.252842	-1.564026	0.839305
29	17	0	-0.900672	-1.755989	-2.201728
30	6	0	-3.119236	-2.640274	-1.010233
31	7	0	-2.210072	4.629340	0.111757
32	1	0	-0.863944	4.819535	1.675652
33	1	0	-3.489235	4.189879	-1.458358
34	6	0	-4.174408	-2.581223	-0.106002
35	1	0	-5.065171	-1.529347	1.553550
36	1	0	-3.070157	-3.421832	-1.757247
37	1	0	4.043163	-3.227776	0.343129
38	7	0	6.892230	-0.656204	-0.088634
39	6	0	7.159850	-1.960828	0.083174
40	1	0	8.212396	-2.235027	0.080922
41	8	0	5.348141	1.045027	-0.257720
42	6	0	6.457137	1.947509	-0.383563
43	1	0	7.095320	1.907459	0.502399
44	1	0	7.058511	1.709074	-1.264154
45	1	0	6.009246	2.935894	-0.485198
46	17	0	-5.423458	-3.806293	-0.158698

MeO3Py$_2$PyBTM

Standard orientation:

Center Number	Atomic Number	Atomic Type	X	Y	Z
1	6	0	6.211254	-4.109948	0.355705
2	1	0	6.203011	-5.182680	0.511005
3	6	0	5.024939	-3.374152	0.320165
4	6	0	6.331804	-1.411296	-0.063178
5	6	0	5.044339	-1.993401	0.107633
6	6	0	3.785474	-1.213007	0.084332
7	6	0	3.572963	-0.157072	-0.813980
8	6	0	2.738211	-1.547452	0.955593
9	1	0	4.346381	0.125256	-1.514161
10	6	0	2.361476	0.517422	-0.836656
11	6	0	1.534121	-0.858476	0.921852
12	1	0	2.873241	-2.324442	1.698091
13	17	0	2.189307	1.736662	-2.095234
14	6	0	1.276439	0.211168	0.026240
15	17	0	0.357313	-1.313772	2.149021
16	6	0	-0.000000	0.942118	0.000001
17	6	0	0.000000	2.417352	0.000001
18	6	0	-1.276440	0.211168	-0.026238
19	6	0	0.769383	3.194080	0.899877
20	6	0	-0.769380	3.194083	-0.899875
21	6	0	-2.361478	0.517423	0.836657
22	6	0	-1.534120	-0.858478	-0.921848
23	6	0	0.743539	4.586767	0.864419
24	17	0	1.730260	2.463539	2.170993
25	6	0	-0.743532	4.586770	-0.864415
26	17	0	-1.730258	2.463547	-2.170993
27	17	0	-2.189311	1.736665	2.095234
28	6	0	-3.572965	-0.157071	0.813979
29	17	0	-0.357309	-1.313776	-2.149013
30	6	0	-2.738210	-1.547454	-0.955590
31	7	0	0.000004	5.280923	0.000002
32	1	0	1.341713	5.151250	1.573974
33	1	0	-1.341704	5.151255	-1.573971
34	6	0	-3.785475	-1.213006	-0.084331
35	1	0	-4.346384	0.125259	1.514160
36	1	0	-2.873239	-2.324445	-1.698086
37	1	0	4.071067	-3.879205	0.435590
38	6	0	-5.044340	-1.993401	-0.107633
39	6	0	-5.024939	-3.374152	-0.320162
40	6	0	-6.331805	-1.411296	0.063174
41	6	0	-6.211254	-4.109949	-0.355703
42	1	0	-4.071067	-3.879206	-0.435584
43	6	0	-7.404478	-3.424978	-0.173417
44	1	0	-6.203011	-5.182681	-0.511001
45	1	0	-8.357210	-3.949575	-0.186542
46	7	0	-7.466577	-2.099061	0.030285
47	7	0	7.466576	-2.099061	-0.030291
48	6	0	7.404478	-3.424977	0.173414
49	1	0	8.357209	-3.949575	0.186537
50	8	0	-6.389030	-0.074901	0.251613
51	8	0	6.389027	-0.074902	-0.251622
52	6	0	7.686325	0.523566	-0.387610
53	1	0	8.290375	0.353461	0.506906
54	1	0	8.215698	0.119832	-1.254035
55	1	0	7.495771	1.588412	-0.520547
56	6	0	-7.686329	0.523567	0.387596
57	1	0	-8.290375	0.353459	-0.506921
58	1	0	-8.215703	0.119835	1.254020
59	1	0	-7.495775	1.588414	0.520530

3PyPyBTM+TsOH@3Py

Standard orientation:

Center Number	Atomic Number	Atomic Type	Coordinates (Angstroms) X	Y	Z
1	6	0	-2.940113	-4.838650	-0.852190
2	1	0	-2.909584	-5.875032	-1.164901
3	6	0	-1.776315	-4.075930	-0.774673
4	6	0	-3.079473	-2.211415	-0.032546
5	6	0	-1.825694	-2.734294	-0.359683
6	6	0	-0.610731	-1.893248	-0.266858
7	6	0	-0.482107	-0.922229	0.733699
8	6	0	0.438874	-2.053904	-1.179233
9	1	0	-1.253915	-0.792530	1.482706
10	6	0	0.666579	-0.146270	0.815349
11	6	0	1.578064	-1.265951	-1.087571
12	1	0	0.357694	-2.760834	-1.995959
13	17	0	0.757196	0.948451	2.182889
14	6	0	1.754432	-0.274433	-0.087699
15	17	0	2.766841	-1.488809	-2.360315
16	6	0	2.968728	0.554095	0.005844
17	6	0	2.851687	2.017364	0.150327
18	6	0	4.301759	-0.073829	-0.044147
19	6	0	2.006519	2.810490	-0.662917
20	6	0	3.574712	2.762111	1.113368
21	6	0	5.335012	0.391687	-0.898051
22	6	0	4.651388	-1.189201	0.760190
23	6	0	1.922992	4.191137	-0.492962
24	17	0	1.084462	2.128404	-1.987555
25	6	0	3.437386	4.145393	1.212775
26	17	0	4.613830	1.992814	2.295629
27	17	0	5.046439	1.695933	-2.035215
28	6	0	6.598531	-0.186253	-0.951268
29	17	0	3.530928	-1.837570	1.944695
30	6	0	5.904985	-1.790068	0.725956
31	7	0	2.625583	4.855505	0.426811
32	1	0	1.268766	4.771256	-1.137234
33	1	0	4.001418	4.686335	1.967076
34	6	0	6.871334	-1.278254	-0.133820
35	1	0	7.345609	0.198910	-1.632981
36	1	0	6.125298	-2.628136	1.374280
37	1	0	-0.822064	-4.531287	-1.017366
38	7	0	-4.181821	-2.972007	-0.112545
39	6	0	-4.149893	-4.255787	-0.508023
40	1	0	-5.097939	-4.778496	-0.535004
41	17	0	8.454531	-2.020333	-0.188145
42	1	0	-3.260327	-1.188843	0.284650
43	1	0	-5.140007	-2.507645	0.156838
44	8	0	-6.439386	-1.884935	0.494975
45	8	0	-6.729357	-0.575263	2.595475
46	8	0	-4.956569	0.050747	0.929869
47	6	0	-7.481681	0.525529	0.320569
48	6	0	-7.078092	1.248058	-0.803229
49	6	0	-8.803702	0.592938	0.764305
50	6	0	-8.006232	2.033590	-1.485231
51	1	0	-6.043046	1.208545	-1.124358
52	6	0	-9.721464	1.381069	0.072268
53	1	0	-9.101123	0.047366	1.653187
54	6	0	-9.341615	2.108974	-1.064792
55	1	0	-7.686637	2.603078	-2.354077
56	1	0	-10.748001	1.437795	0.424878
57	6	0	-10.348722	2.937925	-1.825259
58	1	0	-10.865540	2.332174	-2.579989
59	1	0	-11.113909	3.347371	-1.159445
60	1	0	-9.868861	3.769779	-2.348487
61	16	0	-6.307277	-0.527107	1.179569

3PyPyBTM+TsOH@PyBTM

Standard orientation:

Center Number	Atomic Number	Atomic Type	Coordinates (Angstroms) X	Y	Z
1	6	0	8.077987	-3.428464	-1.239845
2	1	0	9.034387	-3.127690	-1.654070
3	6	0	7.045699	-2.509619	-1.081896
4	6	0	5.723905	-4.290875	-0.177462
5	6	0	5.826502	-2.934195	-0.532255
6	6	0	4.690565	-2.006013	-0.339171
7	6	0	3.831746	-2.131089	0.761761
8	6	0	4.440297	-0.973675	-1.253772
9	1	0	4.014234	-2.885305	1.517192
10	6	0	2.768943	-1.255418	0.932224
11	6	0	3.375032	-0.104325	-1.068292
12	1	0	5.051318	-0.869120	-2.141930
13	17	0	1.839559	-1.452794	2.411094
14	6	0	2.482255	-0.196049	0.031797
15	17	0	3.111303	1.069979	-2.349343
16	6	0	1.357709	0.731960	0.225861
17	6	0	0.014501	0.208319	0.532574
18	6	0	1.565916	2.187768	0.115423
19	6	0	-0.586110	-0.850979	-0.193590
20	6	0	-0.796647	0.722118	1.575746
21	6	0	0.734670	3.021371	-0.676222
22	6	0	2.614190	2.862560	0.793244
23	6	0	-1.859172	-1.317498	0.117646
24	17	0	0.184127	-1.580347	-1.581961
25	6	0	-2.064526	0.210021	1.828103
26	17	0	-0.232453	1.963377	2.669101
27	17	0	-0.549225	2.340526	-1.660000
28	6	0	0.917544	4.394920	-0.789353
29	17	0	3.677304	2.007890	1.895825
30	6	0	2.823731	4.233922	0.696399
31	7	0	-2.583543	-0.790854	1.110567
32	1	0	-2.320392	-2.118959	-0.449104
33	1	0	-2.667604	0.610319	2.635812
34	6	0	1.967900	4.989948	-0.098011
35	1	0	0.263875	4.981991	-1.421186
36	1	0	3.628491	4.701971	1.248200
37	1	0	7.190991	-1.469890	-1.358626
38	7	0	6.708737	-5.182680	-0.320311
39	6	0	7.865296	-4.748535	-0.840960
40	1	0	8.655839	-5.488747	-0.943246
41	17	0	2.216121	6.716339	-0.229644
42	1	0	4.787583	-4.675810	0.221454
43	1	0	-4.029482	-1.372549	1.429964
44	8	0	-4.965368	-1.726550	1.708673
45	16	0	-5.646590	-2.569998	0.536976
46	8	0	-4.619468	-2.893924	-0.466715
47	8	0	-6.404110	-3.651444	1.168731
48	6	0	-6.807009	-1.428879	-0.197023
49	6	0	-8.067712	-1.265590	0.382083
50	6	0	-6.435665	-0.699588	-1.327149
51	6	0	-8.958489	-0.355802	-0.180738
52	1	0	-8.346437	-1.849247	1.252305
53	6	0	-7.341805	0.204979	-1.877902
54	1	0	-5.458200	-0.847657	-1.772012
55	1	0	-9.939677	-0.226034	0.267260
56	1	0	-7.057129	0.772324	-2.759487
57	6	0	-8.613007	0.391940	-1.317185
58	6	0	-9.599689	1.350728	-1.936856
59	1	0	-10.280381	0.823011	-2.615913
60	1	0	-10.215362	1.837260	-1.174933
61	1	0	-9.093226	2.125938	-2.517877

4PyPyBTM+TsOH@4Py

Standard orientation:

Center Number	Atomic Number	Atomic Type	Coordinates (Angstroms) X	Y	Z
1	6	0	5.907017	3.402481	0.140995
2	6	0	6.163315	2.473786	1.144626
3	6	0	4.996468	3.128733	-0.874517
4	1	0	6.858103	2.696509	1.943930
5	6	0	5.490598	1.257317	1.120195
6	6	0	4.341122	1.902426	-0.875290
7	1	0	4.811901	3.845463	-1.664089
8	17	0	5.796478	0.190876	2.478768
9	6	0	4.551841	0.908069	0.114933
10	17	0	3.288045	1.605035	-2.246750
11	6	0	3.851607	-0.388912	0.097901
12	6	0	4.618151	-1.639762	0.253898
13	6	0	2.389963	-0.440700	-0.075193
14	6	0	5.796388	-1.923394	-0.478234
15	6	0	4.245685	-2.668738	1.152154
16	6	0	1.758452	-1.302721	-1.009941
17	6	0	1.499900	0.368884	0.678625
18	6	0	6.493612	-3.116296	-0.295096
19	17	0	6.402632	-0.845538	-1.718472
20	6	0	4.997922	-3.835836	1.270492
21	17	0	2.875347	-2.508031	2.232455
22	17	0	2.705779	-2.291155	-2.107164
23	6	0	0.381709	-1.360343	-1.176058
24	17	0	2.091244	1.409310	1.961471
25	6	0	0.121999	0.332713	0.514176
26	7	0	6.106275	-4.062523	0.562557
27	1	0	7.390720	-3.306545	-0.877077
28	1	0	4.689027	-4.603044	1.974529
29	6	0	-0.459367	-0.537133	-0.416870
30	1	0	-0.024862	-2.014057	-1.937715
31	1	0	-0.495903	0.951606	1.152855
32	6	0	-1.927191	-0.584318	-0.594697
33	6	0	-2.715438	0.570486	-0.445947
34	6	0	-2.581151	-1.787554	-0.919357
35	6	0	-4.087237	0.498158	-0.616537
36	1	0	-2.265192	1.530187	-0.224321
37	1	0	-2.030901	-2.714423	-1.022912
38	1	0	-4.766503	1.339583	-0.527322
39	6	0	-3.954498	-1.800789	-1.079060
40	1	0	-4.510777	-2.697781	-1.322406
41	7	0	-4.670994	-0.673699	-0.925158
42	17	0	6.742011	4.938853	0.156889
43	1	0	-5.755535	-0.695427	-1.054074
44	8	0	-7.241808	-0.754035	-1.209675
45	8	0	-6.978604	1.640230	-0.632498
46	8	0	-8.508354	0.957447	-2.504332
47	6	0	-9.258270	0.456263	-0.027363
48	6	0	-9.049953	0.738561	1.323388
49	6	0	-10.507334	0.017490	-0.470386
50	6	0	-10.096254	0.573603	2.229994
51	1	0	-8.082967	1.102836	1.652437
52	6	0	-11.544335	-0.144454	0.446506
53	1	0	-10.665204	-0.175212	-1.525837
54	6	0	-11.356338	0.124631	1.810047
55	1	0	-9.933761	0.803966	3.279757
56	1	0	-12.517784	-0.478449	0.096908
57	6	0	-12.476957	-0.080638	2.800959
58	1	0	-12.493254	-1.115007	3.166307
59	1	0	-12.364863	0.570559	3.672384
60	1	0	-13.452632	0.117833	2.347957
61	16	0	-7.905070	0.619049	-1.197466

4PyPyBTM+TsOH@PyBTM

Standard orientation:

Center Number	Atomic Number	Atomic Type	Coordinates (Angstroms) X	Y	Z
1	6	0	2.258467	4.978656	-0.137408
2	6	0	1.186191	4.435323	-0.837685
3	6	0	3.064554	4.183667	0.670963
4	1	0	0.571104	5.052207	-1.479773
5	6	0	0.930373	3.073945	-0.719706
6	6	0	2.782556	2.825774	0.772538
7	1	0	3.886343	4.612461	1.229559
8	17	0	-0.376383	2.455598	-1.714254
9	6	0	1.708475	2.202643	0.085525
10	17	0	3.785953	1.923104	1.892437
11	6	0	1.424325	0.760495	0.199375
12	6	0	0.051977	0.305844	0.488643
13	6	0	2.502054	-0.226783	0.026157
14	6	0	-0.744339	0.859302	1.522669
15	6	0	-0.590684	-0.723538	-0.243984
16	6	0	2.718859	-1.293850	0.936812
17	6	0	3.411039	-0.190307	-1.063605
18	6	0	-2.036340	0.405479	1.764466
19	17	0	-0.133881	2.076066	2.618387
20	6	0	-1.886309	-1.131385	0.056264
21	17	0	0.157804	-1.486194	-1.626212
22	17	0	1.764237	-1.430050	2.405985
23	6	0	3.730703	-2.230436	0.780876
24	17	0	3.228261	0.989651	-2.352534
25	6	0	4.430461	-1.117309	-1.230409
26	7	0	-2.593526	-0.572174	1.043568
27	1	0	-2.627593	0.833153	2.566860
28	1	0	-2.379006	-1.912180	-0.512798
29	6	0	4.607977	-2.157370	-0.308822
30	1	0	3.859807	-2.993494	1.538450
31	1	0	5.059301	-1.050611	-2.109696
32	6	0	5.692639	-3.152607	-0.480967
33	6	0	6.934046	-2.796662	-1.028639
34	6	0	5.523301	-4.492709	-0.102535
35	6	0	7.920197	-3.771064	-1.167725
36	1	0	7.144771	-1.773080	-1.319480
37	1	0	4.581402	-4.844569	0.304608
38	1	0	8.888650	-3.503850	-1.585449
39	6	0	6.574419	-5.388818	-0.285405
40	1	0	6.450870	-6.431731	-0.001142
41	7	0	7.762321	-5.052760	-0.808015
42	17	0	2.597942	6.688659	-0.275098
43	1	0	-4.054378	-1.100492	1.364538
44	8	0	-5.002280	-1.416548	1.653177
45	16	0	-5.664535	-2.369680	0.557512
46	8	0	-4.665879	-2.644498	-0.489302
47	8	0	-6.289227	-3.489515	1.264352
48	6	0	-6.958245	-1.362367	-0.148011
49	6	0	-8.206967	-1.312146	0.475307
50	6	0	-6.700977	-0.622133	-1.302851
51	6	0	-9.202656	-0.504704	-0.068400
52	1	0	-8.395804	-1.902868	1.364654
53	6	0	-7.710498	0.178432	-1.833453
54	1	0	-5.730149	-0.682610	-1.781566
55	1	0	-10.175145	-0.462697	0.413970
56	1	0	-7.514600	0.753896	-2.733814
57	6	0	-8.973485	0.250665	-1.228490
58	6	0	-10.071215	1.095037	-1.827483
59	1	0	-9.664185	1.931115	-2.402406
60	1	0	-10.691163	0.499819	-2.508958
61	1	0	-10.732408	1.496822	-1.054617

MeO3PyPyBTM+TsOH@MeO3Py

Standard orientation:

Center Number	Atomic Number	Atomic Type	X	Y	Z
1	6	0	-3.255134	-3.326358	-0.386693
2	1	0	-3.435331	-4.393578	-0.383162
3	6	0	-1.956175	-2.807687	-0.301582
4	6	0	-2.843607	-0.580366	-0.388606
5	6	0	-1.714269	-1.435999	-0.298425
6	6	0	-0.330959	-0.904013	-0.228748
7	6	0	0.013791	0.171210	0.599886
8	6	0	0.682398	-1.515493	-0.977204
9	1	0	-0.734103	0.656739	1.211606
10	6	0	1.329915	0.604579	0.675361
11	6	0	1.993319	-1.064448	-0.895314
12	1	0	0.448487	-2.321601	-1.662035
13	17	0	1.649308	1.880921	1.839853
14	6	0	2.388048	0.016754	-0.066431
15	17	0	3.139699	-1.845354	-1.972674
16	6	0	3.779349	0.492452	0.015194
17	6	0	4.073337	1.932994	-0.104555
18	6	0	4.882092	-0.465595	0.215822
19	6	0	3.537269	2.750718	-1.128719
20	6	0	4.920474	2.628959	0.791432
21	6	0	6.049730	-0.467351	-0.590260
22	6	0	4.858909	-1.455541	1.231918
23	6	0	3.840326	4.108711	-1.206521
24	17	0	2.536466	2.098380	-2.410517
25	6	0	5.176684	3.990256	0.638534
26	17	0	5.626442	1.853806	2.195298
27	17	0	6.202806	0.620054	-1.958284
28	6	0	7.100155	-1.360165	-0.409124
29	17	0	3.538065	-1.537946	2.384470
30	6	0	5.891946	-2.364188	1.434359
31	7	0	4.647717	4.725887	-0.341370
32	1	0	3.417492	4.706356	-2.008880
33	1	0	5.826425	4.495417	1.347316
34	6	0	7.008829	-2.305499	0.607169
35	1	0	7.961706	-1.327848	-1.063245
36	1	0	5.831541	-3.088681	2.236064
37	1	0	-1.113643	-3.486234	-0.219004
38	7	0	-4.081776	-1.106662	-0.465906
39	6	0	-4.308105	-2.447128	-0.466352
40	1	0	-5.357116	-2.722424	-0.534775
41	17	0	8.319223	-3.439093	0.848414
42	1	0	-4.989019	-0.510811	-0.537931
43	8	0	-6.361529	0.144034	-0.593214
44	8	0	-7.932722	-0.387338	-2.452557
45	8	0	-7.405527	-2.106267	-0.701452
46	6	0	-8.917613	-0.052386	-0.027121
47	6	0	-9.689219	1.021981	-0.473219
48	6	0	-9.164111	-0.626963	1.220839
49	6	0	-10.705767	1.521911	0.338905
50	1	0	-9.498986	1.450850	-1.450975
51	6	0	-10.184438	-0.117598	2.022493
52	1	0	-8.569498	-1.471716	1.550977
53	6	0	-10.971239	0.962278	1.597040
54	1	0	-11.305345	2.358075	-0.011418
55	1	0	-10.375560	-0.568226	2.992944
56	6	0	-12.096430	1.487521	2.455958
57	1	0	-13.043371	0.992320	2.207886
58	1	0	-11.905230	1.310179	3.518067
59	1	0	-12.244310	2.561264	2.308484
60	16	0	-7.572409	-0.666988	-1.045688
61	8	0	-2.644350	0.728380	-0.408239
62	6	0	-3.749771	1.652792	-0.545182
63	1	0	-4.431887	1.561192	0.301403
64	1	0	-3.285682	2.636386	-0.559119
65	1	0	-4.289161	1.468009	-1.475193

MeO3PyPyBTM+TsOH@MeO3Py

Standard orientation:

Center Number	Atomic Number	Atomic Type	Coordinates (Angstroms) X	Y	Z
1	6	0	8.101307	-2.590244	-1.254080
2	1	0	9.025813	-2.160648	-1.622299
3	6	0	6.966439	-1.802574	-1.052913
4	6	0	5.821809	-3.761012	-0.300536
5	6	0	5.782495	-2.362999	-0.565750
6	6	0	4.576495	-1.527552	-0.366263
7	6	0	3.709495	-1.706360	0.722303
8	6	0	4.288869	-0.488645	-1.264596
9	1	0	3.901042	-2.484138	1.447789
10	6	0	2.616094	-0.872510	0.898091
11	6	0	3.188707	0.334762	-1.075785
12	1	0	4.901996	-0.344029	-2.145551
13	17	0	1.684327	-1.131160	2.368615
14	6	0	2.290748	0.190418	0.013809
15	17	0	2.888503	1.515491	-2.344808
16	6	0	1.125057	1.063173	0.209851
17	6	0	-0.194324	0.476266	0.504749
18	6	0	1.266128	2.528247	0.113564
19	6	0	-0.742081	-0.602774	-0.234365
20	6	0	-1.033747	0.941531	1.548336
21	6	0	0.403453	3.329712	-0.677240
22	6	0	2.276334	3.244657	0.806056
23	6	0	-1.991482	-1.133798	0.068720
24	17	0	0.065398	-1.279930	-1.627919
25	6	0	-2.275804	0.366311	1.792201
26	17	0	-0.534268	2.199195	2.654697
27	17	0	-0.840423	2.598643	-1.676641
28	6	0	0.523195	4.711304	-0.777150
29	17	0	3.369057	2.429939	1.910187
30	6	0	2.423185	4.625080	0.722679
31	7	0	-2.743096	-0.652824	1.064636
32	1	0	-2.411654	-1.952146	-0.505687
33	1	0	-2.899829	0.729380	2.601612
34	6	0	1.539647	5.347804	-0.072314
35	1	0	-0.152196	5.273281	-1.408995
36	1	0	3.201259	5.124785	1.284930
37	1	0	7.005748	-0.736514	-1.253299
38	7	0	6.893765	-4.520628	-0.489464
39	6	0	8.012600	-3.942998	-0.955435
40	1	0	8.868105	-4.601357	-1.088538
41	8	0	4.689235	-4.339878	0.152331
42	6	0	4.711176	-5.756716	0.383983
43	1	0	4.938064	-6.298683	-0.537206
44	1	0	5.454128	-6.018388	1.141184
45	1	0	3.708534	-6.002983	0.732920
46	17	0	1.709296	7.085231	-0.187352
47	1	0	-4.146032	-1.312957	1.382645
48	8	0	-5.061778	-1.718266	1.667537
49	16	0	-5.664449	-2.667334	0.535421
50	8	0	-4.633161	-2.884890	-0.492861
51	8	0	-6.265483	-3.821869	1.205773
52	6	0	-6.976789	-1.692962	-0.182999
53	6	0	-8.239213	-1.694078	0.414110
54	6	0	-6.720884	-0.926327	-1.320909
55	6	0	-9.250105	-0.911832	-0.138505
56	1	0	-8.426689	-2.304653	1.290270
57	6	0	-7.745407	-0.151452	-1.860689
58	1	0	-5.738802	-0.946904	-1.779628
59	1	0	-10.233293	-0.909949	0.323544
60	1	0	-7.550221	0.444503	-2.747805
61	6	0	-9.022475	-0.130927	-1.281763
62	6	0	-10.134890	0.685978	-1.891695
63	1	0	-10.703432	0.089106	-2.615275
64	1	0	-10.839750	1.031871	-1.130578
65	1	0	-9.745128	1.558296	-2.423274

3PyPyBTM+BH_3@3Py

Standard orientation:

Center Number	Atomic Number	Atomic Type	Coordinates (Angstroms) X	Y	Z
1	6	0	6.481685	-2.471524	0.297901
2	1	0	6.858161	-3.472916	0.469532
3	6	0	5.115447	-2.207309	0.293277
4	6	0	5.633002	0.088119	-0.148812
5	6	0	4.665530	-0.899056	0.062814
6	6	0	3.225158	-0.553739	0.048799
7	6	0	2.721951	0.403989	-0.840272
8	6	0	2.330554	-1.180971	0.924631
9	1	0	3.371735	0.879075	-1.565077
10	6	0	1.369467	0.717493	-0.846313
11	6	0	0.981393	-0.853702	0.908513
12	1	0	2.686312	-1.895703	1.656851
13	17	0	0.849479	1.846790	-2.086102
14	6	0	0.428431	0.109908	0.025244
15	17	0	-0.008908	-1.627823	2.135269
16	6	0	-1.004075	0.451487	0.013372
17	6	0	-1.423039	1.865606	0.033913
18	6	0	-2.022347	-0.614423	-0.019569
19	6	0	-0.893539	2.817388	0.938817
20	6	0	-2.393643	2.400292	-0.847576
21	6	0	-3.125769	-0.646230	0.871894
22	6	0	-1.975556	-1.687269	-0.946914
23	6	0	-1.315895	4.145358	0.924892
24	17	0	0.253520	2.375285	2.187456
25	6	0	-2.764445	3.742450	-0.790806
26	17	0	-3.122619	1.441439	-2.120039
27	17	0	-3.287203	0.544414	2.150213
28	6	0	-4.096033	-1.641956	0.849429
29	17	0	-0.737180	-1.757075	-2.188167
30	6	0	-2.928450	-2.699151	-0.990391
31	7	0	-2.237633	4.607993	0.077796
32	1	0	-0.893927	4.848730	1.636879
33	1	0	-3.507622	4.122190	-1.485963
34	6	0	-3.985517	-2.664765	-0.086840
35	1	0	-4.909183	-1.626705	1.563421
36	1	0	-2.854618	-3.485643	-1.730157
37	1	0	4.403878	-3.011205	0.447794
38	7	0	6.952015	-0.172881	-0.151143
39	6	0	7.374289	-1.433949	0.071866
40	1	0	8.447127	-1.578002	0.064260
41	17	0	-5.194735	-3.928346	-0.128359
42	1	0	5.368765	1.126669	-0.302661
43	5	0	8.020311	0.996950	-0.457754
44	1	0	7.482084	2.043623	-0.169282
45	1	0	8.996669	0.760075	0.220780
46	1	0	8.266768	0.921362	-1.645996

3PyPyBTM+BH_3@PyBTM

Standard orientation:

Center Number	Atomic Number	Atomic Type	Coordinates (Angstroms) X	Y	Z
1	6	0	7.048942	-1.764355	0.226738
2	1	0	7.545122	-2.712204	0.405833
3	6	0	5.661337	-1.668804	0.242234
4	6	0	5.909783	0.662596	-0.249571
5	6	0	5.055951	-0.427375	-0.004975
6	6	0	3.585990	-0.259869	-0.001668
7	6	0	2.956583	0.626784	-0.886626
8	6	0	2.780163	-0.983361	0.888444
9	1	0	3.535843	1.171756	-1.621676
10	6	0	1.577406	0.777324	-0.874221
11	6	0	1.401822	-0.824634	0.886974
12	1	0	3.229115	-1.644383	1.619652
13	17	0	0.907937	1.835450	-2.107902
14	6	0	0.725556	0.062068	0.008120
15	17	0	0.526064	-1.706686	2.129002
16	6	0	-0.736722	0.221199	0.003762
17	6	0	-1.328713	1.566969	0.015573
18	6	0	-1.614951	-0.963304	-0.012609
19	6	0	-0.905221	2.592907	0.899808
20	6	0	-2.381415	1.961933	-0.849458
21	6	0	-2.694953	-1.124064	0.893226
22	6	0	-1.441575	-2.030390	-0.931611
23	6	0	-1.487407	3.851208	0.894110
24	17	0	0.303841	2.322354	2.127383
25	6	0	-2.924460	3.237170	-0.808218
26	17	0	-3.011748	0.909460	-2.089238
27	17	0	-2.993020	0.054824	2.157982
28	6	0	-3.529425	-2.235876	0.894380
29	17	0	-0.221269	-1.952960	-2.188713
30	6	0	-2.258415	-3.155514	-0.951914
31	7	0	-2.481298	4.171854	0.048753
32	1	0	-1.173691	4.622801	1.584079
33	1	0	-3.721019	3.534681	-1.476628
34	6	0	-3.299430	-3.246791	-0.033420
35	1	0	-4.329495	-2.316805	1.618628
36	1	0	-2.094311	-3.934490	-1.685056
37	1	0	5.053590	-2.550779	0.419416
38	7	0	7.243503	0.583612	-0.270732
39	6	0	7.795569	-0.615798	-0.038599
40	1	0	8.882112	-0.659738	-0.066365
41	17	0	-4.335971	-4.654796	-0.045496
42	1	0	5.489028	1.652590	-0.413655
43	1	0	-2.409713	6.290558	-0.755830
44	1	0	-4.239650	5.557632	-0.351212
45	1	0	-2.995768	6.088991	1.158431
46	5	0	-3.094856	5.664204	0.028805

4PyPyBTM+BH_3@4Py

Standard orientation:

Center Number	Atomic Number	Atomic Type	Coordinates (Angstroms) X	Y	Z
1	6	0	6.623054	-1.827504	0.256749
2	1	0	7.087690	-2.783990	0.459753
3	6	0	5.247968	-1.661299	0.273405
4	6	0	5.585198	0.635570	-0.304880
5	6	0	4.689885	-0.405446	-0.012293
6	6	0	3.226206	-0.189432	-0.008726
7	6	0	2.631527	0.720803	-0.891729
8	6	0	2.399490	-0.890349	0.878330
9	1	0	3.229306	1.249764	-1.623787
10	6	0	1.257104	0.913801	-0.884050
11	6	0	1.027537	-0.679668	0.880051
12	1	0	2.824351	-1.569273	1.607337
13	17	0	0.626122	1.989525	-2.120019
14	6	0	0.382848	0.229583	0.000835
15	17	0	0.123499	-1.532307	2.120270
16	6	0	-1.073678	0.445999	0.006671
17	6	0	-1.612383	1.819263	0.028881
18	6	0	-1.996966	-0.703211	-0.009243
19	6	0	-1.157831	2.814646	0.927359
20	6	0	-2.633968	2.266120	-0.843584
21	6	0	-3.083762	-0.823755	0.895138
22	6	0	-1.869060	-1.773161	-0.932462
23	6	0	-1.692526	4.101497	0.915731
24	17	0	0.034027	2.473923	2.165827
25	6	0	-3.117453	3.571967	-0.785429
26	17	0	-3.291438	1.244570	-2.105719
27	17	0	-3.331525	0.355101	2.170403
28	6	0	-3.965888	-1.898416	0.887961
29	17	0	-0.641814	-1.744728	-2.186204
30	6	0	-2.733272	-2.862198	-0.960861
31	7	0	-2.658179	4.481479	0.076495
32	1	0	-1.326210	4.839945	1.622997
33	1	0	-3.897107	3.885037	-1.473786
34	6	0	-3.779451	-2.912843	-0.045444
35	1	0	-4.769251	-1.948800	1.611334
36	1	0	-2.601121	-3.643653	-1.697809
37	1	0	4.621323	-2.520702	0.479951
38	17	0	-4.877551	-4.274179	-0.067951
39	1	0	5.231385	1.638089	-0.513693
40	1	0	7.668514	1.175110	-0.522125
41	6	0	6.949171	0.396242	-0.302948
42	7	0	7.469303	-0.815990	-0.023100
43	5	0	9.062816	-1.034995	0.024783
44	1	0	9.543674	-0.275096	-0.788726
45	1	0	9.408399	-0.759538	1.158378
46	1	0	9.262178	-2.201988	-0.236619

$4PyPyBTM+BH_3@PyBTM$

Standard orientation:

Center Number	Atomic Number	Atomic Type	Coordinates (Angstroms) X	Y	Z
1	6	0	-3.284404	-3.259288	-0.037107
2	6	0	-3.520796	-2.249693	0.890611
3	6	0	-2.242864	-3.162364	-0.954480
4	1	0	-4.321198	-2.335099	1.613973
5	6	0	-2.692428	-1.133400	0.890611
6	6	0	-1.431991	-2.033023	-0.933119
7	1	0	-2.073788	-3.940371	-1.687519
8	17	0	-2.998197	0.043573	2.155134
9	6	0	-1.612162	-0.967002	-0.014070
10	17	0	-0.210990	-1.948603	-2.188891
11	6	0	-0.740067	0.221663	0.003942
12	6	0	-1.337579	1.565116	0.017688
13	6	0	0.723807	0.069334	0.007978
14	6	0	-2.391258	1.956745	-0.847614
15	6	0	-0.917985	2.591589	0.902958
16	6	0	1.571063	0.787346	-0.876241
17	6	0	1.403292	-0.812001	0.889261
18	6	0	-2.938445	3.230271	-0.805784
19	17	0	-3.017595	0.902866	-2.087955
20	6	0	-1.504339	3.847988	0.897801
21	17	0	0.291430	2.323951	2.130753
22	17	0	0.896111	1.840251	-2.110848
23	6	0	2.951305	0.645826	-0.885641
24	17	0	0.531659	-1.694300	2.133030
25	6	0	2.782528	-0.966420	0.888773
26	7	0	-2.498607	4.165836	0.051902
27	1	0	-3.735696	3.525615	-1.474335
28	1	0	-1.193634	4.620187	1.588454
29	6	0	3.582823	-0.238387	-0.001228
30	1	0	3.529688	1.196199	-1.617416
31	1	0	3.235344	-1.626433	1.618304
32	6	0	5.056143	-0.399213	-0.006798
33	6	0	5.658459	-1.637850	0.259109
34	6	0	5.909726	0.680230	-0.278382
35	6	0	7.048238	-1.735479	0.240959
36	1	0	5.059816	-2.521702	0.452030
37	1	0	5.514440	1.672502	-0.467600
38	1	0	7.525206	-2.692974	0.439562
39	6	0	7.287936	0.475504	-0.270444
40	1	0	7.958973	1.307540	-0.473249
41	7	0	7.867595	-0.706411	-0.017230
42	17	0	-4.313313	-4.672559	-0.050479
43	1	0	-2.432563	6.284322	-0.752859
44	1	0	-4.260661	5.546870	-0.348300
45	1	0	-3.018171	6.081642	1.161431
46	5	0	-3.116294	5.656547	0.031894

MeO3PyPyBTM+BH_3@MeO3Py

Standard orientation:

Center Number	Atomic Number	Atomic Type	Coordinates (Angstroms) X	Y	Z
1	6	0	5.958159	-2.960165	0.136980
2	1	0	6.218369	-4.006144	0.243170
3	6	0	4.631569	-2.538588	0.129166
4	6	0	5.404384	-0.277919	-0.153708
5	6	0	4.326755	-1.181911	-0.017532
6	6	0	2.913646	-0.725744	-0.008984
7	6	0	2.432343	0.229927	-0.913412
8	6	0	2.008441	-1.294164	0.896429
9	1	0	3.095257	0.678148	-1.640293
10	6	0	1.091729	0.588387	-0.907770
11	6	0	0.671697	-0.918113	0.893112
12	1	0	2.348780	-2.005885	1.638930
13	17	0	0.593796	1.706866	-2.168672
14	6	0	0.140382	0.039503	-0.007963
15	17	0	-0.325147	-1.623083	2.157317
16	6	0	-1.278657	0.432119	-0.009433
17	6	0	-1.648457	1.859813	-0.024540
18	6	0	-2.334425	-0.597376	0.002939
19	6	0	-1.074098	2.818348	0.845073
20	6	0	-2.613060	2.402554	-0.907695
21	6	0	-3.424229	-0.565085	0.910822
22	6	0	-2.340261	-1.696483	-0.894325
23	6	0	-1.451319	4.158996	0.798937
24	17	0	0.075466	2.372555	2.090650
25	6	0	-2.937425	3.757670	-0.884541
26	17	0	-3.392166	1.433562	-2.142272
27	17	0	-3.524170	0.666386	2.156741
28	6	0	-4.429373	-1.525701	0.931038
29	17	0	-1.125399	-1.844569	-2.151833
30	6	0	-3.328858	-2.674571	-0.894956
31	7	0	-2.369161	4.629004	-0.048420
32	1	0	-0.995574	4.867274	1.484782
33	1	0	-3.677336	4.142805	-1.580234
34	6	0	-4.369676	-2.577461	0.022704
35	1	0	-5.230190	-1.461549	1.656181
36	1	0	-3.294568	-3.483344	-1.613260
37	1	0	3.827983	-3.261571	0.220450
38	7	0	6.692377	-0.697162	-0.168131
39	6	0	6.955474	-2.016512	-0.020382
40	1	0	8.004297	-2.279427	-0.048938
41	8	0	5.147090	1.030143	-0.332457
42	6	0	5.370422	1.921630	0.787287
43	1	0	4.750163	1.616495	1.635297
44	1	0	6.425040	1.933647	1.062733
45	1	0	5.060550	2.905313	0.437194
46	17	0	-5.623491	-3.797740	0.034730
47	1	0	7.618947	1.149559	-1.185467
48	1	0	8.209398	0.790139	0.703106
49	1	0	8.854356	-0.391906	-0.779971
50	5	0	7.950645	0.309973	-0.383377

MeO3PyPyBTM+BH_3@PyBTM

Standard orientation:

Center Number	Atomic Number	Atomic Type	Coordinates (Angstroms) X	Y	Z
1	6	0	6.415191	-2.696661	0.270438
2	1	0	6.735825	-3.723011	0.406716
3	6	0	5.061430	-2.356695	0.256363
4	6	0	5.704394	-0.082915	-0.102738
5	6	0	4.657315	-1.032214	0.066832
6	6	0	3.221053	-0.673232	0.063124
7	6	0	2.689849	0.290600	-0.807523
8	6	0	2.332226	-1.333234	0.925671
9	1	0	3.335406	0.813135	-1.498992
10	6	0	1.330830	0.563037	-0.814135
11	6	0	0.975167	-1.046908	0.908568
12	1	0	2.704015	-2.048038	1.649422
13	17	0	0.784067	1.704055	-2.037427
14	6	0	0.397770	-0.085489	0.038470
15	17	0	0.003111	-1.864087	2.125089
16	6	0	-1.040933	0.211012	0.016011
17	6	0	-1.505474	1.605411	0.035928
18	6	0	-2.026343	-0.885938	-0.026728
19	6	0	-1.000132	2.578357	0.937006
20	6	0	-2.506849	2.106217	-0.835780
21	6	0	-3.128031	-0.957154	0.863929
22	6	0	-1.941685	-1.952348	-0.958665
23	6	0	-1.464199	3.885031	0.940443
24	17	0	0.164848	2.185251	2.174119
25	6	0	-2.930864	3.425043	-0.784815
26	17	0	-3.215531	1.130630	-2.096416
27	17	0	-3.330886	0.228409	2.141580
28	6	0	-4.062896	-1.985814	0.839782
29	17	0	-0.703569	-1.972844	-2.200581
30	6	0	-2.859447	-2.996133	-1.004250
31	7	0	-2.414147	4.305587	0.088613
32	1	0	-1.088923	4.616581	1.643101
33	1	0	-3.688214	3.801970	-1.458800
34	6	0	-3.916187	-3.001640	-0.099405
35	1	0	-4.876158	-2.000852	1.553630
36	1	0	-2.759108	-3.777647	-1.746174
37	1	0	4.308531	-3.130351	0.369108
38	7	0	6.994712	-0.393633	-0.089561
39	6	0	7.341221	-1.678024	0.091248
40	1	0	8.408406	-1.887978	0.087885
41	17	0	-5.079435	-4.306774	-0.143730
42	8	0	5.351240	1.209803	-0.267258
43	6	0	6.404697	2.176056	-0.403329
44	1	0	7.041800	2.185489	0.484219
45	1	0	7.021142	1.962184	-1.279626
46	1	0	5.898250	3.134175	-0.518303
47	1	0	-2.170901	6.407111	-0.727921
48	1	0	-4.048304	5.845368	-0.273073
49	1	0	-2.723739	6.264638	1.202297
50	5	0	-2.888958	5.847615	0.077550

III. Results of TD-DFT calculations (UB3LYP/6-31G(d,p)) in $CHCl_3$

3PyPyBTM

```
Excitation energies and oscillator strengths:

 Excited State   1:  2.180-A     2.4208 eV  512.16 nm  f=0.0699  <S**2>=0.938
    141A ->142A      -0.20394
    141A ->143A       0.10652
    133B ->141B      -0.12997
    134B ->141B       0.10652
    140B ->141B       0.91149
 This state for optimization and/or second-order correction.
 Total Energy, E(TD-HF/TD-DFT) =  -4213.21904424
 Copying the excited state density for this state as the 1-particle RhoCI density.

 Excited State   2:  2.126-A     2.6201 eV  473.20 nm  f=0.0042  <S**2>=0.880
    141A ->142A      -0.10179
    141A ->143A      -0.10054
    135B ->141B       0.24625
    137B ->141B       0.10651
    138B ->141B      -0.11309
    139B ->141B       0.90980

 Excited State   3:  2.134-A     2.7036 eV  458.59 nm  f=0.0127  <S**2>=0.888
    141A ->142A       0.10374
    138B ->141B       0.93606
    139B ->141B       0.20062

 Excited State   4:  2.171-A     2.7555 eV  449.96 nm  f=0.0208  <S**2>=0.929
    141A ->143A      -0.21121
    135B ->141B       0.59645
    136B ->141B      -0.46706
    137B ->141B       0.38634
    138B ->141B       0.22642
    139B ->141B      -0.30033

 Excited State   5:  2.107-A     2.8217 eV  439.40 nm  f=0.0063  <S**2>=0.860
    141A ->142A       0.11610
    135B ->141B      -0.32715
    136B ->141B       0.19131
    137B ->141B       0.88934

 Excited State   6:  2.333-A     3.0989 eV  400.09 nm  f=0.1254  <S**2>=1.111
    135A ->143A       0.10458
    141A ->142A       0.64497
    141A ->143A       0.11418
    141A ->148A       0.12710
    131B ->141B      -0.10688
    132B ->141B      -0.35425
    133B ->141B       0.27144
    134B ->141B      -0.20334
    135B ->141B       0.13770
    136B ->141B      -0.19525
    140B ->141B       0.27416

 Excited State   7:  2.468-A     3.1256 eV  396.68 nm  f=0.0715  <S**2>=1.273
    140A ->142A       0.17159
    141A ->142A       0.34753
    141A ->143A      -0.32981
    132B ->141B       0.24203
    134B ->141B       0.68263
    134B ->142B      -0.11491
    135B ->141B      -0.11726
    140B ->141B       0.11978
    140B ->142B      -0.21024

 Excited State   8:  2.430-A     3.1953 eV  388.01 nm  f=0.0947  <S**2>=1.226
    140A ->142A      -0.14447
    141A ->142A      -0.30194
    141A ->143A       0.17617
    132B ->141B      -0.31810
    133B ->141B       0.60226
    133B ->142B      -0.11251
    134B ->141B       0.43550
    140B ->141B      -0.13172
```

```
   140B ->142B       0.15200

Excited State   9:  2.099-A     3.2612 eV  380.18 nm  f=0.0057  <S**2>=0.851
   141A ->142A       0.10544
   135B ->141B       0.55843
   136B ->141B       0.80357

Excited State  10:  2.385-A     3.3345 eV  371.82 nm  f=0.1662  <S**2>=1.172
   137A ->144A      -0.15814
   137A ->145A       0.11076
   140A ->143A      -0.13973
   141A ->142A       0.17568
   141A ->143A       0.73491
   132B ->141B       0.20243
   133B ->141B      -0.17530
   134B ->141B       0.30098
   135B ->141B       0.24392
   135B ->143B      -0.12187
   136B ->141B      -0.10406
   137B ->144B       0.13130

Excited State  11:  2.423-A     3.5148 eV  352.75 nm  f=0.0006  <S**2>=1.218
   135A ->143A      -0.10848
   140A ->148A       0.10422
   141A ->152A      -0.10433
   131B ->141B       0.37574
   132B ->141B       0.43066
   133B ->141B       0.56889
   134B ->141B      -0.28696
   135B ->141B       0.10839
   139B ->147B       0.10767
   140B ->141B       0.16752

Excited State  12:  2.987-A     3.6232 eV  342.20 nm  f=0.0596  <S**2>=1.980
   135A ->142A       0.10747
   137A ->144A       0.15686
   139A ->147A      -0.10993
   140A ->142A      -0.34969
   140A ->143A       0.17334
   141A ->142A       0.36904
   141A ->148A       0.14205
   131B ->141B       0.14077
   132B ->141B       0.31143
   133B ->141B      -0.14857
   135B ->141B      -0.11292
   137B ->144B      -0.14085
   139B ->147B      -0.13338
   140B ->142B       0.40652
   140B ->143B      -0.15476

Excited State  13:  2.263-A     3.7391 eV  331.59 nm  f=0.0108  <S**2>=1.030
   141A ->144A       0.94439

Excited State  14:  3.082-A     3.8317 eV  323.58 nm  f=0.0320  <S**2>=2.125
   135A ->143A       0.18371
   137A ->144A       0.31328
   137A ->145A      -0.20811
   138A ->144A       0.11123
   138A ->145A       0.12662
   138A ->146A      -0.17280
   140A ->142A       0.17371
   141A ->142A      -0.21233
   141A ->143A       0.29434
   132B ->141B       0.33832
   135B ->143B       0.16625
   137B ->144B      -0.29752
   137B ->145B      -0.19145
   137B ->146B      -0.11209
   138B ->144B       0.10384
   138B ->146B      -0.17888
   140B ->142B      -0.15921

Excited State  15:  3.204-A     3.8563 eV  321.51 nm  f=0.0090  <S**2>=2.316
   137A ->144A       0.19756
   137A ->145A      -0.14303
   138A ->144A      -0.24611
   138A ->145A      -0.25038
```

138A	->146A	0.29826
138A	->147A	0.10275
140A	->143A	0.11926
141A	->143A	0.31266
141A	->145A	-0.18872
141A	->146A	-0.10242
132B	->141B	-0.22298
137B	->144B	-0.20896
137B	->145B	-0.12818
138B	->144B	-0.25208
138B	->145B	0.18163
138B	->146B	0.32382
138B	->147B	-0.14887

$3Py_2PyBTM$

```
Excitation energies and oscillator strengths:

 Excited State   1:  2.179-A     2.3723 eV  522.62 nm  f=0.0904  <S**2>=0.937
    153A ->154A       0.22465
    143B ->153B       0.12897
    145B ->153B      -0.12666
    151B ->154B       0.10042
    152B ->153B       0.92478
 This state for optimization and/or second-order correction.
 Total Energy, E(TD-HF/TD-DFT) =  -4000.72980106
 Copying the excited state density for this state as the 1-particle RhoCI density.

 Excited State   2:  2.198-A     2.5432 eV  487.51 nm  f=0.0182  <S**2>=0.957
    152A ->154A       0.12864
    153A ->155A       0.24468
    144B ->153B       0.22280
    150B ->153B       0.15397
    151B ->153B       0.88477
    152B ->154B       0.10687

 Excited State   3:  2.094-A     2.6956 eV  459.95 nm  f=0.0093  <S**2>=0.846
    149B ->153B       0.97884

 Excited State   4:  2.127-A     2.6988 eV  459.41 nm  f=0.0081  <S**2>=0.881
    153A ->155A      -0.15260
    144B ->153B      -0.10781
    150B ->153B       0.95728

 Excited State   5:  2.135-A     2.7233 eV  455.27 nm  f=0.0121  <S**2>=0.889
    148B ->153B       0.97121
    148B ->155B      -0.10663

 Excited State   6:  2.509-A     3.0586 eV  405.36 nm  f=0.3316  <S**2>=1.324
    152A ->155A       0.16607
    153A ->154A       0.80546
    145B ->153B       0.21670
    151B ->154B       0.16461
    152B ->153B      -0.28343
    152B ->155B       0.16248

 Excited State   7:  2.295-A     3.0651 eV  404.51 nm  f=0.0574  <S**2>=1.067
    148A ->155A       0.10509
    149A ->158A      -0.10503
    153A ->155A       0.60691
    153A ->157A       0.13167
    153A ->161A       0.12486
    140B ->153B      -0.13649
    142B ->153B      -0.30441
    144B ->153B       0.47305
    148B ->156B      -0.10104
    150B ->153B       0.11331
    151B ->153B      -0.31892

 Excited State   8:  2.232-A     3.1750 eV  390.51 nm  f=0.0626  <S**2>=0.996
    153A ->154A      -0.20898
    143B ->153B       0.37990
    143B ->155B      -0.13047
    145B ->153B       0.83665
    145B ->155B      -0.17349
    152B ->153B       0.13483

 Excited State   9:  2.627-A     3.1861 eV  389.14 nm  f=0.1238  <S**2>=1.476
    152A ->154A       0.25846
    153A ->155A       0.53319
    142B ->153B       0.28542
    144B ->153B      -0.50513
    146B ->153B      -0.10794
    150B ->153B       0.10166
    151B ->153B      -0.18182
    151B ->155B       0.15328
    152B ->154B       0.25364

 Excited State  10:  2.094-A     3.2878 eV  377.10 nm  f=0.0011  <S**2>=0.846
    147B ->153B       0.98335
```

```
Excited State  11:  2.096-A     3.2905 eV  376.79 nm  f=0.0072  <S**2>=0.848
   153A ->155A        0.13089
   146B ->153B        0.97846

Excited State  12:  2.397-A     3.4705 eV  357.25 nm  f=0.0081  <S**2>=1.186
   148A ->154A        0.11478
   141B ->153B       -0.27424
   143B ->153B        0.76899
   145B ->153B       -0.34291
   151B ->154B       -0.11948
   152B ->153B       -0.15529

Excited State  13:  2.782-A     3.5278 eV  351.45 nm  f=0.0294  <S**2>=1.684
   148A ->155A       -0.15804
   150A ->156A       -0.15112
   151A ->160A       -0.13958
   152A ->154A        0.30271
   153A ->155A       -0.35806
   153A ->157A       -0.13042
   153A ->161A       -0.10240
   140B ->153B        0.11602
   144B ->153B        0.52069
   149B ->156B       -0.14633
   149B ->159B        0.11294
   150B ->160B       -0.15360
   151B ->153B       -0.20981
   151B ->155B        0.17973
   152B ->154B        0.31165

Excited State  14:  2.646-A     3.6025 eV  344.17 nm  f=0.0127  <S**2>=1.501
   144A ->155A       -0.10426
   148A ->161A       -0.11620
   152A ->154A       -0.20872
   153A ->155A        0.18179
   153A ->166A       -0.10396
   140B ->153B        0.41592
   142B ->153B        0.57239
   144B ->153B        0.27556
   151B ->155B       -0.13704
   151B ->161B        0.13724
   152B ->154B       -0.23654

Excited State  15:  3.010-A     3.7008 eV  335.02 nm  f=0.0733  <S**2>=2.015
   146A ->154A       -0.10767
   148A ->154A        0.22111
   150A ->160A       -0.14549
   151A ->154A       -0.15732
   151A ->156A       -0.13079
   152A ->155A       -0.30060
   152A ->161A        0.11168
   153A ->154A        0.43310
   153A ->156A        0.10483
   141B ->157B        0.10010
   143B ->153B       -0.18313
   145B ->153B        0.14964
   149B ->160B       -0.16395
   150B ->154B        0.10613
   150B ->156B       -0.15811
   150B ->159B        0.11464
   151B ->154B       -0.32312
   152B ->153B        0.11411
   152B ->155B       -0.29157
   152B ->161B        0.14188
```

4PyPyBTM

```
Excitation energies and oscillator strengths:

 Excited State   1:  2.195-A     2.4755 eV  500.84 nm  f=0.0483  <S**2>=0.955
    141A ->142A       0.22773
    141A ->143A      -0.10119
    131B ->141B      -0.10066
    140B ->141B       0.92048
 This state for optimization and/or second-order correction.
 Total Energy, E(TD-HF/TD-DFT) =  -4213.21708453
 Copying the excited state density for this state as the 1-particle RhoCI density.

 Excited State   2:  2.136-A     2.6202 eV  473.19 nm  f=0.0037  <S**2>=0.890
    141A ->142A      -0.12898
    135B ->141B      -0.25250
    136B ->141B       0.17685
    137B ->141B       0.19323
    138B ->141B       0.18060
    139B ->141B       0.86270
    140B ->141B       0.11796

 Excited State   3:  2.143-A     2.6987 eV  459.43 nm  f=0.0129  <S**2>=0.898
    141A ->142A      -0.13661
    135B ->141B      -0.14383
    138B ->141B       0.90556
    139B ->141B      -0.27893
    140B ->141B       0.10592

 Excited State   4:  2.166-A     2.7453 eV  451.63 nm  f=0.0215  <S**2>=0.923
    141A ->143A       0.20347
    134B ->141B       0.16191
    135B ->141B       0.60005
    136B ->141B       0.10299
    137B ->141B      -0.58202
    138B ->141B       0.21376
    139B ->141B       0.29847
    140B ->141B       0.10973

 Excited State   5:  2.128-A     2.8079 eV  441.55 nm  f=0.0064  <S**2>=0.882
    141A ->142A       0.17507
    135B ->141B       0.51687
    136B ->141B      -0.35319
    137B ->141B       0.68862
    138B ->141B       0.15376
    139B ->141B       0.10972

 Excited State   6:  2.402-A     3.1102 eV  398.64 nm  f=0.1494  <S**2>=1.193
    140A ->142A      -0.12306
    141A ->142A       0.60310
    141A ->143A      -0.17792
    133B ->141B       0.58727
    138B ->141B       0.14194
    140B ->141B      -0.21190
    140B ->142B       0.17010

 Excited State   7:  2.331-A     3.1389 eV  394.99 nm  f=0.0478  <S**2>=1.109
    134A ->143A       0.13743
    138A ->146A       0.10179
    141A ->142A       0.37066
    141A ->143A       0.30889
    141A ->147A      -0.16712
    131B ->141B       0.18613
    132B ->141B       0.60477
    133B ->141B      -0.26440
    135B ->141B      -0.25290

 Excited State   8:  2.344-A     3.1854 eV  389.23 nm  f=0.1231  <S**2>=1.123
    141A ->142A      -0.40020
    132B ->141B       0.42841
    133B ->141B       0.67137
    133B ->142B      -0.12356
    133B ->143B      -0.11448
    136B ->141B      -0.14482
    140B ->141B       0.13555

 Excited State   9:  2.204-A     3.2061 eV  386.72 nm  f=0.0022  <S**2>=0.965
```

```
   135B ->141B       0.20272
   136B ->141B       0.86535
   136B ->142B       0.16801
   137B ->141B       0.31420
   139B ->141B      -0.18084

Excited State  10:  2.170-A     3.3143 eV  374.09 nm  f=0.0021  <S**2>=0.927
   141A ->143A       0.15486
   134B ->141B       0.92720
   134B ->142B       0.12596
   135B ->141B      -0.25792

Excited State  11:  2.437-A     3.3470 eV  370.43 nm  f=0.1599  <S**2>=1.235
   136A ->144A       0.11070
   137A ->144A      -0.11759
   140A ->143A       0.12382
   141A ->143A       0.72907
   141A ->147A       0.11998
   132B ->141B      -0.30552
   133B ->141B       0.18462
   134B ->141B      -0.24174
   135B ->141B      -0.21748
   135B ->143B       0.12789
   137B ->144B       0.12287
   137B ->145B       0.10039
   140B ->141B       0.10275
   140B ->143B      -0.11130

Excited State  12:  3.042-A     3.6312 eV  341.44 nm  f=0.0405  <S**2>=2.063
   132A ->142A      -0.12234
   134A ->142A      -0.15117
   135A ->148A      -0.19584
   136A ->144A       0.11623
   138A ->142A       0.12388
   139A ->145A      -0.11902
   139A ->146A      -0.14387
   140A ->142A      -0.31321
   140A ->143A       0.10696
   141A ->142A      -0.32523
   141A ->147A       0.11295
   131B ->141B       0.23123
   132B ->141B       0.31189
   134B ->148B      -0.19375
   135B ->142B       0.14638
   137B ->144B       0.10851
   138B ->142B      -0.14745
   139B ->145B       0.10884
   139B ->146B       0.15537
   140B ->142B       0.35685
   140B ->143B      -0.11503

Excited State  13:  2.959-A     3.7323 eV  332.19 nm  f=0.0169  <S**2>=1.939
   132A ->142A      -0.16952
   134A ->143A       0.13509
   135A ->148A      -0.21375
   136A ->144A      -0.10667
   138A ->144A       0.12026
   138A ->146A       0.10948
   139A ->145A       0.13930
   139A ->146A       0.16262
   140A ->147A      -0.11660
   131B ->141B       0.53531
   131B ->142B       0.14127
   132B ->141B      -0.31069
   132B ->142B      -0.12780
   134B ->148B      -0.20726
   135B ->143B      -0.12798
   138B ->144B      -0.11399
   139B ->145B      -0.11245
   139B ->146B      -0.15829
   140B ->147B      -0.10826

Excited State  14:  2.274-A     3.7631 eV  329.48 nm  f=0.0101  <S**2>=1.043
   141A ->144A       0.93989

Excited State  15:  3.121-A     3.8343 eV  323.36 nm  f=0.0184  <S**2>=2.185
   134A ->143A      -0.13555
```

```
136A ->144A        0.13725
136A ->145A        0.19583
137A ->144A       -0.20268
137A ->145A       -0.16082
138A ->144A       -0.16505
138A ->146A       -0.12270
139A ->144A       -0.17582
139A ->146A        0.21554
140A ->142A        0.13728
140A ->143A        0.10856
140A ->145A        0.10959
141A ->142A        0.25006
141A ->143A       -0.26618
131B ->141B        0.28010
132B ->141B        0.16082
135B ->143B        0.14477
135B ->144B        0.11047
136B ->145B       -0.10376
137B ->144B        0.22403
137B ->145B        0.22324
138B ->144B        0.13843
138B ->146B        0.12231
139B ->144B        0.17963
139B ->146B       -0.22111
140B ->142B       -0.11406
```

4Py$_2$PyBTM

```
Excitation energies and oscillator strengths:

 Excited State   1:  2.200-A     2.4267 eV  510.92 nm  f=0.0592  <S**2>=0.960
    153A ->154A      -0.25487
    141B ->153B       0.11916
    152B ->153B       0.92431
 This state for optimization and/or second-order correction.
 Total Energy, E(TD-HF/TD-DFT) =  -4000.72797769
 Copying the excited state density for this state as the 1-particle RhoCI density.

 Excited State   2:  2.181-A     2.5794 eV  480.68 nm  f=0.0066  <S**2>=0.939
    153A ->155A       0.22986
    142B ->153B      -0.11402
    148B ->153B       0.41809
    151B ->153B       0.83121

 Excited State   3:  2.102-A     2.6734 eV  463.77 nm  f=0.0100  <S**2>=0.855
    147B ->153B       0.22309
    150B ->153B       0.94937
    152B ->153B       0.10190

 Excited State   4:  2.141-A     2.7015 eV  458.95 nm  f=0.0108  <S**2>=0.896
    153A ->154A      -0.13626
    149B ->153B       0.95885
    152B ->153B      -0.11125

 Excited State   5:  2.198-A     2.7031 eV  458.67 nm  f=0.0101  <S**2>=0.958
    152A ->154A      -0.12411
    153A ->155A       0.27125
    146B ->153B      -0.38513
    148B ->153B       0.66465
    151B ->153B      -0.49798
    152B ->154B      -0.11131

 Excited State   6:  2.408-A     3.0425 eV  407.51 nm  f=0.3126  <S**2>=1.199
    152A ->155A      -0.14109
    153A ->154A       0.79314
    143B ->153B      -0.30634
    148B ->154B       0.13120
    149B ->153B       0.18337
    150B ->153B      -0.10175
    152B ->153B       0.27625
    152B ->155B      -0.14453

 Excited State   7:  2.346-A     3.1121 eV  398.40 nm  f=0.0339  <S**2>=1.126
    144A ->155A      -0.14695
    149A ->156A      -0.10731
    149A ->158A      -0.13454
    153A ->155A      -0.44736
    153A ->157A       0.25658
    140B ->153B       0.20075
    142B ->153B       0.65320
    142B ->155B      -0.11757
    146B ->153B      -0.17123
    148B ->153B       0.19653
    149B ->156B       0.10645
    149B ->158B       0.13168

 Excited State   8:  2.229-A     3.1701 eV  391.10 nm  f=0.0937  <S**2>=0.993
    153A ->154A       0.28884
    141B ->153B       0.10400
    143B ->153B       0.89690
    143B ->155B      -0.21115
    152B ->153B       0.10452

 Excited State   9:  2.436-A     3.1798 eV  389.91 nm  f=0.1433  <S**2>=1.233
    152A ->154A      -0.18440
    153A ->155A       0.63884
    142B ->153B       0.38686
    146B ->153B      -0.20586
    148B ->153B      -0.45366
    152B ->154B      -0.19599

 Excited State  10:  2.195-A     3.2080 eV  386.49 nm  f=0.0014  <S**2>=0.955
    146B ->154B       0.13583
```

```
   147B ->153B       0.94352
   150B ->153B      -0.21346

Excited State  11:  2.249-A     3.2130 eV  385.89 nm  f=0.0221  <S**2>=1.015
   153A ->155A       0.21995
   142B ->153B       0.22673
   146B ->153B       0.84792
   147B ->154B       0.14316
   148B ->153B       0.26011
   151B ->153B      -0.15073

Excited State  12:  2.155-A     3.3168 eV  373.80 nm  f=0.0016  <S**2>=0.911
   144B ->153B       0.98026
   145B ->154B      -0.11486

Excited State  13:  2.159-A     3.3175 eV  373.73 nm  f=0.0048  <S**2>=0.916
   144B ->154B      -0.11525
   145B ->153B       0.97520

Excited State  14:  2.997-A     3.5782 eV  346.50 nm  f=0.0289  <S**2>=1.995
   144A ->155A       0.16981
   145A ->160A       0.11438
   146A ->161A       0.10084
   150A ->156A       0.15360
   151A ->159A       0.16145
   152A ->154A       0.35006
   153A ->155A       0.35063
   153A ->157A      -0.13690
   140B ->153B       0.14826
   142B ->153B       0.38714
   144B ->160B       0.11339
   145B ->161B       0.11294
   148B ->153B       0.12863
   148B ->155B      -0.21022
   149B ->154B       0.10536
   150B ->156B      -0.14597
   151B ->157B       0.14762
   152B ->154B       0.37085

Excited State  15:  2.999-A     3.6533 eV  339.38 nm  f=0.0728  <S**2>=1.998
   141A ->157A      -0.10568
   142A ->154A       0.17274
   144A ->154A      -0.21266
   145A ->161A       0.20094
   146A ->154A      -0.10221
   146A ->160A       0.18455
   152A ->155A      -0.23431
   152A ->157A      -0.11538
   153A ->154A      -0.29530
   153A ->162A      -0.12024
   140B ->154B      -0.10925
   141B ->153B       0.50397
   142B ->154B       0.11044
   144B ->161B       0.19975
   145B ->160B       0.19955
   146B ->154B      -0.11731
   148B ->154B       0.26051
   152B ->155B      -0.21975
   152B ->159B       0.10986
```

MeO3PyPyBTM

```
Excitation energies and oscillator strengths:

 Excited State   1:  2.135-A     2.2227 eV  557.82 nm  f=0.0990  <S**2>=0.889
    149A ->150A      -0.15218
    143B ->149B      -0.13111
    145B ->149B      -0.14189
    146B ->149B       0.16914
    148B ->149B       0.92503
 This state for optimization and/or second-order correction.
 Total Energy, E(TD-HF/TD-DFT) =  -4327.76278843
 Copying the excited state density for this state as the 1-particle RhoCI density.

 Excited State   2:  2.100-A     2.5852 eV  479.58 nm  f=0.0027  <S**2>=0.853
    143B ->149B      -0.14238
    144B ->149B      -0.12196
    147B ->149B       0.95567

 Excited State   3:  2.142-A     2.6827 eV  462.16 nm  f=0.0105  <S**2>=0.897
    149A ->151A       0.13265
    143B ->149B       0.21546
    144B ->149B       0.24345
    146B ->149B       0.87520
    147B ->149B       0.18233
    148B ->149B      -0.14510

 Excited State   4:  2.175-A     2.7612 eV  449.02 nm  f=0.0199  <S**2>=0.933
    149A ->151A       0.20640
    143B ->149B       0.28956
    144B ->149B       0.74326
    145B ->149B      -0.29444
    146B ->149B      -0.36638
    147B ->149B       0.14225

 Excited State   5:  2.107-A     2.8220 eV  439.34 nm  f=0.0081  <S**2>=0.860
    149A ->150A       0.11426
    144B ->149B       0.29453
    145B ->149B       0.90294
    148B ->149B       0.16068

 Excited State   6:  2.320-A     2.9115 eV  425.84 nm  f=0.0309  <S**2>=1.096
    149A ->150A       0.51800
    140B ->149B       0.17196
    142B ->149B      -0.18144
    143B ->149B       0.58030
    144B ->149B      -0.36645
    148B ->149B       0.27092
    148B ->150B      -0.10716

 Excited State   7:  2.688-A     3.0813 eV  402.37 nm  f=0.1561  <S**2>=1.556
    148A ->150A       0.21679
    148A ->155A      -0.10162
    149A ->150A       0.49612
    149A ->151A      -0.34959
    140B ->149B      -0.23915
    141B ->149B       0.26755
    142B ->149B       0.15191
    143B ->149B      -0.34309
    144B ->149B       0.24210
    145B ->149B      -0.12526
    148B ->150B      -0.29590

 Excited State   8:  2.352-A     3.1912 eV  388.52 nm  f=0.0231  <S**2>=1.133
    146A ->153A       0.10249
    148A ->150A      -0.12816
    149A ->150A       0.19476
    149A ->151A       0.29635
    149A ->155A       0.11335
    139B ->149B      -0.16997
    140B ->149B       0.55306
    141B ->149B       0.46283
    143B ->149B      -0.30576
    146B ->153B      -0.10729

 Excited State   9:  2.270-A     3.2082 eV  386.46 nm  f=0.0551  <S**2>=1.038
    148A ->151A       0.10678
```

```
   149A ->150A      -0.32439
   149A ->151A      -0.25583
   139B ->149B       0.16199
   141B ->149B       0.72658
   141B ->150B      -0.13212
   141B ->151B      -0.10731
   143B ->149B       0.38566

Excited State  10:  2.415-A     3.3289 eV  372.45 nm  f=0.1382  <S**2>=1.208
   145A ->152A       0.14434
   148A ->151A      -0.15188
   149A ->150A       0.11755
   149A ->151A       0.64371
   149A ->155A      -0.10129
   139B ->149B       0.15705
   140B ->149B      -0.41821
   141B ->149B       0.24999
   142B ->149B       0.11498
   144B ->149B      -0.20219
   144B ->151B       0.12268
   145B ->149B       0.10925
   145B ->152B      -0.12534

Excited State  11:  2.848-A     3.5167 eV  352.56 nm  f=0.1268  <S**2>=1.778
   148A ->150A      -0.40332
   148A ->151A       0.19147
   148A ->157A       0.10021
   149A ->150A       0.41191
   149A ->155A       0.19882
   139B ->149B       0.11762
   140B ->149B      -0.29082
   142B ->149B       0.18996
   148B ->150B       0.46130
   148B ->151B      -0.16746
   148B ->155B      -0.13014
   148B ->157B      -0.15954

Excited State  12:  2.142-A     3.5636 eV  347.92 nm  f=0.0049  <S**2>=0.898
   139B ->149B      -0.10719
   140B ->149B       0.12041
   141B ->149B      -0.12715
   142B ->149B       0.91378
   142B ->150B       0.10054
   143B ->149B       0.25792

Excited State  13:  2.247-A     3.7082 eV  334.35 nm  f=0.0099  <S**2>=1.012
   148A ->152A      -0.17311
   149A ->152A       0.93912

Excited State  14:  3.136-A     3.7694 eV  328.92 nm  f=0.0096  <S**2>=2.208
   144A ->151A      -0.14448
   144A ->152A       0.13420
   144A ->153A       0.13540
   145A ->152A       0.29205
   146A ->151A      -0.10594
   146A ->153A      -0.15400
   147A ->154A       0.23172
   148A ->150A      -0.11351
   148A ->155A       0.10381
   149A ->151A      -0.22450
   139B ->149B       0.12701
   140B ->149B       0.38727
   142B ->149B       0.10671
   143B ->149B      -0.13818
   143B ->150B       0.10447
   143B ->151B       0.10250
   144B ->151B       0.15652
   144B ->152B      -0.15523
   144B ->153B      -0.13158
   145B ->152B      -0.25715
   146B ->153B       0.13999
   147B ->154B      -0.21009
   148B ->155B      -0.10386
   148B ->157B      -0.10567

Excited State  15:  3.159-A     3.8372 eV  323.11 nm  f=0.0187  <S**2>=2.245
   144A ->152A      -0.20928
```

```
145A ->152A      -0.14916
145A ->153A      -0.24795
146A ->151A       0.10350
146A ->152A       0.19103
146A ->153A      -0.18942
147A ->154A       0.20099
149A ->151A       0.35978
149A ->154A      -0.17541
139B ->149B       0.19477
140B ->149B       0.18987
144B ->152B       0.21434
145B ->152B       0.13148
145B ->153B       0.23716
146B ->152B      -0.18934
146B ->153B       0.21215
147B ->154B      -0.20063
148B ->150B      -0.12335
```

MeO3Py$_2$PyBTM

```
Excitation energies and oscillator strengths:

 Excited State   1:  2.142-A     2.1980 eV  564.08 nm  f=0.1375  <S**2>=0.897
    169A ->170A      -0.16842
    163B ->169B      -0.22078
    164B ->169B       0.12024
    165B ->169B      -0.10390
    168B ->169B       0.92713
 This state for optimization and/or second-order correction.
 Total Energy, E(TD-HF/TD-DFT) =  -4229.80895651
 Copying the excited state density for this state as the 1-particle RhoCI density.

 Excited State   2:  2.154-A     2.3634 eV  524.60 nm  f=0.0302  <S**2>=0.910
    168A ->170A       0.10935
    169A ->171A      -0.17003
    162B ->169B      -0.23943
    166B ->169B       0.10695
    167B ->169B       0.91772
    168B ->170B       0.10191

 Excited State   3:  2.087-A     2.6407 eV  469.51 nm  f=0.0022  <S**2>=0.839
    166B ->169B       0.98034
    167B ->169B      -0.11616

 Excited State   4:  2.087-A     2.6636 eV  465.47 nm  f=0.0093  <S**2>=0.839
    163B ->169B      -0.10047
    165B ->169B       0.97600

 Excited State   5:  2.128-A     2.7565 eV  449.78 nm  f=0.0129  <S**2>=0.882
    163B ->169B       0.14784
    164B ->169B       0.96194
    164B ->171B      -0.10216

 Excited State   6:  2.414-A     2.9059 eV  426.66 nm  f=0.1583  <S**2>=1.206
    168A ->171A      -0.13175
    169A ->170A       0.64311
    162B ->170B       0.10348
    163B ->169B       0.55040
    167B ->170B      -0.10201
    168B ->169B       0.33797
    168B ->171B      -0.10572

 Excited State   7:  2.337-A     2.9096 eV  426.13 nm  f=0.0212  <S**2>=1.116
    168A ->170A      -0.11962
    169A ->171A       0.59841
    158B ->169B       0.22692
    160B ->169B      -0.19029
    162B ->169B       0.54783
    167B ->169B       0.32252
    168B ->170B      -0.11229

 Excited State   8:  2.425-A     3.0493 eV  406.60 nm  f=0.1472  <S**2>=1.220
    168A ->171A       0.15462
    168A ->173A      -0.11876
    169A ->170A      -0.50590
    157B ->169B      -0.11453
    159B ->169B      -0.20498
    161B ->169B      -0.11794
    163B ->169B       0.71276
    167B ->170B       0.17107
    168B ->171B       0.14703

 Excited State   9:  2.748-A     3.1278 eV  396.40 nm  f=0.1063  <S**2>=1.637
    167A ->171A       0.10409
    168A ->170A       0.31039
    169A ->171A      -0.46170
    169A ->173A       0.13569
    158B ->169B       0.35593
    160B ->169B      -0.16915
    162B ->169B       0.48957
    167B ->171B       0.17834
    168B ->170B       0.30118

 Excited State  10:  2.378-A     3.2264 eV  384.28 nm  f=0.0335  <S**2>=1.164
    162A ->171A      -0.13193
```

```
    164A ->172A       0.11972
    167A ->171A      -0.13310
    168A ->170A      -0.14616
    169A ->171A      -0.36096
    169A ->173A      -0.22803
    156B ->169B       0.24542
    158B ->169B      -0.47786
    162B ->169B       0.52295
    164B ->172B      -0.12009
    167B ->169B       0.15710
    168B ->170B      -0.10900

 Excited State  11:  2.278-A     3.2578 eV  380.58 nm  f=0.0306  <S**2>=1.048
    159A ->171A       0.10451
    169A ->170A      -0.13401
    157B ->169B      -0.13750
    159B ->169B       0.88910
    159B ->171B      -0.21027
    161B ->169B       0.21056
    163B ->169B       0.14128

 Excited State  12:  2.753-A     3.4462 eV  359.77 nm  f=0.0582  <S**2>=1.645
    167A ->171A      -0.19578
    167A ->173A       0.12903
    168A ->170A      -0.33155
    169A ->171A      -0.41417
    169A ->173A      -0.15760
    156B ->169B      -0.19175
    158B ->169B       0.48917
    160B ->169B      -0.17650
    167B ->171B      -0.22716
    167B ->173B       0.14487
    167B ->177B       0.10236
    168B ->170B      -0.37547

 Excited State  13:  3.020-A     3.5301 eV  351.22 nm  f=0.1371  <S**2>=2.030
    167A ->170A       0.36755
    168A ->171A       0.30674
    168A ->173A      -0.19438
    169A ->170A       0.42218
    169A ->176A       0.12565
    169A ->178A       0.13690
    163B ->169B      -0.14451
    167B ->170B       0.42942
    167B ->178B       0.13576
    168B ->171B       0.32285
    168B ->173B      -0.18886
    168B ->177B      -0.13100

 Excited State  14:  2.101-A     3.6211 eV  342.39 nm  f=0.0005  <S**2>=0.854
    159B ->169B      -0.22219
    161B ->169B       0.95253
    163B ->169B       0.11386

 Excited State  15:  2.148-A     3.6392 eV  340.69 nm  f=0.0001  <S**2>=0.903
    158B ->169B       0.17084
    160B ->169B       0.92132
    162B ->169B       0.24865
```

3PyPyBTM+TsOH@3Py

```
Excitation energies and oscillator strengths:

 Excited State   1:  2.208-A     2.4940 eV  497.14 nm  f=0.0253  <S**2>=0.969
    186A ->187A      -0.21448
    186A ->188A      -0.15139
    179B ->186B       0.20358
    180B ->186B       0.32672
    181B ->186B      -0.19303
    182B ->186B       0.80331
    185B ->186B       0.10793
 This state for optimization and/or second-order correction.
 Total Energy, E(TD-HF/TD-DFT) =  -5108.62498965
 Copying the excited state density for this state as the 1-particle RhoCI density.

 Excited State   2:  2.075-A     2.5549 eV  485.28 nm  f=0.0001  <S**2>=0.826
    182B ->186B      -0.11212
    185B ->186B       0.99074

 Excited State   3:  2.198-A     2.6264 eV  472.07 nm  f=0.0037  <S**2>=0.958
    186A ->187A      -0.23054
    186A ->188A      -0.15464
    186A ->189A       0.11223
    178B ->186B       0.40976
    180B ->186B       0.56754
    181B ->186B       0.51845
    182B ->186B      -0.26364

 Excited State   4:  2.141-A     2.6510 eV  467.70 nm  f=0.0165  <S**2>=0.896
    186A ->187A       0.11095
    179B ->186B      -0.20496
    180B ->186B      -0.38704
    181B ->186B       0.72485
    182B ->186B       0.45821

 Excited State   5:  2.083-A     2.7098 eV  457.53 nm  f=0.0006  <S**2>=0.834
    184B ->186B       0.99296

 Excited State   6:  2.126-A     2.7507 eV  450.73 nm  f=0.0148  <S**2>=0.880
    186A ->189A      -0.11582
    178B ->186B      -0.27034
    179B ->186B       0.87537
    180B ->186B      -0.10961
    181B ->186B       0.27905

 Excited State   7:  2.182-A     2.7724 eV  447.20 nm  f=0.0194  <S**2>=0.941
    186A ->187A      -0.29807
    186A ->188A      -0.17201
    186A ->189A       0.12033
    178B ->186B       0.61312
    179B ->186B       0.20590
    180B ->186B      -0.58844
    181B ->186B      -0.18017

 Excited State   8:  2.072-A     2.9399 eV  421.74 nm  f=0.0000  <S**2>=0.823
    183B ->186B       0.99959

 Excited State   9:  2.223-A     2.9641 eV  418.28 nm  f=0.2230  <S**2>=0.986
    186A ->187A       0.73287
    186A ->188A       0.12158
    186A ->189A       0.13653
    174B ->186B       0.19326
    176B ->186B       0.19589
    178B ->186B       0.42738
    179B ->186B       0.22706
    180B ->186B       0.13716
    181B ->186B      -0.11235

 Excited State  10:  2.235-A     3.1034 eV  399.52 nm  f=0.0307  <S**2>=0.999
    186A ->187A      -0.23457
    176B ->186B       0.91071
    176B ->188B       0.10068
    176B ->189B      -0.16259

 Excited State  11:  2.401-A     3.1503 eV  393.57 nm  f=0.0239  <S**2>=1.191
    178A ->189A       0.15514
```

```
    182A ->193A      -0.11961
    186A ->187A      -0.16498
    186A ->188A      -0.15700
    186A ->189A       0.26932
    186A ->192A      -0.14727
    186A ->198A       0.13671
    174B ->186B       0.74364
    178B ->186B      -0.19606
    182B ->193B       0.10624

 Excited State  12:  2.323-A     3.2851 eV  377.41 nm  f=0.0784  <S**2>=1.099
    186A ->187A      -0.30439
    186A ->188A       0.83701
    174B ->186B       0.16894
    176B ->186B      -0.10735
    178B ->186B       0.19512

 Excited State  13:  2.538-A     3.3613 eV  368.86 nm  f=0.1582  <S**2>=1.361
    179A ->190A       0.12294
    179A ->191A      -0.11357
    186A ->188A       0.11459
    186A ->189A       0.75133
    186A ->192A       0.11883
    174B ->186B      -0.20196
    176B ->186B      -0.12532
    178B ->186B      -0.23246
    179B ->186B       0.12147
    181B ->186B      -0.10494

 Excited State  14:  3.137-A     3.5611 eV  348.16 nm  f=0.0157  <S**2>=2.211
    173A ->187A       0.16907
    178A ->187A       0.18049
    178A ->188A       0.10123
    179A ->187A       0.15600
    180A ->187A       0.19814
    181A ->190A       0.11026
    181A ->191A       0.13430
    182A ->187A       0.22972
    182A ->188A       0.11424
    184A ->187A      -0.13478
    185A ->187A       0.21829
    186A ->187A       0.20565
    186A ->188A       0.14297
    186A ->189A      -0.11978
    172B ->186B      -0.18869
    172B ->187B       0.10877
    174B ->186B       0.28346
    174B ->187B       0.12076
    178B ->187B      -0.25946
    178B ->188B      -0.14447
    179B ->187B      -0.13829
    180B ->187B      -0.22623
    180B ->188B      -0.10645
    181B ->190B      -0.10345
    181B ->191B      -0.12261
    182B ->187B      -0.19877
    182B ->188B      -0.10503
    185B ->187B      -0.13782

 Excited State  15:  2.923-A     3.6305 eV  341.51 nm  f=0.0028  <S**2>=1.886
    184A ->187A      -0.15861
    185A ->187A       0.91442
    185A ->195A      -0.11448
    177B ->186B       0.11180
    185B ->195B       0.11094
```

3PyPyBTM+TsOH@PyBTM

```
 Excitation energies and oscillator strengths:

 Excited State   1:  2.142-A     2.3322 eV  531.63 nm  f=0.0877  <S**2>=0.897
    186A ->187A      -0.10954
    186A ->188A      -0.15818
    177B ->186B       0.17236
    179B ->186B      -0.14578
    185B ->186B       0.92293
 This state for optimization and/or second-order correction.
 Total Energy, E(TD-HF/TD-DFT) =  -5108.62539098
 Copying the excited state density for this state as the 1-particle RhoCI density.

 Excited State   2:  2.092-A     2.5230 eV  491.42 nm  f=0.0033  <S**2>=0.844
    179B ->186B       0.17214
    183B ->186B       0.95684

 Excited State   3:  2.160-A     2.6632 eV  465.55 nm  f=0.0202  <S**2>=0.916
    186A ->187A      -0.21110
    186A ->188A       0.10093
    177B ->186B       0.16166
    178B ->186B      -0.21785
    179B ->186B       0.76460
    180B ->186B      -0.34708
    181B ->186B       0.19817
    183B ->186B      -0.21680

 Excited State   4:  2.091-A     2.7231 eV  455.30 nm  f=0.0066  <S**2>=0.843
    186A ->187A      -0.10182
    179B ->186B       0.26371
    180B ->186B       0.90759
    181B ->186B       0.22323

 Excited State   5:  2.177-A     2.8002 eV  442.77 nm  f=0.0165  <S**2>=0.935
    179A ->187A       0.11193
    178B ->186B       0.91885
    179B ->186B       0.28880

 Excited State   6:  2.067-A     2.9033 eV  427.04 nm  f=0.0000  <S**2>=0.818
    184B ->186B       0.99700

 Excited State   7:  2.267-A     2.9954 eV  413.92 nm  f=0.1493  <S**2>=1.035
    186A ->187A       0.78081
    174B ->186B      -0.13516
    175B ->186B       0.27363
    177B ->186B      -0.16972
    178B ->186B      -0.13790
    179B ->186B       0.19718
    181B ->186B       0.19335
    185B ->186B       0.22007

 Excited State   8:  2.094-A     3.0978 eV  400.24 nm  f=0.0032  <S**2>=0.846
    186A ->187A      -0.10184
    179B ->186B      -0.30063
    180B ->186B      -0.14225
    181B ->186B       0.91022

 Excited State   9:  2.071-A     3.1440 eV  394.36 nm  f=0.0017  <S**2>=0.823
    182B ->186B       0.99453

 Excited State  10:  2.692-A     3.1617 eV  392.14 nm  f=0.1380  <S**2>=1.561
    183A ->191A      -0.11313
    184A ->187A      -0.13206
    185A ->187A       0.13991
    185A ->188A       0.10223
    186A ->187A       0.21501
    186A ->188A       0.53487
    174B ->186B       0.13756
    175B ->186B      -0.40854
    177B ->186B       0.31409
    181B ->186B      -0.12253
    185B ->186B       0.12584
    185B ->187B      -0.24264
    185B ->188B       0.16518

 Excited State  11:  2.392-A     3.2733 eV  378.78 nm  f=0.1412  <S**2>=1.181
```

```
    180A ->190A       0.13712
    186A ->187A      -0.33343
    186A ->188A       0.56354
    186A ->194A      -0.10547
    175B ->186B       0.33629
    177B ->186B      -0.46231
    178B ->186B       0.11110
    179B ->188B       0.10899
    180B ->190B      -0.11850
    185B ->186B       0.17917

 Excited State  12:  2.379-A     3.3987 eV  364.80 nm  f=0.0017  <S**2>=1.165
    177A ->187A       0.10200
    178A ->188A       0.11358
    186A ->188A       0.12004
    174B ->186B      -0.44405
    175B ->186B       0.30904
    177B ->186B       0.65665
    179B ->186B      -0.16503
    179B ->188B       0.10648
    185B ->186B      -0.15777

 Excited State  13:  3.007-A     3.6011 eV  344.29 nm  f=0.0611  <S**2>=2.010
    178A ->194A       0.10205
    180A ->189A      -0.12456
    180A ->190A      -0.19519
    183A ->191A      -0.12731
    184A ->187A      -0.13282
    184A ->188A      -0.23346
    185A ->187A       0.13476
    185A ->188A       0.23648
    186A ->187A      -0.30264
    186A ->188A      -0.14759
    186A ->194A       0.15738
    174B ->186B      -0.21283
    175B ->186B       0.28825
    176B ->186B       0.13065
    179B ->186B       0.12059
    179B ->187B      -0.11564
    180B ->189B       0.11265
    180B ->190B       0.17425
    183B ->191B       0.10832
    185B ->187B      -0.28657
    185B ->188B       0.31384
    185B ->194B      -0.10878

 Excited State  14:  2.180-A     3.6327 eV  341.30 nm  f=0.0148  <S**2>=0.938
    170B ->186B       0.22530
    172B ->186B      -0.11411
    173B ->186B       0.24612
    176B ->186B       0.88921
    176B ->187B      -0.10702

 Excited State  15:  3.475-A     3.6599 eV  338.76 nm  f=0.0000  <S**2>=2.769
    182A ->196A       0.40410
    184A ->193A      -0.40626
    185A ->193A      -0.40266
    182B ->196B      -0.39971
    184B ->192B       0.10896
    184B ->193B       0.56253
```

4PyPyBTM+TsOH@4Py

```
 Excitation energies and oscillator strengths:

 Excited State   1:  2.172-A      2.4524 eV  505.57 nm  f=0.0063  <S**2>=0.930
    186A ->187A       0.26169
    178B ->186B      -0.14874
    179B ->186B       0.16417
    180B ->186B      -0.22256
    182B ->186B      -0.46913
    185B ->186B       0.75227
 This state for optimization and/or second-order correction.
 Total Energy, E(TD-HF/TD-DFT) =  -5108.62786062
 Copying the excited state density for this state as the 1-particle RhoCI density.

 Excited State   2:  2.174-A      2.4706 eV  501.84 nm  f=0.0049  <S**2>=0.932
    186A ->187A      -0.28280
    178B ->186B       0.13236
    179B ->186B      -0.16658
    180B ->186B       0.25044
    182B ->186B       0.57313
    185B ->186B       0.65335

 Excited State   3:  2.224-A      2.5803 eV  480.50 nm  f=0.0301  <S**2>=0.986
    186A ->187A       0.48268
    186A ->188A      -0.12335
    178B ->186B      -0.41314
    179B ->186B       0.15816
    180B ->186B      -0.10680
    181B ->186B       0.50591
    182B ->186B       0.44953

 Excited State   4:  2.131-A      2.6042 eV  476.09 nm  f=0.0008  <S**2>=0.885
    184B ->186B       0.98612
    184B ->187B       0.10785

 Excited State   5:  2.131-A      2.6269 eV  471.97 nm  f=0.0118  <S**2>=0.885
    186A ->187A      -0.11867
    179B ->186B      -0.12104
    180B ->186B       0.58012
    181B ->186B       0.61373
    182B ->186B      -0.43689
    184B ->186B      -0.10224

 Excited State   6:  2.108-A      2.6919 eV  460.58 nm  f=0.0399  <S**2>=0.861
    186A ->187A       0.35910
    178B ->186B      -0.20820
    179B ->186B       0.16471
    180B ->186B       0.69021
    181B ->186B      -0.52581

 Excited State   7:  2.163-A      2.7294 eV  454.25 nm  f=0.0455  <S**2>=0.920
    186A ->187A      -0.20371
    186A ->188A       0.11535
    178B ->186B       0.25216
    179B ->186B       0.89516
    181B ->186B       0.14216

 Excited State   8:  2.100-A      2.8588 eV  433.69 nm  f=0.0002  <S**2>=0.853
    183B ->186B       0.99858

 Excited State   9:  2.127-A      2.8919 eV  428.73 nm  f=0.2744  <S**2>=0.881
    186A ->187A       0.56615
    186A ->188A       0.14402
    173B ->186B       0.14824
    178B ->186B       0.72693
    179B ->186B      -0.14547
    181B ->186B       0.14196

 Excited State  10:  2.231-A      3.0841 eV  402.01 nm  f=0.0312  <S**2>=0.995
    186A ->187A       0.12410
    175B ->186B       0.93366
    175B ->187B      -0.14644
    175B ->188B       0.16838

 Excited State  11:  2.461-A      3.1400 eV  394.86 nm  f=0.0217  <S**2>=1.264
    177A ->187A       0.11092
```

```
   177A ->188A      -0.14838
   182A ->193A       0.12820
   186A ->187A      -0.16881
   186A ->188A       0.27971
   186A ->191A       0.12442
   186A ->197A      -0.14444
   173B ->186B       0.73709
   174B ->186B       0.18217
   178B ->186B      -0.13399
   178B ->187B      -0.11323
   182B ->193B      -0.12894

Excited State  12:  2.614-A     3.3527 eV  369.81 nm  f=0.1298  <S**2>=1.458
   181A ->190A       0.12196
   181A ->192A       0.16165
   185A ->187A       0.11342
   186A ->188A       0.72558
   186A ->191A      -0.15996
   173B ->186B      -0.17295
   178B ->186B      -0.25872
   179B ->186B      -0.10870
   181B ->192B       0.12389
   182B ->187B       0.10003

Excited State  13:  2.896-A     3.3827 eV  366.52 nm  f=0.0043  <S**2>=1.847
   184A ->187A      -0.23612
   185A ->187A       0.95115
   186A ->188A      -0.10113

Excited State  14:  2.955-A     3.4224 eV  362.27 nm  f=0.0004  <S**2>=1.933
   184A ->187A       0.95441
   185A ->187A       0.24643

Excited State  15:  2.712-A     3.4683 eV  357.48 nm  f=0.0043  <S**2>=1.589
   173B ->186B      -0.11147
   184B ->187B      -0.15729
   185B ->187B       0.93810
```

4PyPyBTM+TsOH@PyBTM

```
 Excitation energies and oscillator strengths:

 Excited State   1:  2.159-A     2.4067 eV  515.16 nm  f=0.0651  <S**2>=0.916
    186A ->187A       0.13750
    186A ->188A      -0.16143
    183B ->186B      -0.15904
    184B ->186B       0.92669
 This state for optimization and/or second-order correction.
 Total Energy, E(TD-HF/TD-DFT) =  -5108.62267255
 Copying the excited state density for this state as the 1-particle RhoCI density.

 Excited State   2:  2.099-A     2.5268 eV  490.67 nm  f=0.0033  <S**2>=0.852
    179B ->186B      -0.11603
    181B ->186B      -0.30866
    183B ->186B       0.89798
    184B ->186B       0.16363

 Excited State   3:  2.149-A     2.6621 eV  465.74 nm  f=0.0184  <S**2>=0.905
    186A ->187A      -0.19265
    186A ->188A      -0.10631
    175B ->186B       0.10109
    177B ->186B       0.32424
    178B ->186B       0.48758
    179B ->186B      -0.24700
    180B ->186B       0.61955
    181B ->186B      -0.18787
    183B ->186B      -0.22154

 Excited State   4:  2.127-A     2.7188 eV  456.03 nm  f=0.0077  <S**2>=0.881
    186A ->187A       0.20892
    177B ->186B      -0.25130
    178B ->186B      -0.45235
    179B ->186B       0.29430
    180B ->186B       0.72011
    181B ->186B      -0.13376

 Excited State   5:  2.176-A     2.7911 eV  444.21 nm  f=0.0157  <S**2>=0.934
    178A ->187A      -0.11704
    177B ->186B       0.82896
    178B ->186B      -0.48592

 Excited State   6:  2.067-A     2.8888 eV  429.19 nm  f=0.0001  <S**2>=0.818
    185B ->186B       0.99590

 Excited State   7:  2.222-A     3.0316 eV  408.97 nm  f=0.1798  <S**2>=0.984
    177A ->187A       0.10794
    186A ->187A       0.79111
    186A ->188A       0.10235
    174B ->186B       0.17351
    175B ->186B      -0.27551
    177B ->186B       0.18076
    178B ->186B       0.24631
    179B ->186B      -0.14613
    184B ->186B      -0.14581

 Excited State   8:  2.153-A     3.0586 eV  405.36 nm  f=0.0001  <S**2>=0.909
    180B ->186B       0.24226
    181B ->186B       0.89908
    181B ->187B       0.12038
    183B ->186B       0.26801

 Excited State   9:  2.066-A     3.1291 eV  396.23 nm  f=0.0002  <S**2>=0.817
    182B ->186B       0.99895

 Excited State  10:  2.498-A     3.1595 eV  392.42 nm  f=0.1033  <S**2>=1.309
    177A ->187A       0.11981
    184A ->187A      -0.13572
    186A ->187A      -0.23805
    186A ->188A       0.43435
    174B ->186B       0.12465
    175B ->186B      -0.45700
    176B ->186B       0.10851
    177B ->186B       0.13338
    178B ->186B       0.18017
    178B ->187B       0.10078
```

```
   179B ->186B        0.46460
   184B ->186B        0.15263
   184B ->187B       -0.17819

Excited State  11:  2.268-A      3.1670 eV  391.49 nm  f=0.0369  <S**2>=1.036
   186A ->187A        0.12666
   186A ->188A       -0.29353
   175B ->186B        0.25522
   178B ->186B        0.38534
   179B ->186B        0.74396
   179B ->187B        0.10471
   184B ->187B        0.10996

Excited State  12:  2.513-A      3.3006 eV  375.65 nm  f=0.1370  <S**2>=1.329
   180A ->190A        0.15748
   184A ->188A       -0.11598
   186A ->187A        0.19929
   186A ->188A        0.61257
   186A ->192A        0.11373
   186A ->193A        0.14547
   174B ->186B       -0.20259
   175B ->186B        0.47268
   177B ->186B        0.14240
   177B ->188B       -0.12220
   180B ->190B       -0.13713
   184B ->186B        0.13974

Excited State  13:  2.235-A      3.6009 eV  344.31 nm  f=0.0334  <S**2>=0.999
   186A ->188A       -0.10531
   170B ->186B       -0.19523
   173B ->186B       -0.17112
   174B ->186B       -0.35647
   176B ->186B        0.82645

Excited State  14:  3.046-A      3.6149 eV  342.98 nm  f=0.0358  <S**2>=2.070
   177A ->187A        0.13147
   179A ->194A        0.15269
   180A ->189A       -0.12489
   180A ->190A       -0.18731
   181A ->191A       -0.10109
   183A ->188A        0.12825
   183A ->191A        0.17102
   184A ->187A       -0.20728
   184A ->188A        0.25119
   186A ->187A        0.30687
   186A ->193A       -0.11332
   174B ->186B       -0.18401
   175B ->186B        0.26560
   176B ->186B       -0.20577
   178B ->186B       -0.10354
   178B ->187B        0.13449
   179B ->194B       -0.14181
   180B ->189B        0.11343
   180B ->190B        0.16952
   183B ->191B       -0.18569
   184B ->187B       -0.28275
   184B ->188B        0.26219

Excited State  15:  3.475-A      3.6607 eV  338.69 nm  f=0.0000  <S**2>=2.769
   182A ->196A        0.40467
   185A ->192A       -0.46050
   185A ->193A        0.34077
   182B ->196B       -0.40420
   185B ->192B        0.57207
```

MeO3PyBTM+TsOH@MeO3Py

```
Excitation energies and oscillator strengths:

Excited State   1:  2.213-A     2.4746 eV  501.03 nm  f=0.0449  <S**2>=0.974
   194A ->195A      -0.22133
   194A ->197A      -0.13702
   182B ->194B      -0.13454
   186B ->194B      -0.14940
   190B ->194B       0.89479
This state for optimization and/or second-order correction.
Total Energy, E(TD-HF/TD-DFT) =  -5223.15540794
Copying the excited state density for this state as the 1-particle RhoCI density.

Excited State   2:  2.151-A     2.6285 eV  471.69 nm  f=0.0036  <S**2>=0.907
   194A ->195A       0.14515
   194A ->196A      -0.12528
   182B ->194B       0.12751
   186B ->194B      -0.26866
   188B ->194B       0.31945
   189B ->194B       0.69417
   193B ->194B      -0.46698

Excited State   3:  2.093-A     2.6411 eV  469.45 nm  f=0.0009  <S**2>=0.845
   186B ->194B      -0.10338
   189B ->194B       0.42434
   193B ->194B       0.87266

Excited State   4:  2.140-A     2.6749 eV  463.50 nm  f=0.0124  <S**2>=0.895
   194A ->195A       0.11647
   188B ->194B       0.87973
   189B ->194B      -0.38269

Excited State   5:  2.153-A     2.7426 eV  452.07 nm  f=0.0218  <S**2>=0.909
   194A ->196A       0.13109
   194A ->197A       0.12502
   186B ->194B       0.65115
   187B ->194B      -0.57057
   188B ->194B       0.12207
   189B ->194B       0.34908

Excited State   6:  2.087-A     2.7809 eV  445.84 nm  f=0.0016  <S**2>=0.839
   186B ->194B      -0.22833
   187B ->194B      -0.38179
   192B ->194B       0.87537

Excited State   7:  2.134-A     2.7871 eV  444.85 nm  f=0.0059  <S**2>=0.889
   194A ->195A      -0.18811
   194A ->196A       0.10759
   186B ->194B       0.43151
   187B ->194B       0.67591
   189B ->194B       0.14735
   192B ->194B       0.47412

Excited State   8:  2.422-A     3.0083 eV  412.13 nm  f=0.2462  <S**2>=1.217
   190A ->195A       0.18757
   194A ->195A       0.72929
   194A ->197A       0.17573
   182B ->194B      -0.10449
   184B ->194B       0.32654
   186B ->194B       0.12930
   187B ->194B       0.15429
   188B ->194B      -0.17292
   190B ->194B       0.27281
   190B ->195B       0.19365

Excited State   9:  2.067-A     3.0239 eV  410.02 nm  f=0.0001  <S**2>=0.818
   191B ->194B       0.99936

Excited State  10:  2.304-A     3.1118 eV  398.43 nm  f=0.0547  <S**2>=1.078
   194A ->195A      -0.25422
   194A ->196A       0.33100
   194A ->201A       0.12057
   180B ->194B       0.25797
   181B ->194B      -0.31477
   182B ->194B      -0.18393
   184B ->194B       0.56967
```

```
   186B ->194B      -0.27259
   188B ->194B       0.11646
   189B ->194B       0.10082
   190B ->194B      -0.16860

Excited State  11:  2.333-A     3.1411 eV  394.72 nm  f=0.0118  <S**2>=1.111
   194A ->196A      -0.14073
   180B ->194B      -0.14092
   181B ->194B       0.19362
   182B ->194B       0.64860
   183B ->194B      -0.12868
   184B ->194B       0.56091

Excited State  12:  2.566-A     3.3428 eV  370.90 nm  f=0.1524  <S**2>=1.396
   187A ->198A       0.14544
   187A ->199A      -0.12182
   190A ->195A      -0.16114
   194A ->196A       0.42295
   194A ->197A       0.58026
   194A ->201A      -0.11919
   182B ->194B       0.21253
   184B ->194B      -0.24980
   186B ->194B      -0.22374
   186B ->197B       0.12362
   187B ->198B      -0.11834
   187B ->199B       0.10369
   190B ->194B       0.12487
   190B ->195B      -0.18771

Excited State  13:  2.782-A     3.3828 eV  366.52 nm  f=0.0492  <S**2>=1.685
   182A ->195A      -0.15361
   184A ->195A       0.10922
   186A ->195A      -0.17064
   188A ->195A       0.11417
   190A ->195A       0.21248
   190A ->197A      -0.10754
   194A ->196A       0.44605
   194A ->206A       0.12410
   180B ->194B      -0.39650
   181B ->194B       0.40592
   182B ->195B       0.14965
   184B ->195B      -0.12005
   186B ->194B      -0.12586
   186B ->195B       0.15221
   188B ->195B      -0.12838
   190B ->195B       0.24211
   190B ->201B       0.10175

Excited State  14:  2.717-A     3.5160 eV  352.63 nm  f=0.0155  <S**2>=1.595
   186A ->195A      -0.10707
   188A ->195A       0.11640
   189A ->199A       0.13927
   189A ->200A       0.10089
   190A ->195A       0.30765
   194A ->195A      -0.26272
   194A ->196A       0.14724
   180B ->194B       0.16540
   181B ->194B      -0.13859
   182B ->194B       0.48669
   184B ->194B      -0.24820
   186B ->194B       0.18111
   188B ->194B      -0.11963
   188B ->195B      -0.11952
   189B ->199B      -0.13111
   189B ->200B      -0.10966
   190B ->194B       0.18601
   190B ->195B       0.32063

Excited State  15:  2.609-A     3.5698 eV  347.31 nm  f=0.0049  <S**2>=1.452
   189A ->198A       0.10403
   190A ->195A       0.11037
   190A ->196A      -0.15159
   190A ->197A       0.10548
   194A ->195A      -0.37663
   194A ->196A      -0.44201
   194A ->197A       0.47049
   180B ->194B      -0.16012
```

181B	->194B	0.14519
182B	->194B	-0.27388
186B	->197B	0.10142
190B	->195B	0.13647
190B	->196B	0.17699

MeO3PyBTM+TsOH@PyBTM

```
 Excitation energies and oscillator strengths:

 Excited State   1:  2.103-A      2.0978 eV  591.01 nm  f=0.1107  <S**2>=0.856
    194A ->196A       0.11307
    190B ->194B      -0.21777
    193B ->194B       0.94333
 This state for optimization and/or second-order correction.
 Total Energy, E(TD-HF/TD-DFT) =  -5223.17065629
 Copying the excited state density for this state as the 1-particle RhoCI density.

 Excited State   2:  2.079-A      2.4767 eV  500.60 nm  f=0.0026  <S**2>=0.831
    191B ->194B       0.97907

 Excited State   3:  2.149-A      2.6332 eV  470.84 nm  f=0.0186  <S**2>=0.904
    194A ->195A       0.11474
    194A ->196A      -0.14311
    185B ->194B       0.10482
    186B ->194B      -0.34266
    188B ->194B       0.40071
    190B ->194B       0.75486
    191B ->194B       0.10537
    193B ->194B       0.15854

 Excited State   4:  2.106-A      2.7195 eV  455.91 nm  f=0.0084  <S**2>=0.859
    194A ->195A       0.16458
    186B ->194B      -0.13730
    187B ->194B       0.11792
    188B ->194B       0.77432
    190B ->194B      -0.51475
    193B ->194B      -0.12718

 Excited State   5:  2.203-A      2.8046 eV  442.08 nm  f=0.0202  <S**2>=0.963
    194A ->195A       0.36035
    183B ->194B       0.13225
    185B ->194B       0.17448
    186B ->194B      -0.48949
    187B ->194B       0.55776
    188B ->194B      -0.40488
    193B ->194B      -0.13788

 Excited State   6:  2.265-A      2.8434 eV  436.04 nm  f=0.0126  <S**2>=1.032
    194A ->195A      -0.37435
    194A ->196A      -0.18630
    183B ->194B      -0.13816
    185B ->194B      -0.18677
    186B ->194B       0.25587
    187B ->194B       0.75489
    188B ->194B       0.13299
    193B ->194B       0.16909

 Excited State   7:  2.069-A      2.9524 eV  419.95 nm  f=0.0012  <S**2>=0.821
    192B ->194B       0.99584

 Excited State   8:  2.340-A      3.0285 eV  409.39 nm  f=0.1883  <S**2>=1.119
    193A ->196A       0.12385
    194A ->195A       0.64979
    194A ->196A       0.16033
    182B ->194B      -0.15010
    185B ->194B      -0.20806
    186B ->194B       0.56062
    187B ->194B       0.14849
    190B ->194B       0.17042
    193B ->195B       0.13434
    193B ->196B      -0.14661

 Excited State   9:  2.693-A      3.1054 eV  399.26 nm  f=0.0905  <S**2>=1.563
    185A ->195A       0.13550
    193A ->195A       0.25092
    194A ->195A      -0.19692
    194A ->196A       0.59874
    182B ->194B       0.17117
    183B ->194B      -0.36895
    184B ->194B       0.11765
    186B ->194B      -0.14696
    187B ->194B       0.10451
```

```
   188B ->194B       0.10263
   193B ->194B      -0.10727
   193B ->195B       0.24038
   193B ->196B      -0.17945

Excited State  10:  2.065-A     3.1942 eV  388.16 nm  f=0.0001  <S**2>=0.816
   189B ->194B       0.99771

Excited State  11:  2.505-A     3.2927 eV  376.54 nm  f=0.0862  <S**2>=1.318
   185A ->195A      -0.11976
   188A ->198A       0.11748
   193A ->196A      -0.11749
   194A ->195A      -0.26022
   194A ->196A       0.42246
   194A ->201A      -0.12872
   182B ->194B      -0.31263
   183B ->194B       0.55128
   184B ->194B      -0.12428
   185B ->194B       0.10842
   187B ->197B       0.11946
   188B ->198B      -0.10541
   190B ->194B       0.15765

Excited State  12:  2.108-A     3.3976 eV  364.92 nm  f=0.0016  <S**2>=0.861
   185B ->194B       0.89344
   186B ->194B       0.38389

Excited State  13:  2.892-A     3.4998 eV  354.26 nm  f=0.1360  <S**2>=1.840
   188A ->198A      -0.12756
   193A ->195A       0.22087
   193A ->196A       0.37925
   194A ->195A      -0.25386
   194A ->196A      -0.27611
   194A ->201A       0.21032
   194A ->207A      -0.10065
   182B ->194B      -0.19482
   183B ->194B       0.25351
   184B ->194B      -0.11353
   188B ->198B       0.11266
   190B ->194B      -0.10496
   193B ->195B       0.33967
   193B ->196B      -0.35264
   193B ->201B      -0.14321
   193B ->204B      -0.13858

Excited State  14:  2.179-A     3.6541 eV  339.30 nm  f=0.0199  <S**2>=0.936
   194A ->196A      -0.10509
   178B ->194B      -0.22064
   181B ->194B      -0.23646
   182B ->194B      -0.14944
   183B ->194B       0.13405
   184B ->194B       0.87651
   184B ->195B      -0.10124

Excited State  15:  3.474-A     3.6602 eV  338.74 nm  f=0.0000  <S**2>=2.767
   190A ->203A       0.40386
   192A ->200A      -0.56091
   189B ->203B      -0.40339
   192B ->199B       0.57210
```

3PyBTM+BH_3@3Py

```
Excitation energies and oscillator strengths:

Excited State   1:  2.203-A     2.4914 eV  497.65 nm  f=0.0397  <S**2>=0.963
   145A ->146A      -0.21670
   145A ->148A      -0.11295
   140B ->145B      -0.18618
   142B ->145B      -0.13393
   144B ->145B       0.89416
This state for optimization and/or second-order correction.
Total Energy, E(TD-HF/TD-DFT) =  -4239.88814450
Copying the excited state density for this state as the 1-particle RhoCI density.

Excited State   2:  2.172-A     2.6336 eV  470.78 nm  f=0.0050  <S**2>=0.930
   145A ->146A       0.16856
   145A ->147A       0.10707
   139B ->145B       0.32765
   140B ->145B      -0.13839
   141B ->145B       0.10605
   143B ->145B       0.85624

Excited State   3:  2.139-A     2.6759 eV  463.34 nm  f=0.0167  <S**2>=0.894
   145A ->146A       0.10484
   140B ->145B       0.15011
   142B ->145B       0.92750
   144B ->145B       0.23304

Excited State   4:  2.137-A     2.7549 eV  450.05 nm  f=0.0170  <S**2>=0.891
   145A ->148A      -0.13523
   139B ->145B       0.28853
   140B ->145B      -0.50412
   141B ->145B       0.67392
   142B ->145B       0.13300
   143B ->145B      -0.32330

Excited State   5:  2.157-A     2.7919 eV  444.08 nm  f=0.0080  <S**2>=0.914
   145A ->146A      -0.20133
   145A ->147A      -0.11695
   139B ->145B      -0.47958
   140B ->145B       0.35572
   141B ->145B       0.66564
   143B ->145B       0.27359

Excited State   6:  2.398-A     3.0650 eV  404.52 nm  f=0.1652  <S**2>=1.188
   144A ->146A       0.12890
   145A ->146A       0.60471
   145A ->148A       0.19333
   136B ->145B       0.19634
   138B ->145B      -0.50682
   139B ->145B      -0.23736
   141B ->145B       0.11367
   142B ->145B      -0.15124
   144B ->145B       0.18483
   144B ->146B       0.14175

Excited State   7:  2.298-A     3.1363 eV  395.33 nm  f=0.0839  <S**2>=1.070
   139A ->148A      -0.10900
   142A ->151A       0.12087
   145A ->146A      -0.37537
   145A ->147A      -0.27710
   145A ->148A       0.18244
   145A ->152A      -0.16000
   135B ->145B      -0.18855
   136B ->145B       0.54951
   138B ->145B      -0.26117
   140B ->145B      -0.30350
   142B ->145B       0.10564
   142B ->151B       0.10803
   143B ->145B       0.15313
   144B ->145B      -0.14214

Excited State   8:  2.322-A     3.1472 eV  393.95 nm  f=0.0810  <S**2>=1.098
   145A ->146A       0.30367
   136B ->145B       0.43354
   138B ->145B       0.73703
   138B ->146B       0.10233
```

```
   138B ->148B      -0.11343

Excited State   9:  2.187-A     3.3209 eV  373.35 nm  f=0.0120  <S**2>=0.945
   145A ->148A       0.26397
   135B ->145B      -0.12066
   136B ->145B       0.11405
   139B ->145B       0.65203
   140B ->145B       0.53178
   141B ->145B       0.19789
   142B ->145B      -0.17139
   143B ->145B      -0.14892
   144B ->145B       0.18479

Excited State  10:  2.473-A     3.3545 eV  369.61 nm  f=0.1427  <S**2>=1.279
   141A ->149A      -0.15510
   141A ->150A      -0.12045
   144A ->148A      -0.11988
   145A ->147A      -0.35319
   145A ->148A       0.62694
   145A ->152A       0.11556
   136B ->145B      -0.30454
   138B ->145B       0.16539
   140B ->145B      -0.33316
   140B ->148B       0.11669
   141B ->149B       0.13368
   141B ->150B       0.10667
   144B ->148B      -0.11078

Excited State  11:  3.029-A     3.5133 eV  352.90 nm  f=0.0468  <S**2>=2.044
   136A ->146A       0.15188
   140A ->146A       0.14762
   140A ->147A      -0.16289
   141A ->146A       0.11696
   142A ->146A      -0.19140
   144A ->146A      -0.28745
   145A ->146A       0.17400
   145A ->147A       0.36753
   145A ->152A       0.12356
   135B ->145B      -0.24662
   136B ->145B       0.21050
   139B ->147B       0.13604
   140B ->145B      -0.16590
   140B ->146B      -0.18631
   140B ->147B       0.12960
   141B ->146B      -0.11030
   142B ->146B       0.16664
   143B ->146B       0.16269
   144B ->146B      -0.28833

Excited State  12:  2.594-A     3.6013 eV  344.27 nm  f=0.0031  <S**2>=1.432
   139A ->147A       0.10540
   143A ->149A      -0.11707
   143A ->150A       0.11320
   144A ->147A       0.15431
   145A ->146A      -0.41769
   145A ->147A       0.63316
   145A ->148A       0.32723
   139B ->147B      -0.14534
   144B ->146B       0.10907
   144B ->147B       0.13542

Excited State  13:  2.983-A     3.7057 eV  334.58 nm  f=0.0057  <S**2>=1.974
   139A ->148A       0.11541
   140A ->146A      -0.13714
   140A ->147A       0.10336
   141A ->149A      -0.19752
   142A ->149A      -0.10415
   142A ->151A      -0.11631
   143A ->149A       0.11738
   143A ->150A      -0.24202
   144A ->152A      -0.12694
   145A ->147A       0.22432
   135B ->145B       0.25692
   136B ->145B       0.35729
   137B ->145B       0.32884
   139B ->145B       0.15395
   139B ->148B      -0.10738
```

```
   141B ->149B       0.18393
   142B ->149B       0.13320
   143B ->150B       0.19893
   143B ->151B      -0.12850
   144B ->152B      -0.10903

Excited State  14:  2.236-A     3.7655 eV  329.26 nm  f=0.0018  <S**2>=1.000
   145A ->149A      -0.18056
   136B ->145B      -0.17159
   137B ->145B       0.91209

Excited State  15:  2.279-A     3.7755 eV  328.39 nm  f=0.0101  <S**2>=1.049
   145A ->149A       0.92559
   137B ->145B       0.15180
```

3PyBTM+BH_3@PyBTM

```
Excitation energies and oscillator strengths:

Excited State   1:  2.123-A     2.2713 eV  545.86 nm  f=0.0944  <S**2>=0.877
   145A ->147A       0.14346
   137B ->145B       0.14870
   139B ->145B       0.13818
   144B ->145B       0.92960
This state for optimization and/or second-order correction.
Total Energy, E(TD-HF/TD-DFT) =  -4239.89151077
Copying the excited state density for this state as the 1-particle RhoCI density.

Excited State   2:  2.105-A     2.4412 eV  507.89 nm  f=0.0076  <S**2>=0.858
   145A ->146A       0.13680
   140B ->145B      -0.20429
   141B ->145B       0.12925
   143B ->145B       0.93459

Excited State   3:  2.159-A     2.5624 eV  483.86 nm  f=0.0263  <S**2>=0.915
   145A ->146A      -0.27685
   140B ->145B       0.62953
   141B ->145B      -0.57883
   142B ->145B       0.13431
   143B ->145B       0.29168

Excited State   4:  2.083-A     2.6376 eV  470.06 nm  f=0.0064  <S**2>=0.835
   145A ->146A      -0.12706
   140B ->145B       0.56119
   141B ->145B       0.76601
   142B ->145B       0.20897

Excited State   5:  2.197-A     2.7781 eV  446.29 nm  f=0.0442  <S**2>=0.957
   140A ->146A      -0.12872
   145A ->146A      -0.50655
   145A ->152A       0.10169
   134B ->145B       0.15285
   137B ->145B      -0.12062
   139B ->145B       0.72805
   140B ->145B      -0.11676
   142B ->145B      -0.14570
   144B ->145B      -0.17335

Excited State   6:  2.212-A     2.8651 eV  432.75 nm  f=0.0173  <S**2>=0.973
   139A ->146A      -0.10760
   145A ->146A      -0.13051
   138B ->145B       0.94484
   138B ->146B      -0.11021

Excited State   7:  2.333-A     2.9594 eV  418.95 nm  f=0.2643  <S**2>=1.110
   144A ->147A      -0.14082
   145A ->146A       0.69547
   145A ->147A      -0.12539
   138B ->145B       0.14938
   139B ->145B       0.48506
   140B ->145B       0.27666
   141B ->145B      -0.11293
   144B ->145B       0.10111
   144B ->146B      -0.10074
   144B ->147B       0.12143

Excited State   8:  2.079-A     3.0009 eV  413.15 nm  f=0.0061  <S**2>=0.830
   145A ->146A      -0.10835
   140B ->145B      -0.26205
   142B ->145B       0.93809
   143B ->145B      -0.10901

Excited State   9:  2.671-A     3.1975 eV  387.76 nm  f=0.1738  <S**2>=1.534
   143A ->150A       0.12962
   144A ->146A      -0.19111
   144A ->152A      -0.10960
   145A ->146A       0.15774
   145A ->147A       0.72482
   137B ->145B       0.13405
   139B ->145B       0.14262
   140B ->145B       0.12319
   141B ->145B      -0.11048
```

```
      144B ->145B      -0.22146
      144B ->146B       0.24125
      144B ->147B      -0.14493

 Excited State  10:  2.215-A     3.3005 eV  375.65 nm  f=0.0008  <S**2>=0.977
      145A ->147A      -0.12526
      134B ->145B      -0.10159
      135B ->145B       0.32285
      137B ->145B       0.84933
      139B ->145B       0.11708
      140B ->145B      -0.12982
      144B ->145B      -0.16555

 Excited State  11:  2.119-A     3.4228 eV  362.23 nm  f=0.0021  <S**2>=0.873
      136B ->145B       0.98513
      136B ->146B      -0.12536

 Excited State  12:  2.998-A     3.4878 eV  355.48 nm  f=0.0355  <S**2>=1.997
      139A ->148A       0.11578
      140A ->146A       0.17983
      141A ->149A       0.20622
      143A ->150A       0.10844
      144A ->146A      -0.14020
      144A ->147A       0.26848
      145A ->147A      -0.22187
      145A ->151A      -0.11026
      145A ->152A      -0.17152
      134B ->145B      -0.31340
      135B ->145B      -0.21073
      138B ->148B      -0.12959
      139B ->145B       0.33831
      140B ->146B       0.16762
      140B ->147B       0.11949
      141B ->149B      -0.18261
      144B ->146B       0.19291
      144B ->147B      -0.24617

 Excited State  13:  2.201-A     3.6078 eV  343.65 nm  f=0.0085  <S**2>=0.961
      145A ->147A      -0.18974
      145A ->148A       0.91173
      134B ->145B       0.15932

 Excited State  14:  2.659-A     3.6353 eV  341.06 nm  f=0.0204  <S**2>=1.518
      138A ->148A       0.10659
      139A ->148A      -0.11754
      141A ->149A       0.14994
      144A ->147A       0.22329
      145A ->146A       0.19796
      145A ->148A      -0.24234
      134B ->145B       0.55858
      135B ->145B       0.36291
      138B ->148B       0.16741
      140B ->145B      -0.14662
      141B ->149B      -0.13018
      144B ->146B       0.15578
      144B ->147B      -0.22113

 Excited State  15:  2.565-A     3.7303 eV  332.37 nm  f=0.0080  <S**2>=1.395
      138A ->148A      -0.12883
      139A ->148A       0.13815
      140A ->146A       0.14439
      143A ->150A       0.10481
      134B ->145B      -0.10923
      135B ->145B       0.71815
      135B ->146B       0.10011
      137B ->145B      -0.37796
      138B ->148B      -0.18123
      139B ->146B       0.12763
```

4PyBTM+BH_3@4Py

```
Excitation energies and oscillator strengths:

Excited State   1:  2.257-A     2.4585 eV  504.30 nm  f=0.0318  <S**2>=1.024
   145A ->146A       0.30999
   139B ->145B      -0.21525
   140B ->145B      -0.23389
   143B ->145B      -0.17927
   144B ->145B       0.81399
This state for optimization and/or second-order correction.
Total Energy, E(TD-HF/TD-DFT) =  -4239.89068804
Copying the excited state density for this state as the 1-particle RhoCI density.

Excited State   2:  2.202-A     2.6070 eV  475.58 nm  f=0.0067  <S**2>=0.963
   145A ->146A       0.25385
   145A ->147A       0.13088
   139B ->145B      -0.27564
   140B ->145B       0.15938
   142B ->145B      -0.54617
   143B ->145B       0.65909

Excited State   3:  2.135-A     2.6582 eV  466.43 nm  f=0.0122  <S**2>=0.890
   145A ->146A       0.11157
   140B ->145B      -0.19885
   142B ->145B       0.73228
   143B ->145B       0.59389

Excited State   4:  2.125-A     2.7361 eV  453.14 nm  f=0.0152  <S**2>=0.879
   145A ->147A       0.11184
   140B ->145B       0.59732
   141B ->145B       0.71887
   142B ->145B       0.21323
   143B ->145B      -0.12984

Excited State   5:  2.174-A     2.7507 eV  450.74 nm  f=0.0237  <S**2>=0.932
   145A ->146A      -0.28433
   145A ->147A      -0.12821
   139B ->145B       0.24936
   140B ->145B      -0.53921
   141B ->145B       0.62052
   142B ->145B      -0.27342
   143B ->145B       0.14254

Excited State   6:  2.209-A     2.9572 eV  419.26 nm  f=0.3678  <S**2>=0.970
   144A ->146A      -0.10065
   145A ->146A       0.74220
   145A ->150A      -0.10059
   139B ->145B       0.35923
   141B ->145B       0.16603
   143B ->145B      -0.19184
   144B ->145B      -0.36912
   144B ->146B       0.10613

Excited State   7:  2.356-A     3.0783 eV  402.77 nm  f=0.0338  <S**2>=1.138
   139A ->147A       0.10851
   144A ->146A      -0.13710
   145A ->146A      -0.19249
   145A ->147A      -0.25889
   145A ->150A       0.12216
   136B ->145B      -0.44978
   137B ->145B       0.14721
   138B ->145B      -0.21410
   139B ->145B       0.50906
   140B ->145B       0.28100
   143B ->145B       0.17708
   144B ->145B       0.24549
   144B ->146B       0.13471

Excited State   8:  2.306-A     3.1251 eV  396.73 nm  f=0.0174  <S**2>=1.080
   145A ->147A       0.10322
   136B ->145B       0.34834
   137B ->145B       0.63789
   137B ->147B      -0.11164
   138B ->145B      -0.53683
   139B ->145B       0.11030
```

```
Excited State   9:  2.460-A     3.3139 eV  374.13 nm  f=0.0471  <S**2>=1.263
   144A ->146A      -0.14690
   145A ->147A       0.44996
   133B ->145B       0.12405
   136B ->145B       0.39197
   137B ->145B      -0.20178
   138B ->145B       0.16828
   139B ->145B       0.51217
   144B ->145B       0.23155
   144B ->146B       0.18630

Excited State  10:  2.547-A     3.3597 eV  369.03 nm  f=0.1002  <S**2>=1.372
   141A ->148A       0.14503
   145A ->147A       0.60679
   145A ->150A       0.12010
   136B ->145B      -0.43013
   137B ->145B       0.11991
   138B ->145B      -0.14269
   140B ->145B      -0.29425
   140B ->147B       0.14037
   141B ->148B      -0.12045
   143B ->145B      -0.13004

Excited State  11:  2.986-A     3.4419 eV  360.22 nm  f=0.0205  <S**2>=1.979
   136A ->149A       0.12306
   143A ->146A       0.13996
   144A ->146A      -0.45596
   145A ->146A      -0.28169
   145A ->147A      -0.18586
   135B ->149B       0.12473
   136B ->145B       0.19607
   139B ->145B      -0.23156
   140B ->145B      -0.11795
   143B ->145B      -0.12785
   143B ->146B       0.15105
   144B ->145B      -0.23245
   144B ->146B       0.46428

Excited State  12:  2.180-A     3.6839 eV  336.56 nm  f=0.0002  <S**2>=0.938
   135B ->145B      -0.22240
   137B ->145B       0.62203
   138B ->145B       0.71696

Excited State  13:  3.051-A     3.7612 eV  329.64 nm  f=0.0103  <S**2>=2.077
   139A ->147A       0.10409
   140A ->147A      -0.14342
   141A ->148A       0.18889
   141A ->149A       0.20242
   141A ->151A       0.19364
   142A ->148A      -0.14454
   142A ->152A      -0.13882
   143A ->148A      -0.15304
   143A ->152A       0.14883
   145A ->146A       0.19040
   145A ->147A      -0.27093
   145A ->148A       0.14655
   145A ->150A       0.19565
   136B ->145B       0.34389
   140B ->147B       0.15852
   141B ->148B      -0.16971
   141B ->149B       0.18231
   141B ->150B       0.16997
   141B ->151B      -0.15610
   142B ->147B      -0.11216
   142B ->148B       0.10929
   142B ->152B       0.11687
   143B ->148B      -0.12526
   143B ->152B       0.13572

Excited State  14:  2.322-A     3.7809 eV  327.92 nm  f=0.0098  <S**2>=1.098
   145A ->148A       0.89101
   145A ->149A       0.19707
   135B ->145B       0.11483
   142B ->146B      -0.15122

Excited State  15:  3.183-A     3.7938 eV  326.81 nm  f=0.0013  <S**2>=2.283
   136A ->149A      -0.13183
```

139A	->146A	0.16696
140A	->146A	0.18336
141A	->148A	0.16782
142A	->148A	0.22833
142A	->151A	-0.21518
143A	->146A	-0.10885
143A	->152A	0.11474
144A	->150A	0.11455
145A	->150A	-0.22715
123B	->146B	-0.10901
135B	->145B	0.14788
135B	->149B	-0.12551
136B	->145B	0.25438
139B	->145B	0.20293
139B	->146B	-0.20743
140B	->146B	-0.10774
140B	->148B	-0.10209
140B	->151B	0.11342
141B	->148B	-0.12377
142B	->148B	-0.22511
142B	->150B	-0.12813
142B	->151B	0.19624
143B	->151B	-0.10650
144B	->152B	-0.11698

4PyBTM+BH_3@PyBTM

```
Excitation energies and oscillator strengths:

Excited State   1:  2.138-A     2.3501 eV  527.57 nm  f=0.0716  <S**2>=0.893
   145A ->146A      -0.11237
   145A ->147A       0.15658
   135B ->145B      -0.12339
   143B ->145B      -0.13410
   144B ->145B       0.93673
This state for optimization and/or second-order correction.
Total Energy, E(TD-HF/TD-DFT) =  -4239.88855907
Copying the excited state density for this state as the 1-particle RhoCI density.

Excited State   2:  2.115-A     2.4403 eV  508.07 nm  f=0.0077  <S**2>=0.869
   145A ->146A       0.15767
   140B ->145B      -0.15547
   142B ->145B       0.38890
   143B ->145B       0.85778
   144B ->145B       0.12279

Excited State   3:  2.155-A     2.5551 eV  485.24 nm  f=0.0265  <S**2>=0.911
   145A ->146A       0.25781
   138B ->145B       0.15030
   139B ->145B       0.17550
   140B ->145B      -0.50878
   141B ->145B       0.59919
   142B ->145B       0.27094
   143B ->145B      -0.33050

Excited State   4:  2.100-A     2.6262 eV  472.11 nm  f=0.0069  <S**2>=0.852
   145A ->146A      -0.19730
   140B ->145B       0.65020
   141B ->145B       0.67693
   142B ->145B       0.15985

Excited State   5:  2.201-A     2.8148 eV  440.47 nm  f=0.0400  <S**2>=0.961
   140A ->146A      -0.11457
   145A ->146A      -0.43245
   145A ->147A      -0.11533
   145A ->151A      -0.12631
   134B ->145B       0.16881
   138B ->145B       0.79982

Excited State   6:  2.209-A     2.8547 eV  434.32 nm  f=0.0162  <S**2>=0.970
   138A ->146A      -0.14190
   145A ->146A      -0.12541
   137B ->145B       0.94974
   137B ->146B      -0.11130

Excited State   7:  2.221-A     2.9579 eV  419.17 nm  f=0.1507  <S**2>=0.983
   145A ->146A      -0.55319
   137B ->145B      -0.10464
   138B ->145B      -0.28101
   140B ->145B      -0.13645
   141B ->145B      -0.21051
   142B ->145B       0.64653
   143B ->145B      -0.15141
   144B ->145B      -0.13340

Excited State   8:  2.205-A     2.9649 eV  418.17 nm  f=0.1343  <S**2>=0.966
   145A ->146A       0.49995
   138B ->145B       0.22148
   140B ->145B       0.39659
   141B ->145B      -0.30244
   142B ->145B       0.53795
   143B ->145B      -0.27977

Excited State   9:  2.111-A     3.0681 eV  404.11 nm  f=0.0020  <S**2>=0.864
   139B ->145B       0.96398
   140B ->145B       0.19808

Excited State  10:  2.627-A     3.2162 eV  385.50 nm  f=0.1845  <S**2>=1.475
   141A ->149A      -0.10126
   144A ->146A       0.16668
   144A ->151A      -0.10220
   145A ->147A       0.76090
```

```
   145A ->151A      -0.11841
   138B ->145B       0.15278
   138B ->147B       0.11827
   140B ->145B       0.12728
   141B ->145B      -0.10857
   144B ->145B      -0.20140
   144B ->146B       0.22332

Excited State  11:  2.118-A     3.4064 eV  363.97 nm  f=0.0028  <S**2>=0.872
   136B ->145B       0.98531
   136B ->146B      -0.12272

Excited State  12:  2.875-A     3.4807 eV  356.21 nm  f=0.0280  <S**2>=1.817
   138A ->148A       0.15151
   140A ->146A       0.14318
   141A ->149A       0.16211
   143A ->146A       0.10333
   144A ->147A      -0.21937
   145A ->147A      -0.17028
   145A ->151A       0.18323
   134B ->145B      -0.26855
   135B ->145B       0.47240
   137B ->148B      -0.13685
   138B ->145B       0.30535
   139B ->152B       0.10793
   140B ->145B       0.10806
   140B ->146B       0.14486
   141B ->149B      -0.14398
   143B ->146B      -0.10559
   144B ->146B       0.14525
   144B ->147B      -0.22766

Excited State  13:  2.621-A     3.5960 eV  344.78 nm  f=0.0025  <S**2>=1.467
   141A ->149A      -0.14315
   143A ->150A      -0.18536
   144A ->147A       0.11611
   145A ->146A      -0.11054
   135B ->145B       0.75540
   138B ->145B      -0.17365
   140B ->146B      -0.12036
   141B ->149B       0.13239
   143B ->150B      -0.18342
   144B ->145B       0.11789
   144B ->146B      -0.11159
   144B ->147B       0.11929

Excited State  14:  2.322-A     3.6305 eV  341.51 nm  f=0.0143  <S**2>=1.098
   144A ->146A       0.10074
   145A ->146A       0.10422
   145A ->147A      -0.16671
   145A ->148A       0.82325
   134B ->145B       0.32206

Excited State  15:  2.648-A     3.6504 eV  339.65 nm  f=0.0224  <S**2>=1.503
   138A ->148A      -0.16933
   139A ->152A      -0.11651
   141A ->149A       0.11420
   144A ->147A      -0.17147
   145A ->146A       0.17088
   145A ->148A      -0.47464
   134B ->145B       0.56919
   137B ->148B       0.17198
   138B ->146B      -0.10112
   139B ->152B       0.15360
   140B ->145B      -0.11252
   144B ->146B       0.12901
   144B ->147B      -0.17909
```

MeO3PyBTM+BH_3@MeO3Py

```
Excitation energies and oscillator strengths:

Excited State   1:  2.193-A     2.4775 eV  500.45 nm  f=0.0494  <S**2>=0.952
   153A ->154A        0.20436
   153A ->156A        0.12852
   144B ->153B       -0.11938
   147B ->153B        0.10539
   152B ->153B        0.91649
This state for optimization and/or second-order correction.
Total Energy, E(TD-HF/TD-DFT) =  -4354.40855840
Copying the excited state density for this state as the 1-particle RhoCI density.

Excited State   2:  2.133-A     2.6183 eV  473.53 nm  f=0.0035  <S**2>=0.888
   153A ->154A       -0.11471
   153A ->155A        0.10113
   144B ->153B        0.11599
   147B ->153B        0.22623
   149B ->153B       -0.11738
   150B ->153B       -0.14413
   151B ->153B        0.90812

Excited State   3:  2.148-A     2.6875 eV  461.33 nm  f=0.0130  <S**2>=0.904
   153A ->154A        0.12930
   147B ->153B       -0.13158
   150B ->153B        0.91426
   151B ->153B        0.24484

Excited State   4:  2.170-A     2.7408 eV  452.36 nm  f=0.0231  <S**2>=0.927
   153A ->155A        0.17076
   153A ->156A        0.12639
   147B ->153B        0.64482
   148B ->153B        0.23791
   149B ->153B       -0.52009
   150B ->153B        0.24570
   151B ->153B       -0.24546
   152B ->153B       -0.10875

Excited State   5:  2.127-A     2.8066 eV  441.77 nm  f=0.0062  <S**2>=0.881
   153A ->154A       -0.15269
   153A ->155A        0.10495
   147B ->153B        0.48868
   149B ->153B        0.80766

Excited State   6:  2.439-A     3.0860 eV  401.77 nm  f=0.1846  <S**2>=1.237
   152A ->154A        0.15351
   153A ->154A        0.65096
   153A ->156A        0.22338
   145B ->153B        0.41510
   146B ->153B       -0.27357
   149B ->153B        0.10793
   150B ->153B       -0.13377
   152B ->153B       -0.23143
   152B ->154B       -0.17563

Excited State   7:  2.310-A     3.1281 eV  396.36 nm  f=0.0581  <S**2>=1.084
   147A ->155A        0.12142
   150A ->158A       -0.10446
   153A ->154A       -0.31962
   153A ->155A        0.35441
   153A ->160A        0.14974
   143B ->153B        0.42128
   144B ->153B        0.35346
   145B ->153B        0.29880
   146B ->153B       -0.26961
   147B ->153B       -0.25590
   150B ->153B        0.10508
   150B ->158B        0.10338
   152B ->153B        0.14911

Excited State   8:  2.344-A     3.1666 eV  391.53 nm  f=0.0707  <S**2>=1.124
   152A ->154A       -0.10521
   153A ->154A       -0.28687
   143B ->153B       -0.19783
   144B ->153B       -0.52560
   145B ->153B        0.60272
```

```
   145B ->155B       0.10294
   146B ->153B      -0.24410

Excited State   9:  2.114-A     3.3240 eV  373.00 nm  f=0.0031  <S**2>=0.867
   153A ->156A       0.13096
   147B ->153B      -0.28207
   148B ->153B       0.92313
   149B ->153B       0.10313

Excited State  10:  2.439-A     3.3466 eV  370.48 nm  f=0.1567  <S**2>=1.237
   149A ->157A       0.16763
   149A ->158A      -0.11373
   152A ->156A      -0.11043
   153A ->155A       0.59595
   153A ->156A       0.45021
   153A ->160A      -0.11153
   143B ->153B      -0.16248
   144B ->153B      -0.22904
   145B ->153B      -0.15382
   146B ->153B       0.17340
   147B ->153B      -0.21249
   147B ->156B       0.12120
   148B ->153B      -0.20457
   149B ->157B      -0.13919
   149B ->158B       0.10046
   152B ->156B       0.10546

Excited State  11:  2.755-A     3.5019 eV  354.05 nm  f=0.0261  <S**2>=1.648
   144A ->154A       0.13230
   147A ->154A       0.11362
   148A ->154A       0.10142
   152A ->154A      -0.17830
   152A ->160A      -0.11176
   153A ->155A      -0.18658
   153A ->156A       0.16594
   142B ->153B      -0.13458
   143B ->153B       0.56285
   144B ->153B      -0.21625
   144B ->154B       0.12081
   146B ->153B       0.28582
   148B ->154B      -0.12448
   148B ->155B       0.10812
   150B ->154B       0.10258
   152B ->153B      -0.10260
   152B ->154B       0.21151
   152B ->160B      -0.11763

Excited State  12:  2.850-A     3.5844 eV  345.90 nm  f=0.0237  <S**2>=1.780
   148A ->156A      -0.10612
   149A ->157A       0.11293
   150A ->154A      -0.10314
   151A ->158A       0.10820
   151A ->159A      -0.16258
   152A ->154A      -0.27667
   152A ->156A      -0.11981
   153A ->154A       0.28959
   153A ->155A      -0.14010
   153A ->160A      -0.10087
   144B ->153B       0.39111
   146B ->153B      -0.34238
   147B ->153B      -0.14778
   148B ->154B      -0.10030
   149B ->157B      -0.10604
   150B ->154B       0.10553
   151B ->159B       0.16557
   152B ->153B       0.12938
   152B ->154B       0.30169
   152B ->156B       0.11327

Excited State  13:  2.614-A     3.6377 eV  340.83 nm  f=0.0014  <S**2>=1.459
   147A ->156A      -0.10539
   152A ->155A      -0.15420
   152A ->156A       0.13313
   153A ->154A      -0.35688
   153A ->155A      -0.47166
   153A ->156A       0.54617
   144B ->153B       0.15156
```

```
  146B ->153B      -0.19912
  147B ->156B       0.11797
  152B ->155B      -0.17799

Excited State  14:  2.250-A     3.7097 eV  334.22 nm  f=0.0016  <S**2>=1.015
  153A ->155A      -0.10649
  144B ->153B       0.37837
  145B ->153B       0.49662
  146B ->153B       0.69007

Excited State  15:  2.279-A     3.7727 eV  328.63 nm  f=0.0105  <S**2>=1.048
  153A ->157A       0.94055
```

MeO3PyBTM+BH_3@PyBTM

```
Excitation energies and oscillator strengths:

Excited State   1:  2.090-A     2.0181 eV  614.38 nm  f=0.1139  <S**2>=0.842
   153A ->155A      -0.10160
   150B ->153B      -0.19910
   152B ->153B       0.95130
This state for optimization and/or second-order correction.
Total Energy, E(TD-HF/TD-DFT) =  -4354.43755000
Copying the excited state density for this state as the 1-particle RhoCI density.

Excited State   2:  2.082-A     2.3995 eV  516.70 nm  f=0.0035  <S**2>=0.833
   149B ->153B       0.11344
   151B ->153B       0.97296

Excited State   3:  2.163-A     2.5512 eV  485.98 nm  f=0.0297  <S**2>=0.919
   153A ->154A      -0.26502
   153A ->155A       0.10381
   145B ->153B       0.11803
   148B ->153B      -0.28235
   149B ->153B       0.74447
   150B ->153B       0.39496
   151B ->153B      -0.16252

Excited State   4:  2.131-A     2.6117 eV  474.72 nm  f=0.0047  <S**2>=0.885
   153A ->154A       0.25940
   147B ->153B      -0.10443
   149B ->153B      -0.33909
   150B ->153B       0.83691
   152B ->153B       0.19817

Excited State   5:  2.163-A     2.6861 eV  461.57 nm  f=0.0076  <S**2>=0.920
   153A ->154A       0.42613
   147B ->153B      -0.12502
   148B ->153B       0.69467
   149B ->153B       0.46620
   152B ->153B       0.12655

Excited State   6:  2.224-A     2.8751 eV  431.23 nm  f=0.0781  <S**2>=0.987
   146A ->154A      -0.11835
   153A ->154A       0.40686
   153A ->155A       0.13739
   146B ->153B       0.80260
   148B ->153B      -0.29146

Excited State   7:  2.119-A     2.9073 eV  426.46 nm  f=0.1241  <S**2>=0.872
   153A ->154A       0.49112
   143B ->153B      -0.13349
   145B ->153B       0.40291
   146B ->153B      -0.43045
   147B ->153B      -0.33444
   148B ->153B      -0.41794
   150B ->153B      -0.15643
   152B ->153B      -0.10469

Excited State   8:  2.524-A     2.9365 eV  422.22 nm  f=0.1105  <S**2>=1.343
   148A ->154A      -0.12575
   152A ->155A       0.12455
   153A ->154A       0.34064
   153A ->155A       0.27338
   153A ->159A      -0.10977
   142B ->153B      -0.15444
   145B ->153B      -0.41463
   146B ->153B      -0.28761
   147B ->153B       0.47161
   148B ->153B      -0.23376
   148B ->154B       0.10844
   149B ->153B       0.10298
   152B ->153B       0.11484
   152B ->154B      -0.14173
   152B ->155B      -0.14087

Excited State   9:  2.711-A     3.1359 eV  395.37 nm  f=0.1287  <S**2>=1.588
   152A ->154A       0.21599
   152A ->159A      -0.14607
   153A ->154A      -0.22897
```

```
   153A ->155A       0.62955
   145B ->153B       0.19123
   147B ->153B      -0.29002
   149B ->153B      -0.20048
   150B ->153B      -0.13265
   152B ->154B      -0.26569
   152B ->155B      -0.21603

Excited State  10:  2.089-A     3.3001 eV  375.69 nm  f=0.0004  <S**2>=0.841
   145B ->153B       0.68798
   147B ->153B       0.68903
   148B ->153B       0.13663

Excited State  11:  2.926-A     3.4333 eV  361.12 nm  f=0.1213  <S**2>=1.890
   148A ->157A      -0.10615
   149A ->157A      -0.12879
   150A ->157A       0.13499
   152A ->154A      -0.17622
   152A ->155A      -0.35726
   153A ->155A       0.38612
   153A ->159A       0.23147
   142B ->153B       0.12276
   143B ->153B       0.15550
   145B ->153B      -0.16237
   146B ->153B      -0.12144
   147B ->153B       0.11767
   148B ->153B      -0.11987
   148B ->157B       0.10298
   149B ->155B      -0.12092
   149B ->157B       0.11457
   150B ->153B      -0.13099
   150B ->157B      -0.11411
   152B ->154B       0.27505
   152B ->155B       0.31031
   152B ->159B      -0.10141

Excited State  12:  2.125-A     3.4537 eV  358.99 nm  f=0.0056  <S**2>=0.879
   144B ->153B       0.98265
   144B ->154B      -0.12660

Excited State  13:  2.210-A     3.5593 eV  348.34 nm  f=0.0051  <S**2>=0.971
   152A ->156A      -0.18542
   153A ->155A      -0.16444
   153A ->156A       0.91295

Excited State  14:  2.588-A     3.5973 eV  344.66 nm  f=0.0260  <S**2>=1.424
   146A ->156A       0.20990
   148A ->154A       0.13224
   152A ->155A       0.17331
   153A ->154A      -0.13629
   153A ->156A      -0.15335
   142B ->153B       0.55192
   143B ->153B       0.44045
   146B ->156B      -0.20834
   147B ->153B       0.14724
   148B ->153B      -0.18491
   149B ->153B       0.12805
   152B ->154B      -0.16803
   152B ->155B      -0.18496

Excited State  15:  2.666-A     3.6681 eV  338.01 nm  f=0.0082  <S**2>=1.527
   148A ->154A      -0.13587
   151A ->158A       0.22160
   142B ->153B      -0.13427
   143B ->153B       0.69849
   145B ->153B       0.24852
   147B ->153B      -0.10333
   148B ->154B       0.14053
   150B ->155B       0.10193
   151B ->158B      -0.19795
```

IV. NMR spectra

・**αH-4PyPyBTM:**

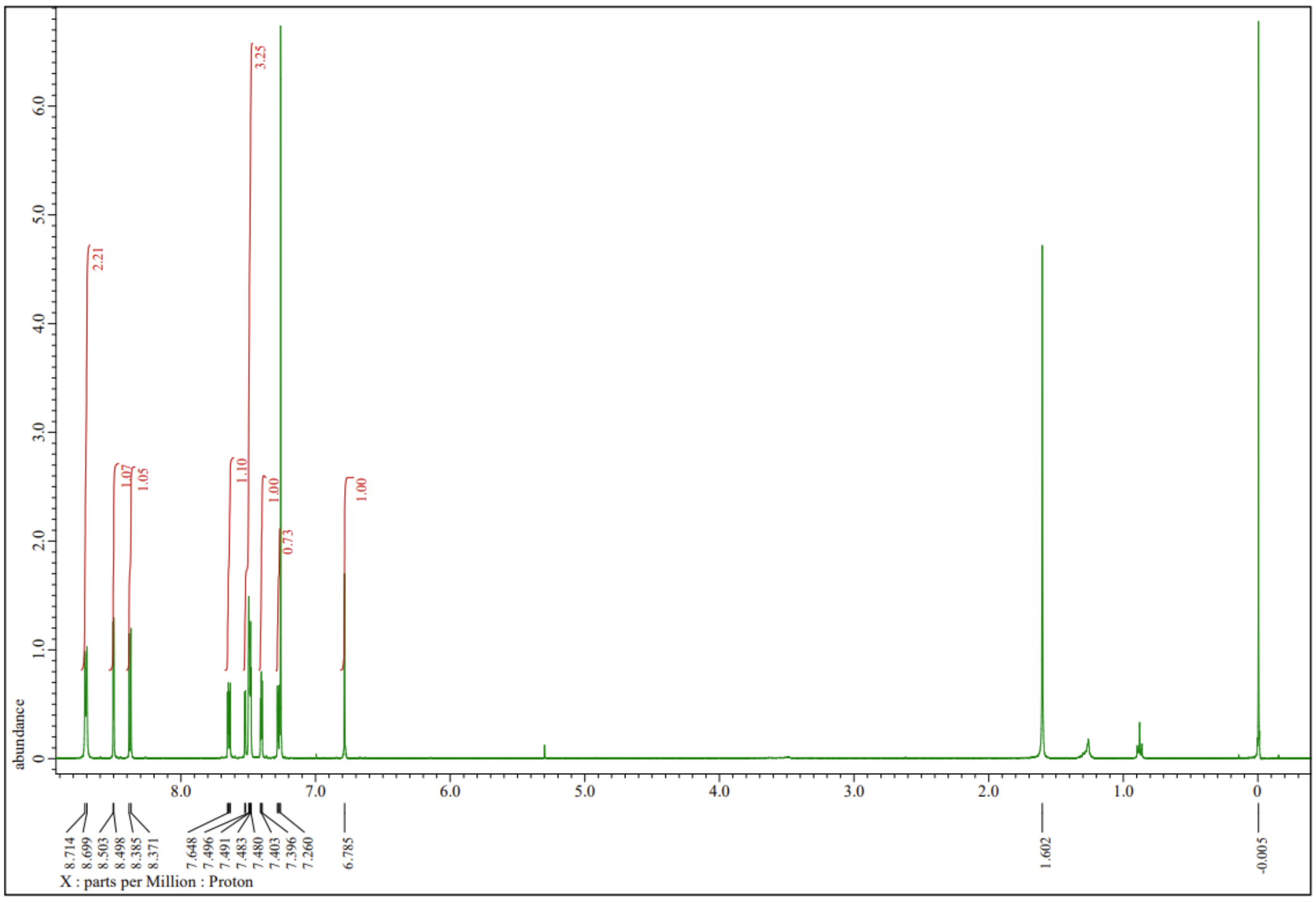

^{1}H-NMR (400 MHz, $CDCl_3$, ppm) δ 8.71 (d, J = 6.0 Hz, 2H), 8.50 (d, J = 1.8 Hz, 1H), 8.38 (d, J = 5.5 Hz, 1H), 7.64 (dd, J = 5.5, 1.8 Hz, 1H), 7.53-7.48 (m, 3H), 7.40 (t, J = 2.7 Hz, 1H), 7.28 (t, J = 3.0 Hz, 1H), 6.78 (s, 1H)

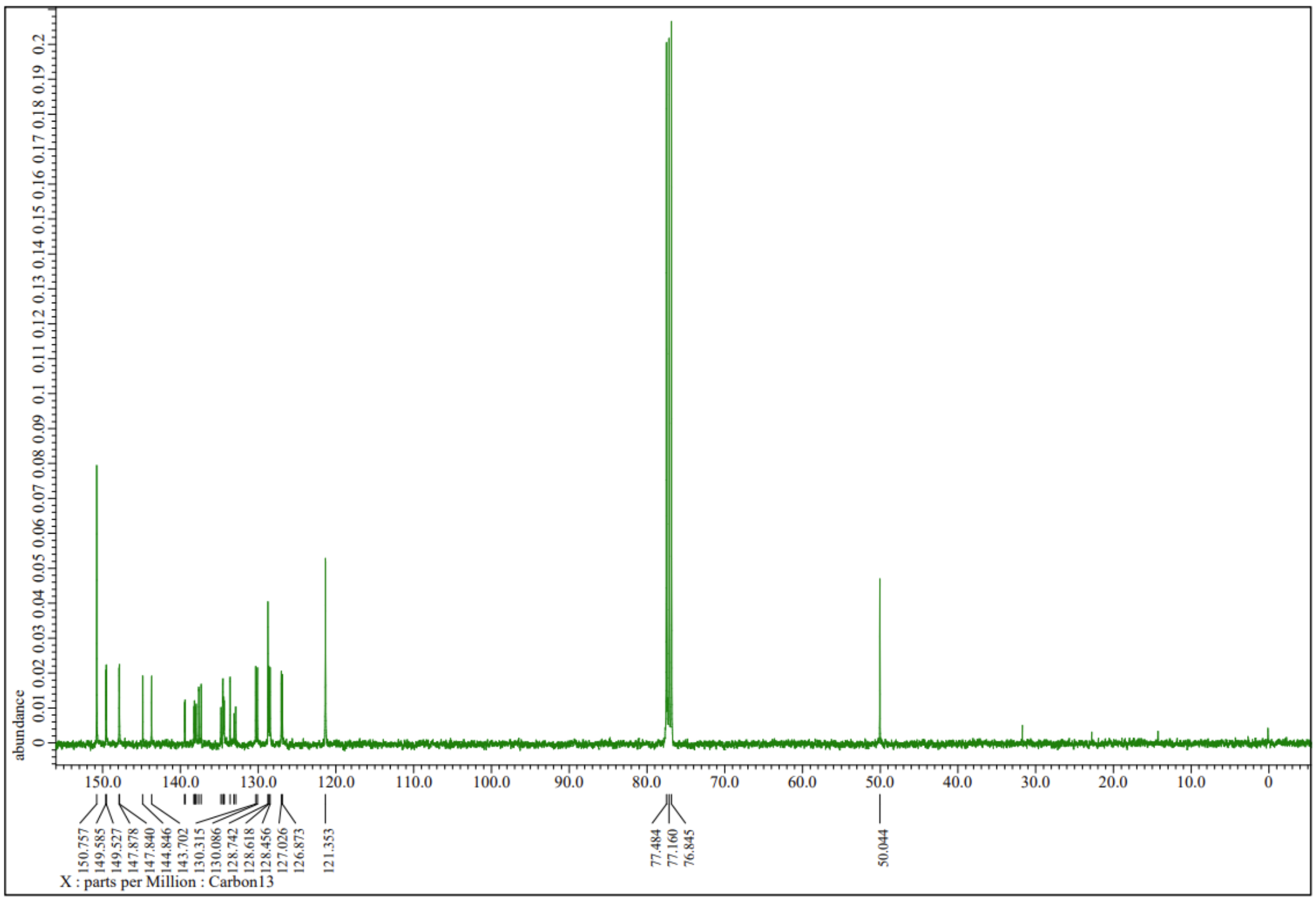

^{13}C-NMR (101 MHz, $CDCl_3$, ppm): δ 150.7, 150.6, 149.6, 149.5, 147.8, 147.8, 144.8, 143.7, 139.4, 139.4, 138.2, 138.2, 138.0, 137.9, 137.6, 137.6, 137.3, 134.8, 134.5, 134.5, 134.4, 134.3, 133.6, 133.1, 132.9, 130.3, 130.1, 128.7, 128.6, 128.5, 128.4, 127.0, 126.8, 121.3, 50.0.

・αH-4Py$_2$PyBTM:

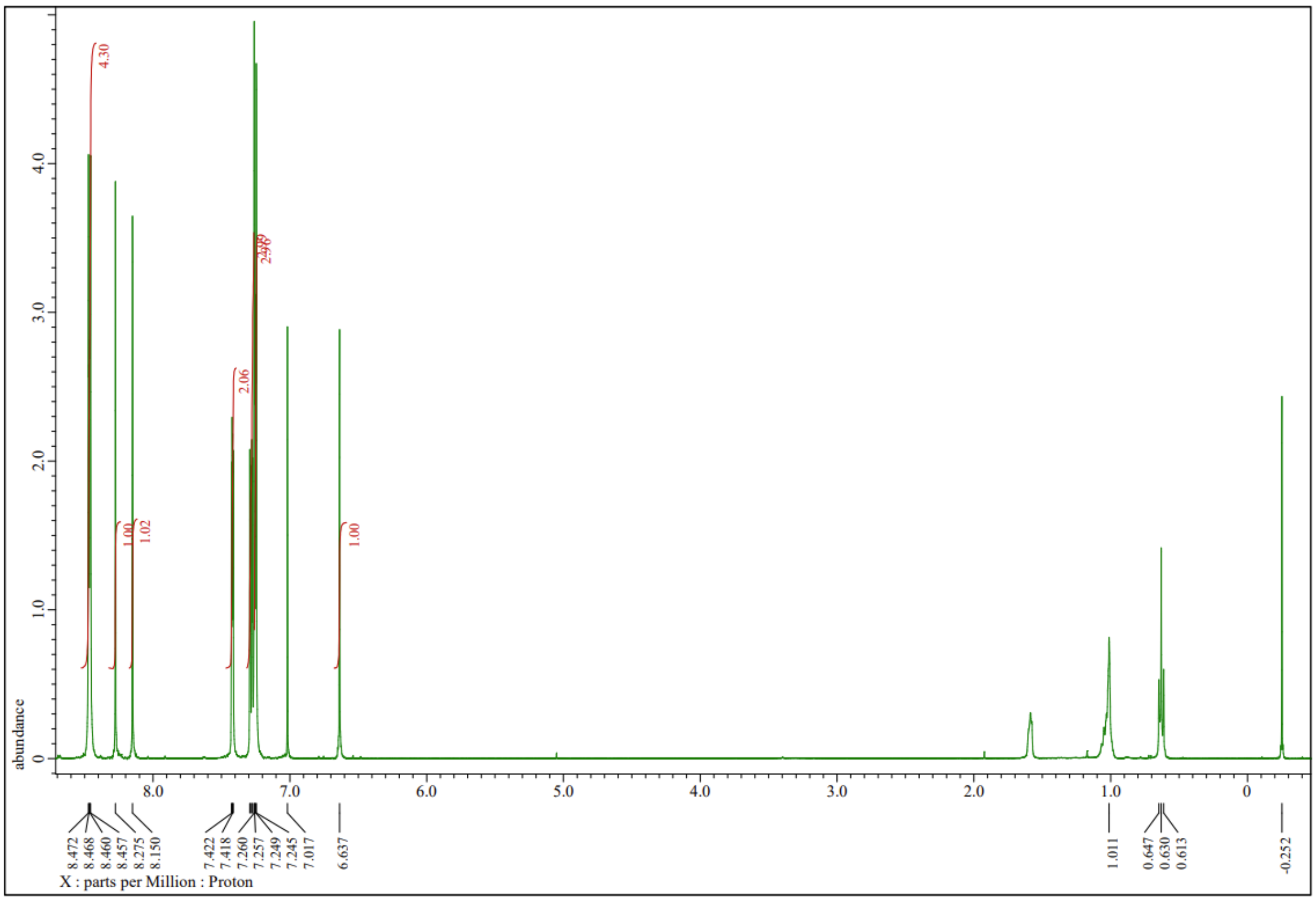

^{1}H-NMR (400 MHz, $CDCl_3$, ppm) δ 8.46 (dd, *J* = 4.6, 1.4 Hz, 4H), 8.27 (s, 1H), 8.15 (s, 1H), 7.42 (dd, *J* = 3.2, 1.6 Hz, 2H), 7.28 (dd, *J* = 6.0, 1.8 Hz, 3H), 7.26-7.25 (m, 3H), 6.64 (s, 1H)

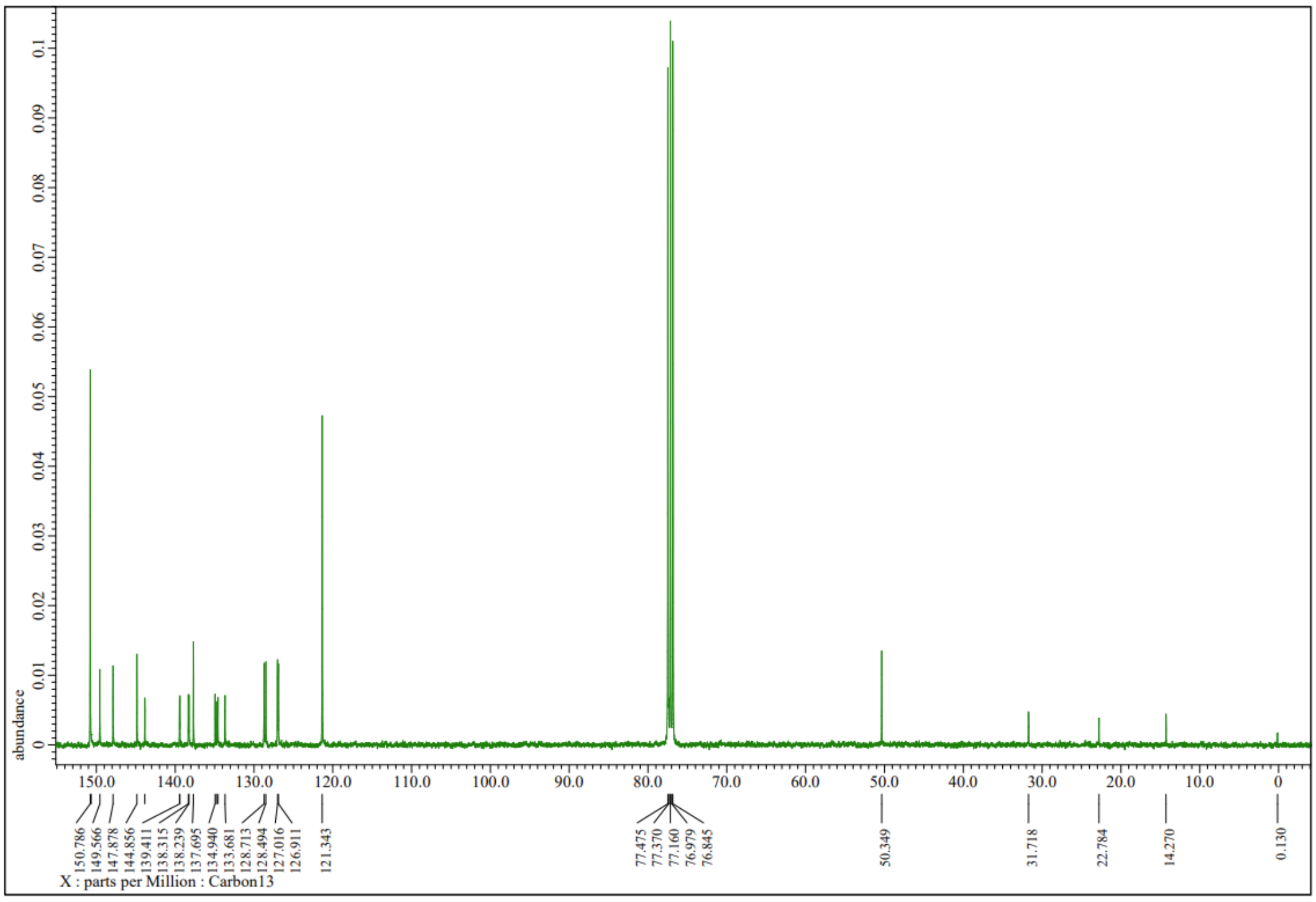

^{13}C-NMR (101 MHz, $CDCl_3$, ppm) : δ 150.8, 150.7, 149.6, 147.9, 144.9, 143.8, 139.5, 139.4, 138.3, 138.2, 137.7, 134.9, 134.7, 134.6, 133.7, 128.7, 128.5, 127.0, 126.9, 121.3, 50.3.

・**αH-3PyPyBTM:**

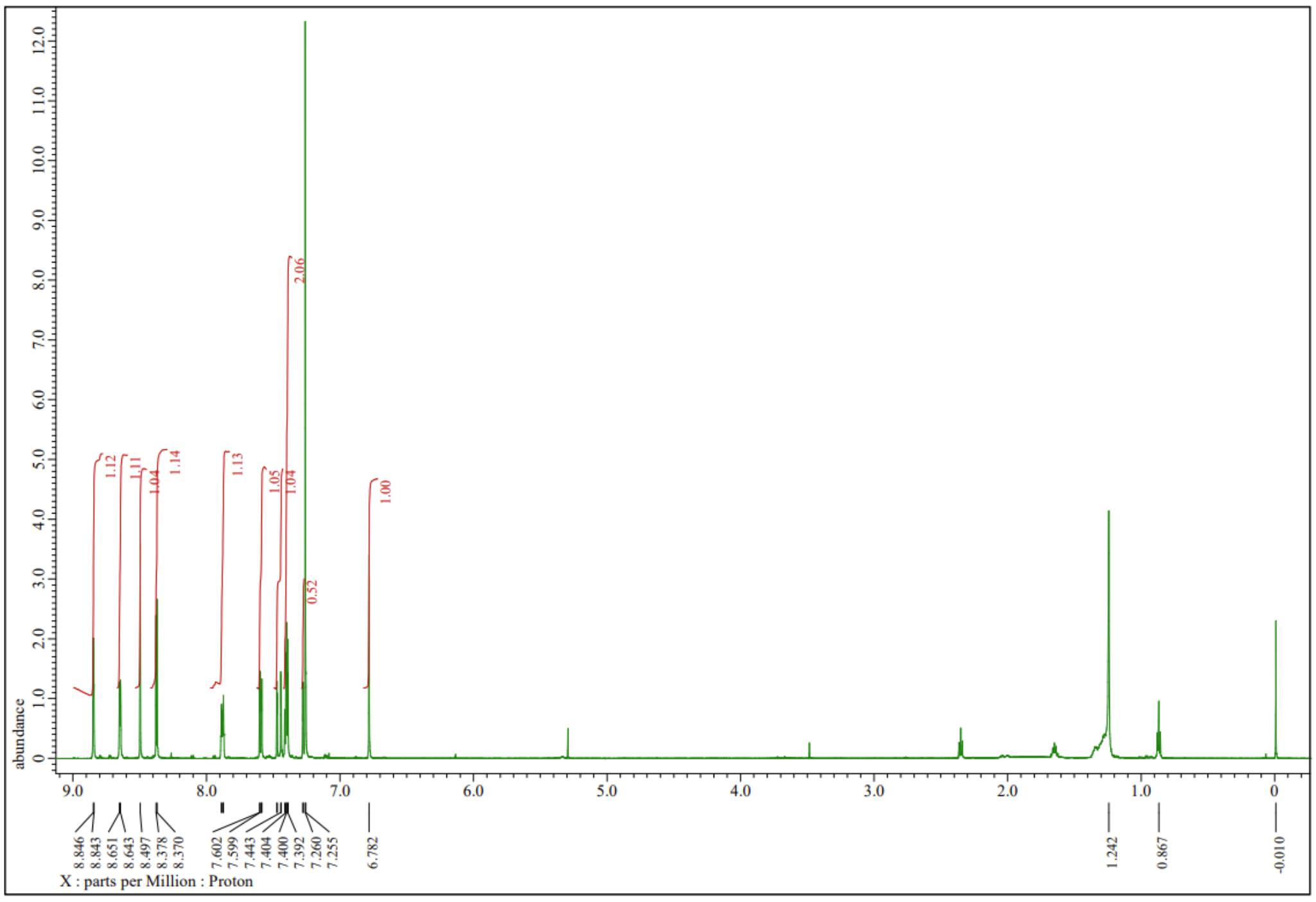

^{1}H-NMR (400 MHz, $CDCl_3$, ppm) δ 8.84 (d, J = 2.1 Hz, 1H), 8.65 (d, J = 4.8 Hz, 1H), 8.50 (s, 1H), 8.37 (d, J = 4.8 Hz, 1H), 7.88 (dd, J = 7.9, 1.7 Hz, 1H), 7.59 (dd, J = 8.2, 2.1 Hz, 1H), 7.46 (dd, J = 18.2, 1.7 Hz, 1H), 7.41-7.39 (m, 2H), 7.28 (d, J = 2.1 Hz, 1H), 6.78 (s, 1H).

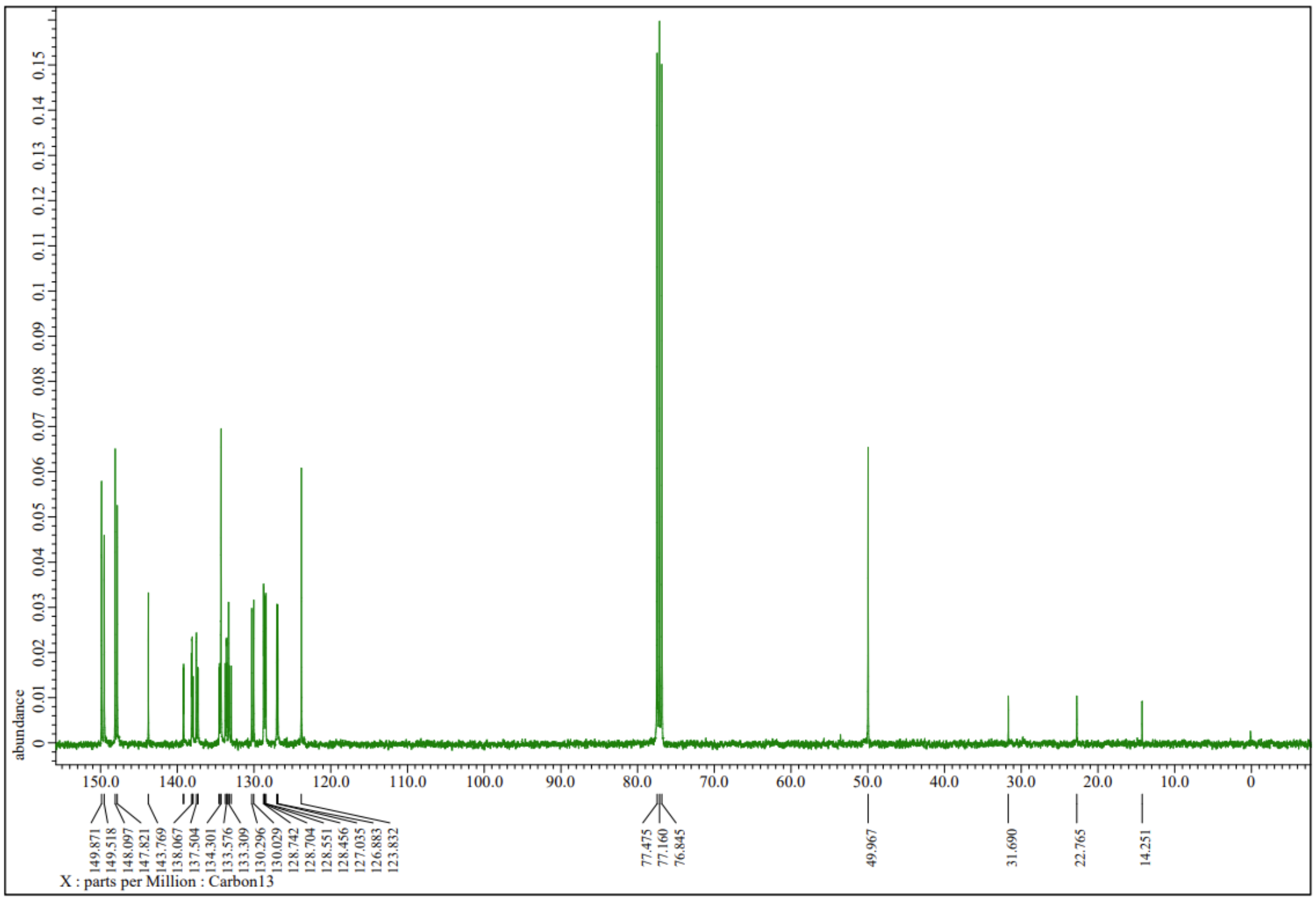

^{13}C-NMR (101 MHz, $CDCl_3$, ppm) : δ 149.9, 149.5, 148.1, 147.8, 143.8, 139.2, 139.2, 138.1, 138.1, 138.1, 137.9, 137.5, 137.3, 137.3, 134.5, 134.5, 134.3, 134.3, 133.7, 133.6, 133.6, 133.5, 133.3, 133.2, 133.0, 130.3, 130.0, 128.7, 128.7, 128.5, 128.5, 127.0, 126.9, 123.8, 50.0.

・αH-3Py$_2$PyBTM:

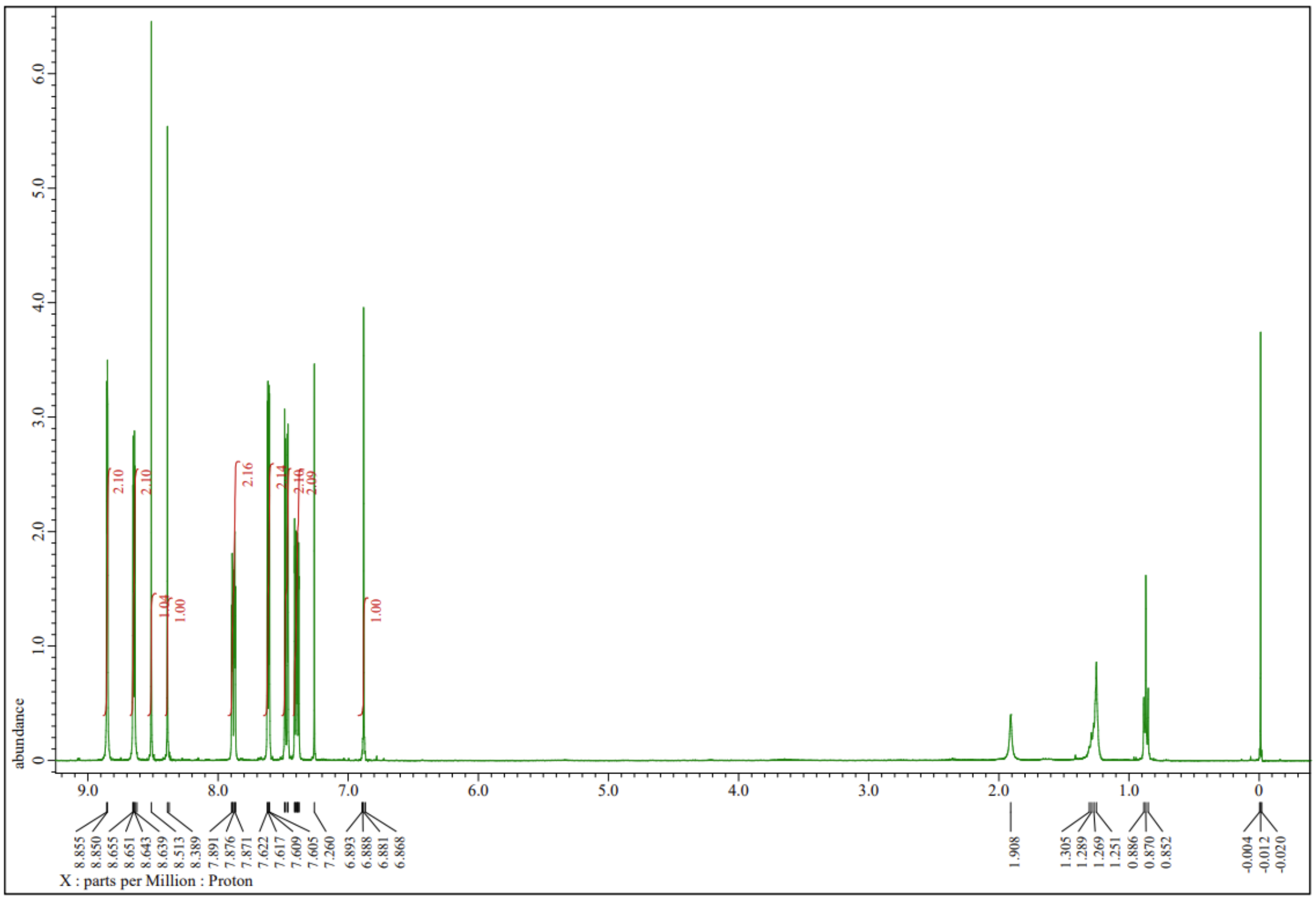

^{1}H-NMR (400 MHz, $CDCl_3$, ppm) δ 8.85 (d, *J* = 2.0 Hz, 2H), 8.65 (dd, *J* = 4.6, 1.4 Hz, 2H), 8.51 (s, 1H), 8.39 (s, 1H), 7.88 (dt, *J* = 8.2, 2.1 Hz, 2H), 7.61 (dd, *J* = 5.2, 2.0 Hz, 2H), 7.48 (dd, *J* = 8.8, 2.0 Hz, 2H), 7.41-7.38 (m, 2H), 6.88 (s, 1H).

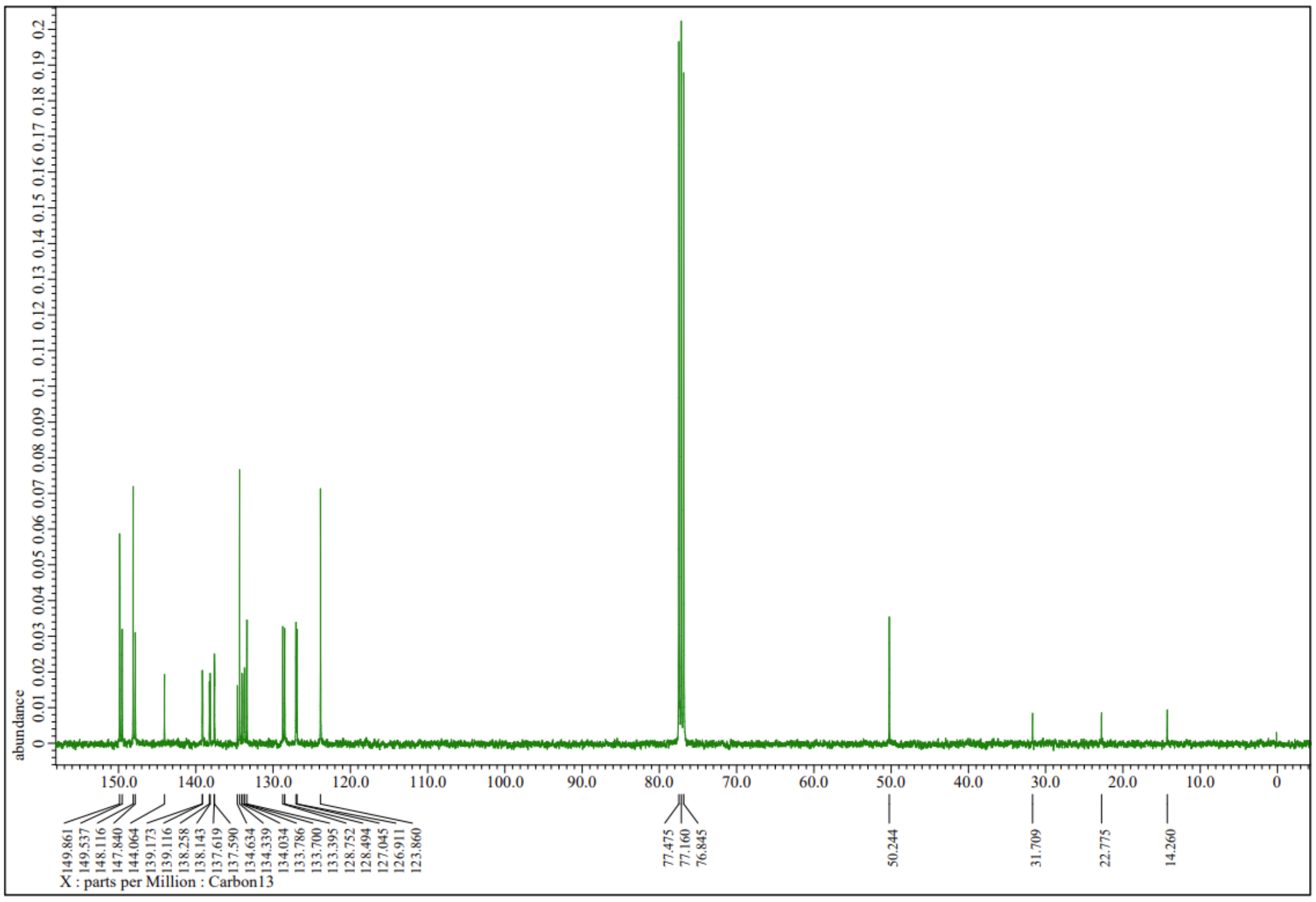

^{13}C-NMR (101 MHz, $CDCl_3$, ppm) : δ 149.9, 149.5, 148.1, 148.0, 147.8, 144.1, 139.2, 139.1, 138.3, 138.1, 137.6, 137.6, 134.6, 134.3, 134.0, 133.8, 133.7, 133.4, 128.8, 128.5, 128.4, 127.0, 126.9, 123.9, 50.2.

• **αH-MeO3PyPyBTM:**

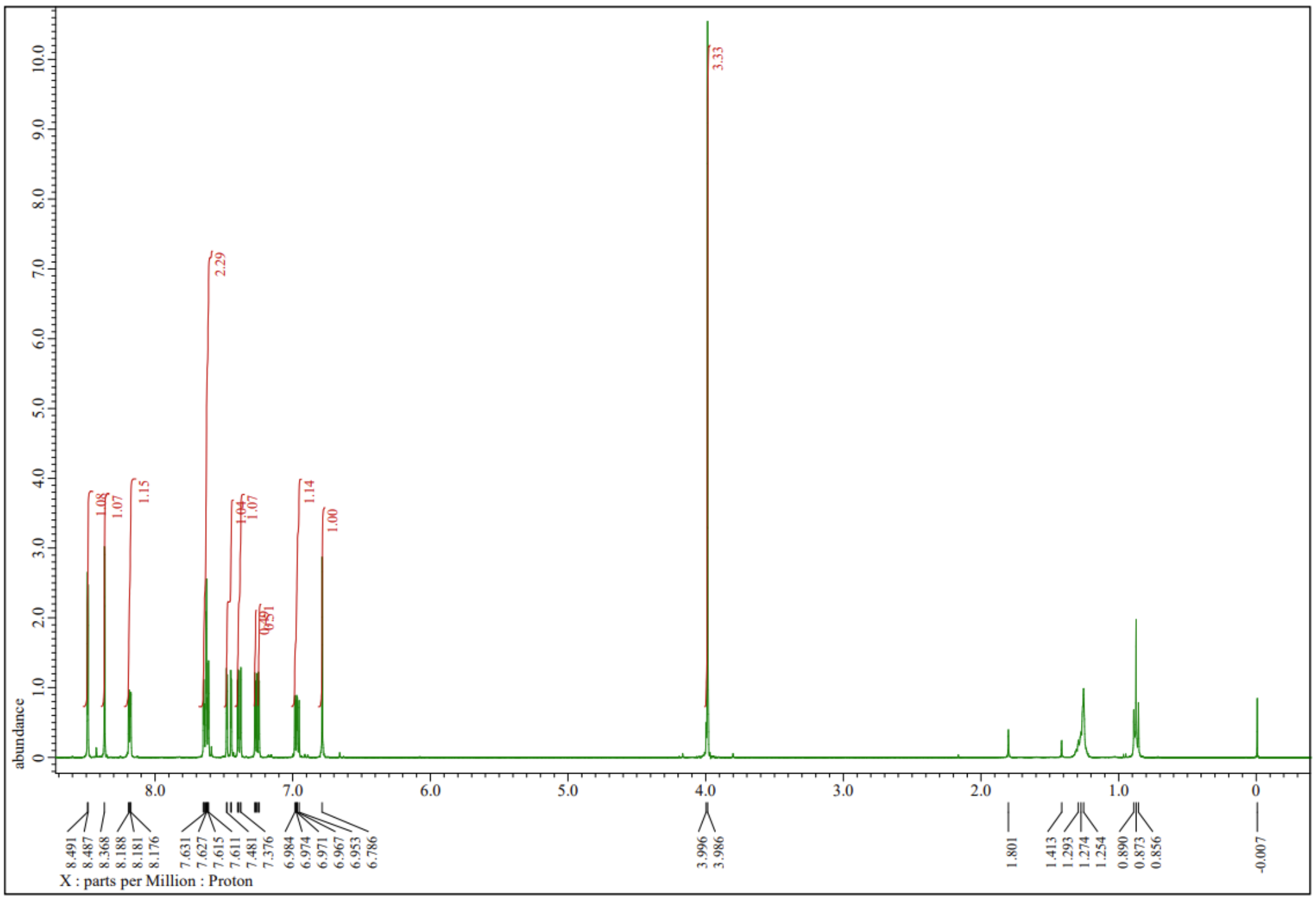

^{1}H-NMR (400 MHz, $CDCl_3$, ppm) δ 8.49 (d, J = 1.8 Hz, 1H), 8.37 (d, J = 0.9 Hz, 1H), 8.18 (dd, J = 5.0, 1.4 Hz, 1H), 7.65-7.61 (m, 2H), 7.46 (dd, J = 12.8, 1.8 Hz, 1H), 7.39 (dd, J = 7.6, 2.1 Hz, 1H), 7.25 (d, J = 2.3 Hz, 1H), 6.98-6.95 (m, 1H), 6.78 (s, 1H), 3.99 (d, J = 4.1 Hz, 3H).

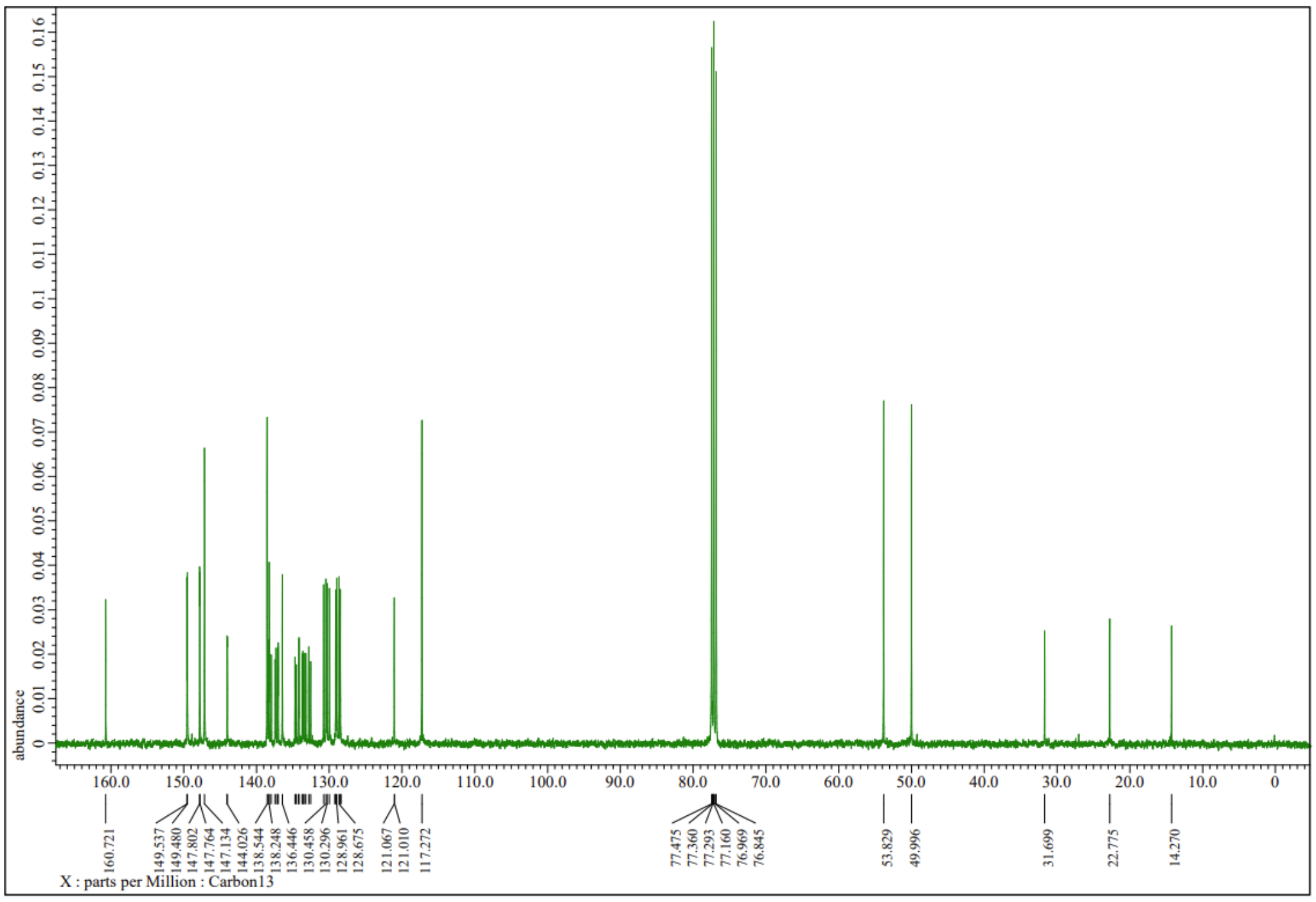

^{13}C-NMR (101 MHz, $CDCl_3$, ppm) δ 160.7, 149.5, 149.5, 147.8, 147.7, 147.1, 144.0, 144.0, 138.5, 138.3, 138.2, 137.9, 137.4, 137.3, 137.0, 137.0, 136.4, 134.7, 134.5, 134.2, 134.1, 133.7, 133.6, 133.4, 133.2, 132.8, 132.5, 130.7, 130.4, 130.3, 130.0, 129.2, 129.1, 128.9, 128.7, 128.5, 128.4, 121.1, 121.0, 117.3, 53.8, 50.0.

・**αH-MeO3Py₂PyBTM:**

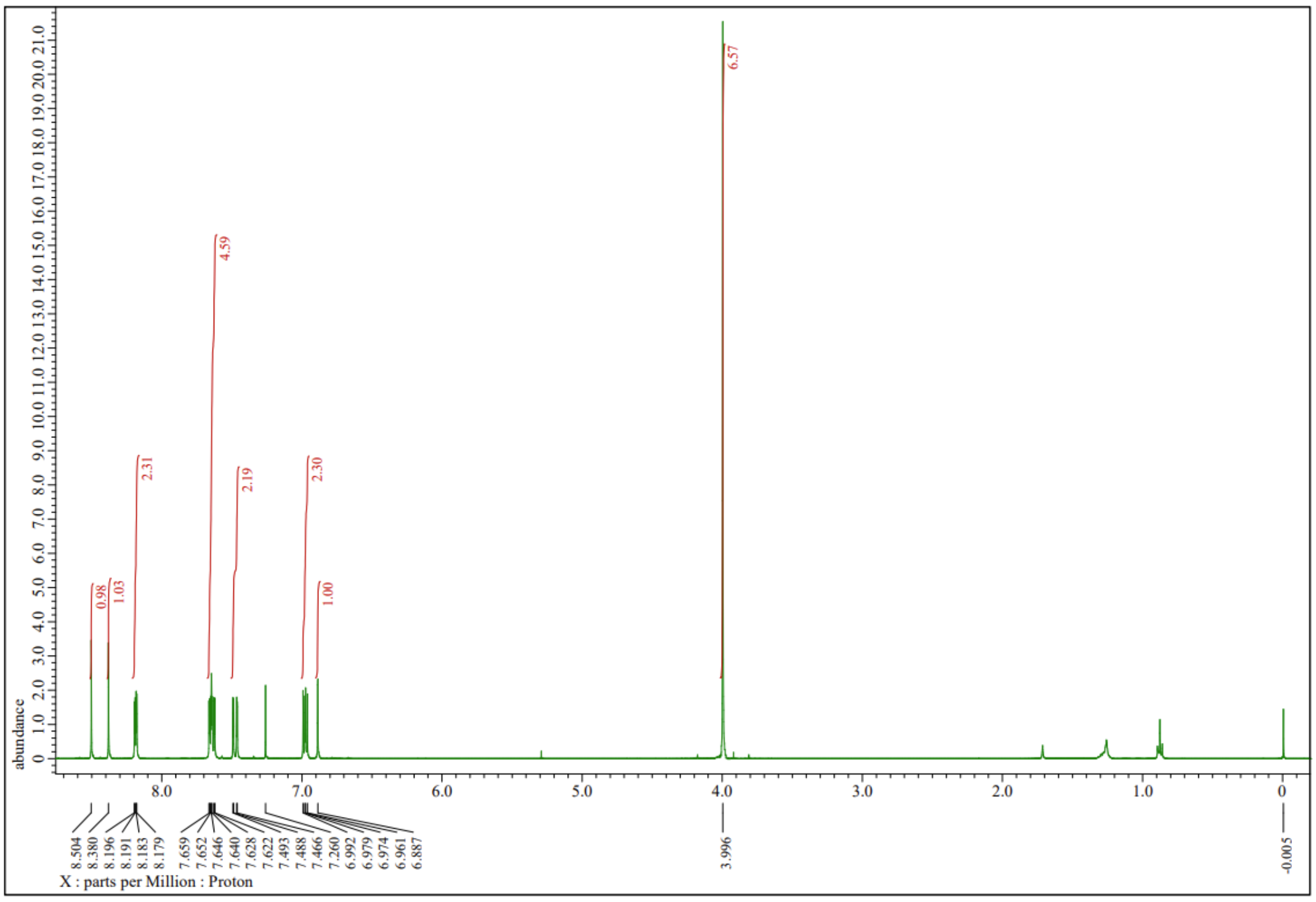

^{1}H-NMR (400 MHz, $CDCl_3$, ppm) δ 8.51 (s, 1H), 8.38 (s, 1H), 8.19 (dd *J* = 5.2, 2.0 Hz, 2H), 7.67-7.62 (m, 4H), 7.48 (dd, *J* = 10.8, 2.1 Hz, 2H), 6.99 (dd, *J* = 7.3, 4.8 Hz, 2H), 6.89 (s, 1H), 4.00 (s, 6H).

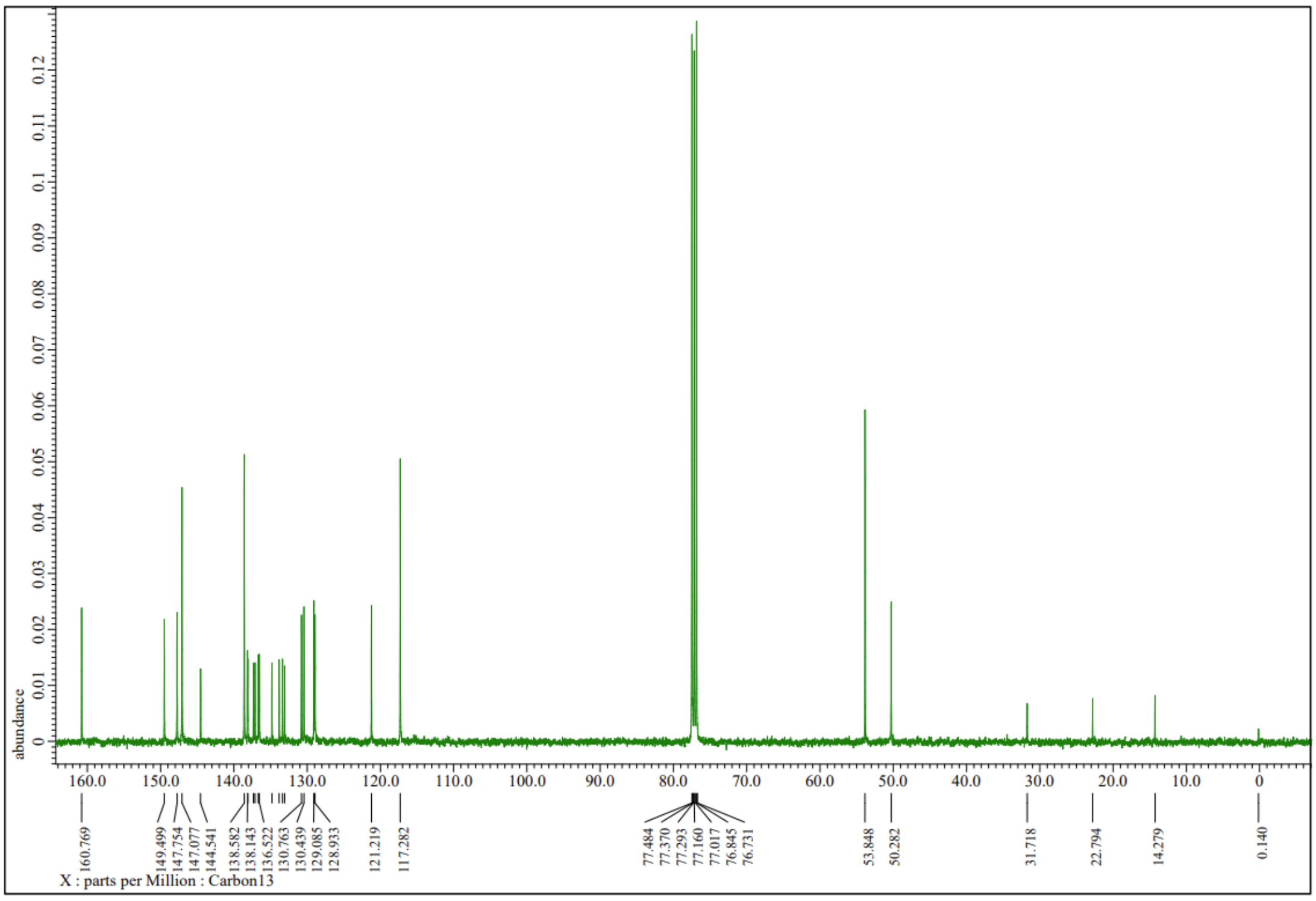

^{13}C-NMR (101 MHz, $CDCl_3$, ppm) δ 160.7, 149.5, 147.7, 147.0, 144.5, 138.5, 138.1, 138.0, 137.3, 137.1, 136.6, 136.5, 134.8, 133.8, 133.3, 133.0, 130.7, 130.4, 129.1, 129.0, 128.9, 121.2, 117.2, 53.8.

V. HR-MS spectra

HR-MALDI-TOF mass spectrum of **aH-4PyPyBTM**, full spectrum (bottom) and calculated isotopic pattern (center) and experimental isotopic pattern (top) of the molecular peak.

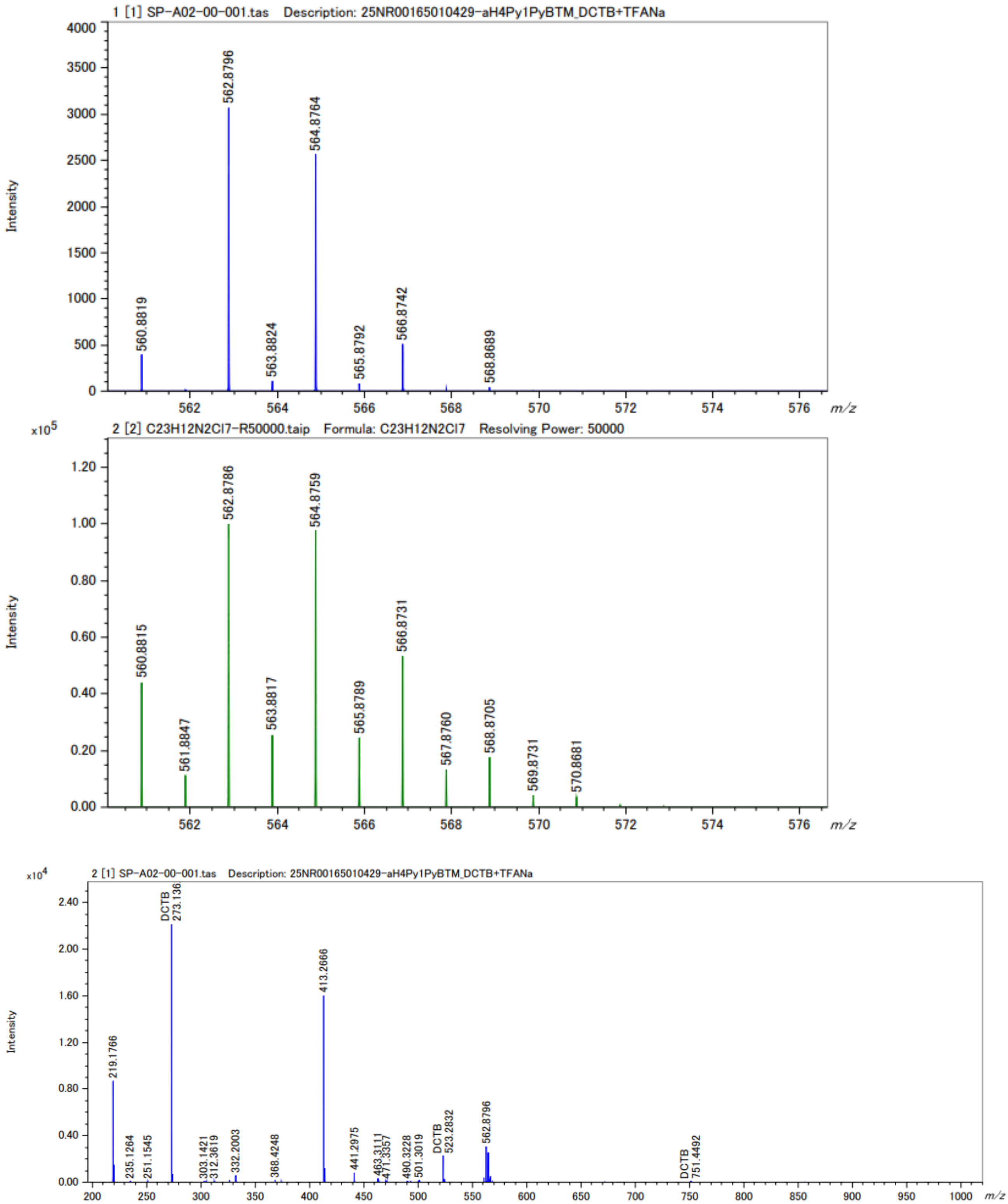

HR-MALDI-TOF mass spectrum of **aH-4Py$_2$PyBTM**, full spectrum (bottom) and calculated isotopic pattern (center) and experimental isotopic pattern (top) of the molecular peak.

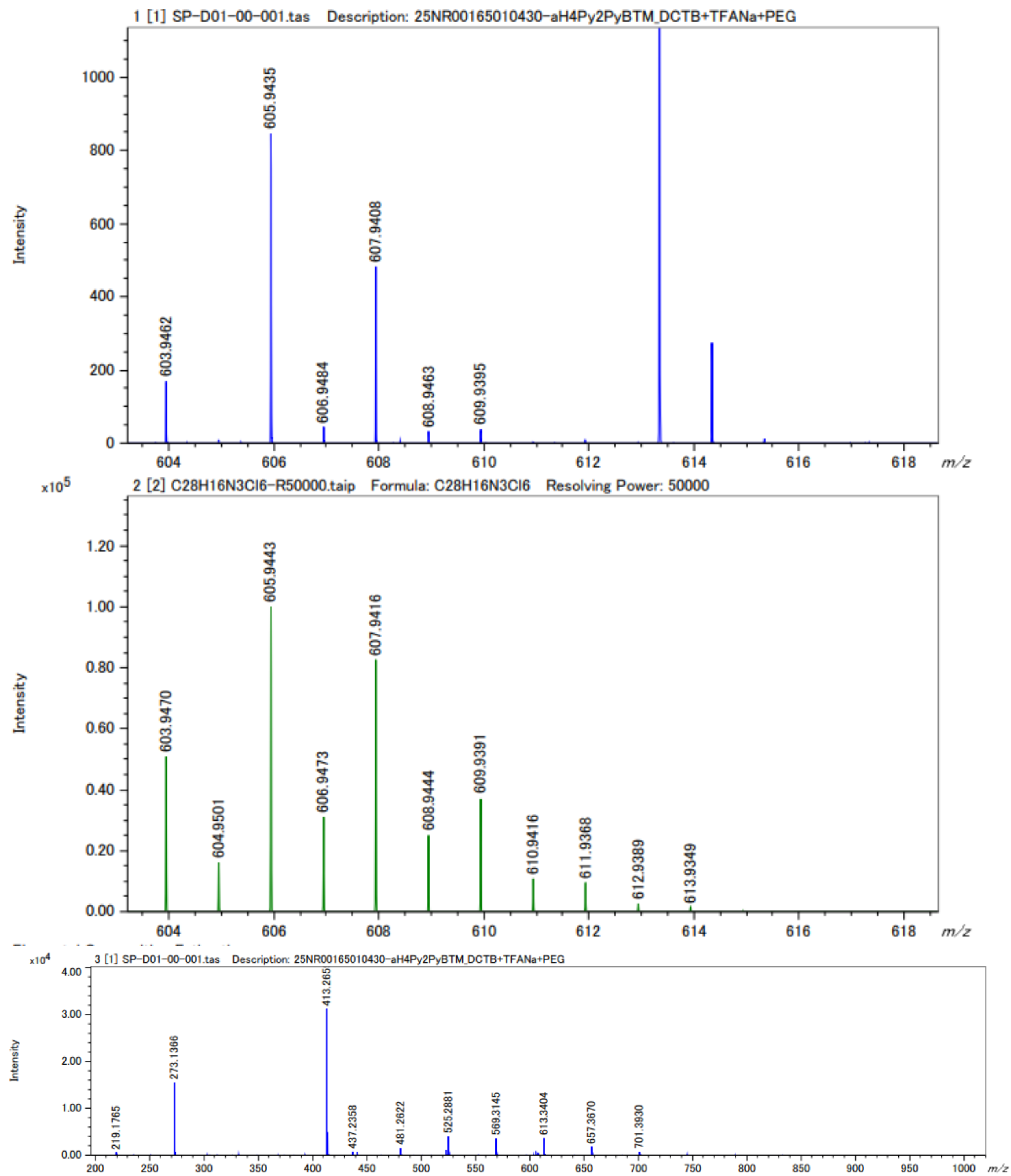

HR-MALDI-TOF mass spectrum of **aH-3PyPyBTM**, full spectrum (bottom) and calculated isotopic pattern (center) and experimental isotopic pattern (top) of the molecular peak.

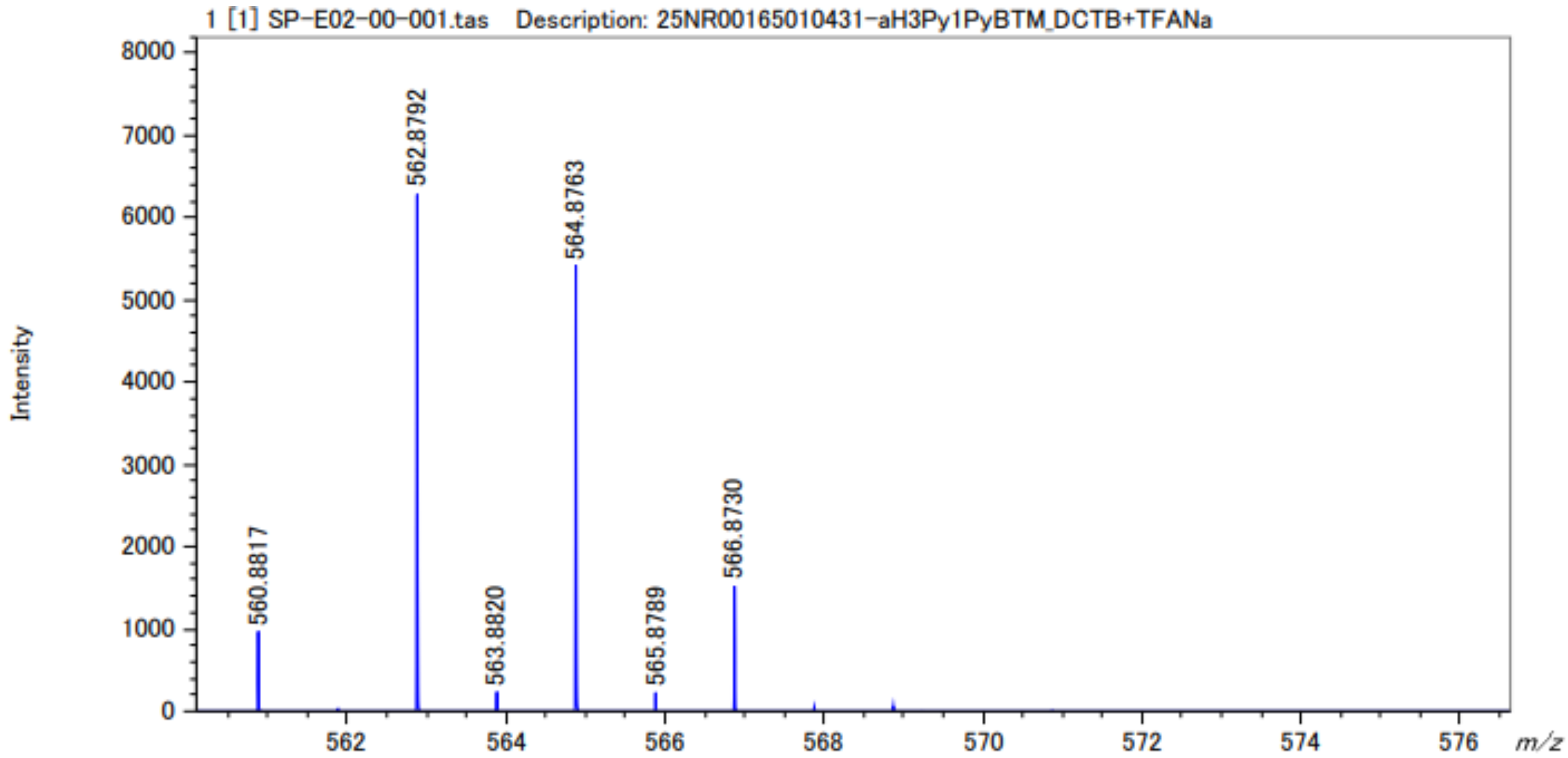

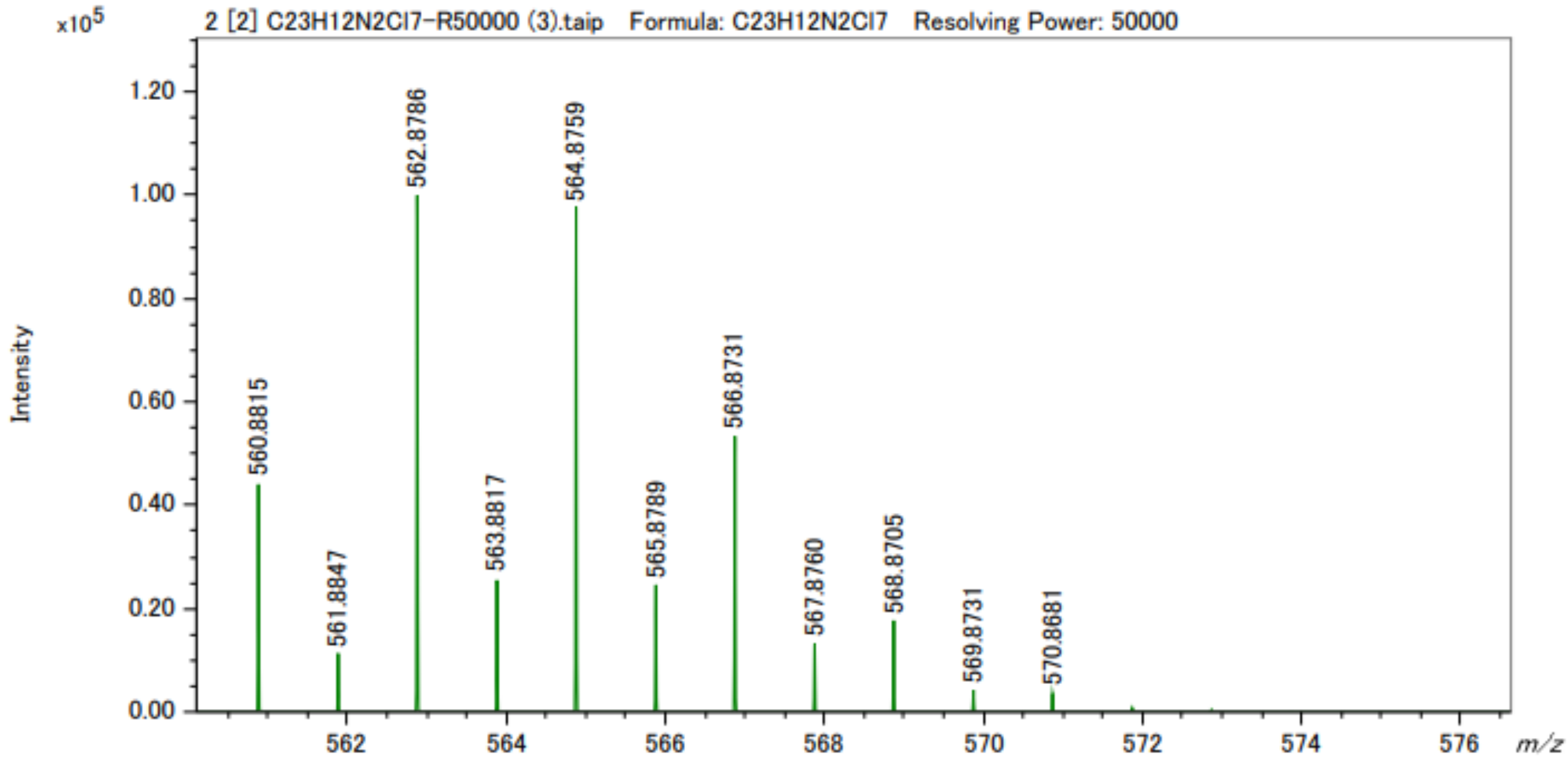

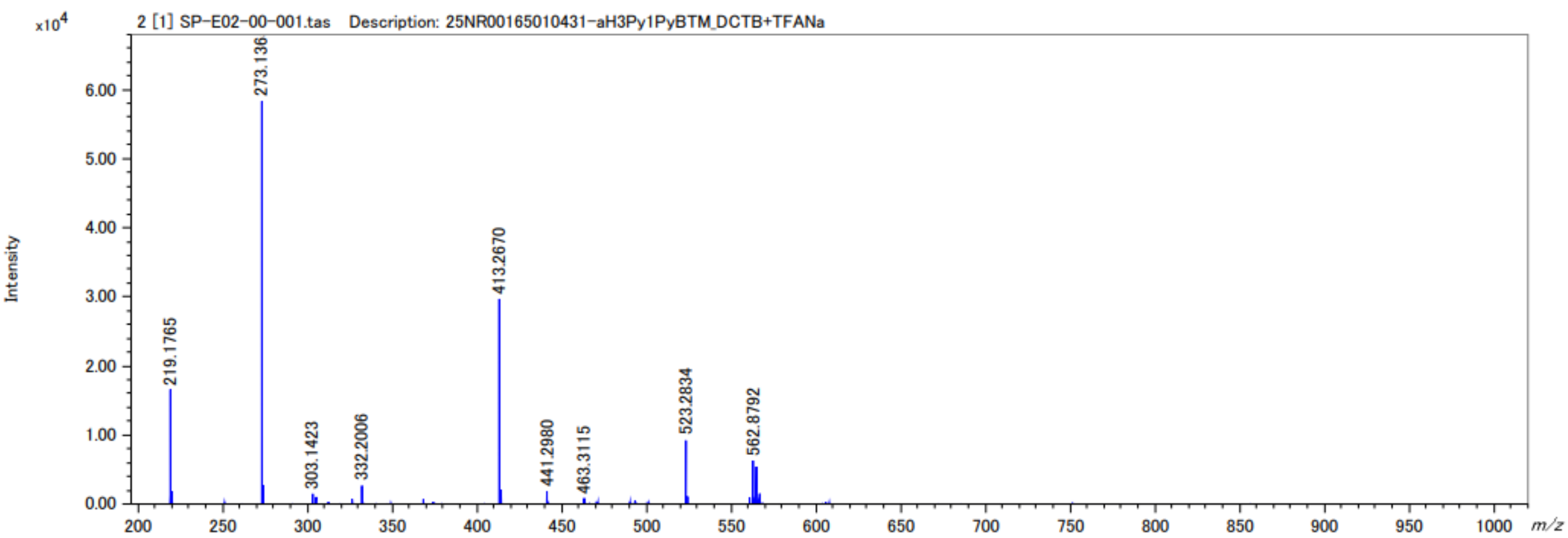

HR-MALDI-TOF mass spectrum of **aH-3Py$_2$PyBTM**, full spectrum (bottom) and calculated isotopic pattern (center) and experimental isotopic pattern (top) of the molecular peak.

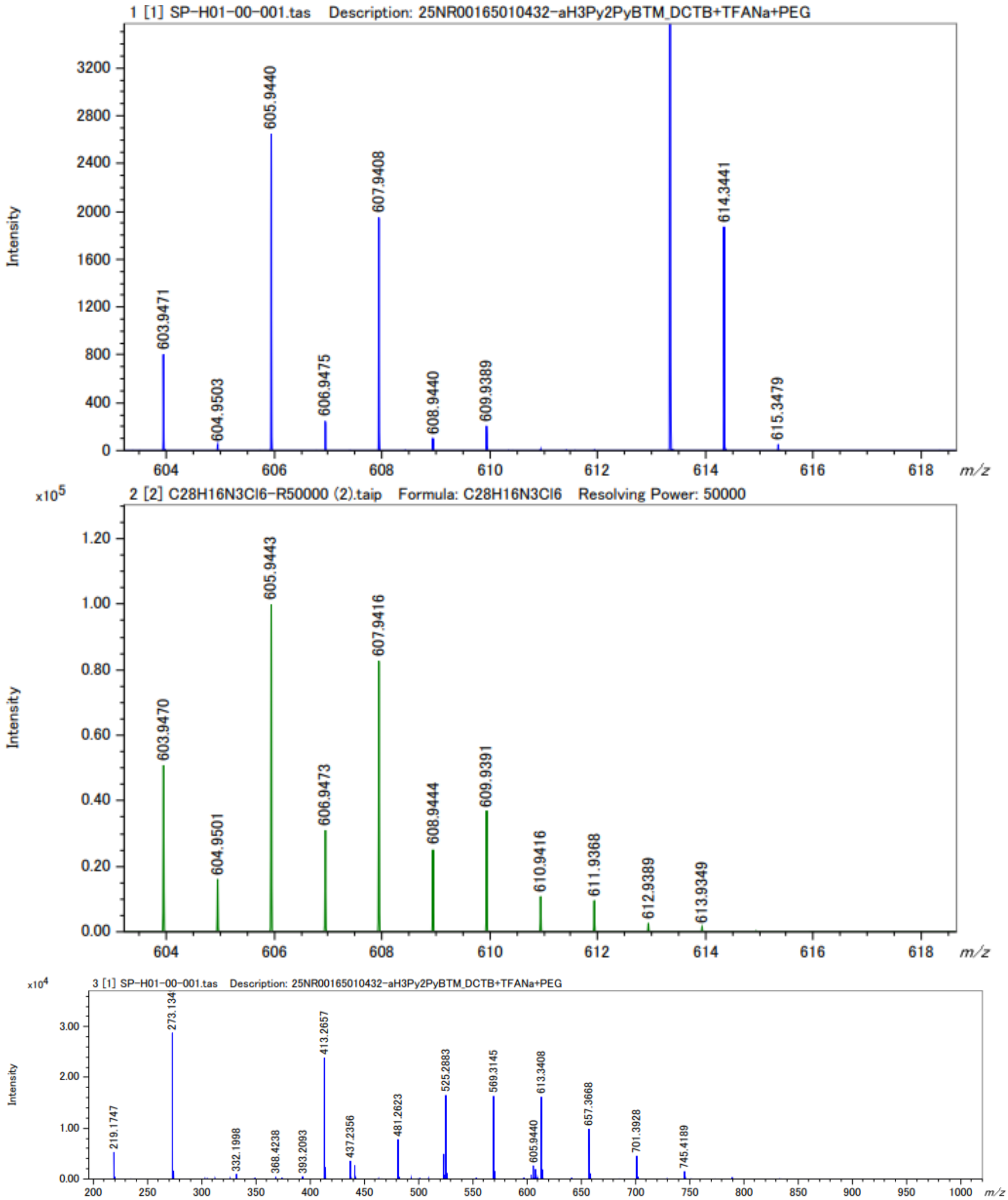

HR-MALDI-TOF mass spectrum of **aH-MeO3PyPyBTM**, full spectrum (bottom) and calculated isotopic pattern (center) and experimental isotopic pattern (top) of the molecular peak.

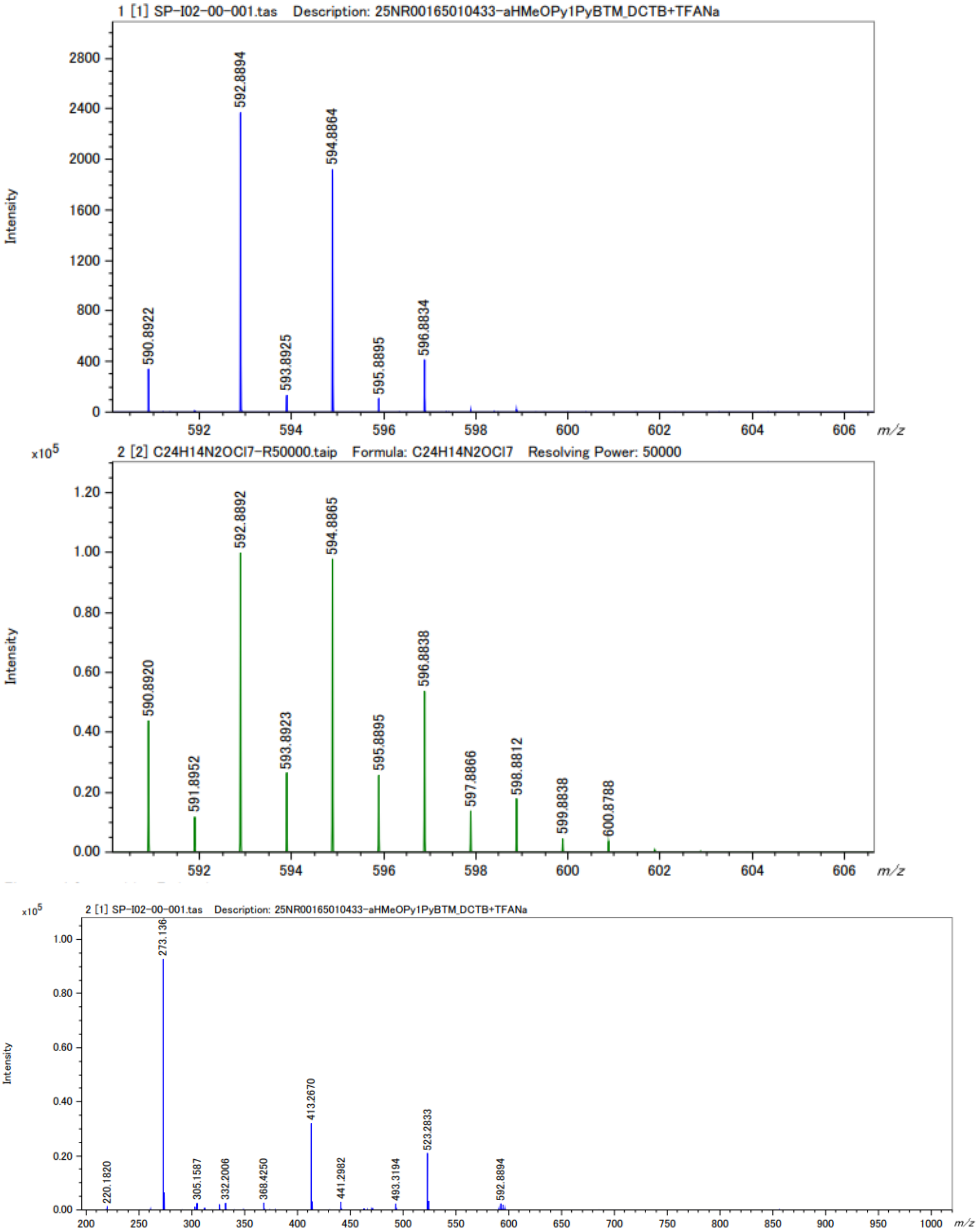

HR-MALDI-TOF mass spectrum of **aH-MeO3Py$_2$PyBTM**, full spectrum (bottom) and calculated isotopic pattern (center) and experimental isotopic pattern (top) of the molecular peak.

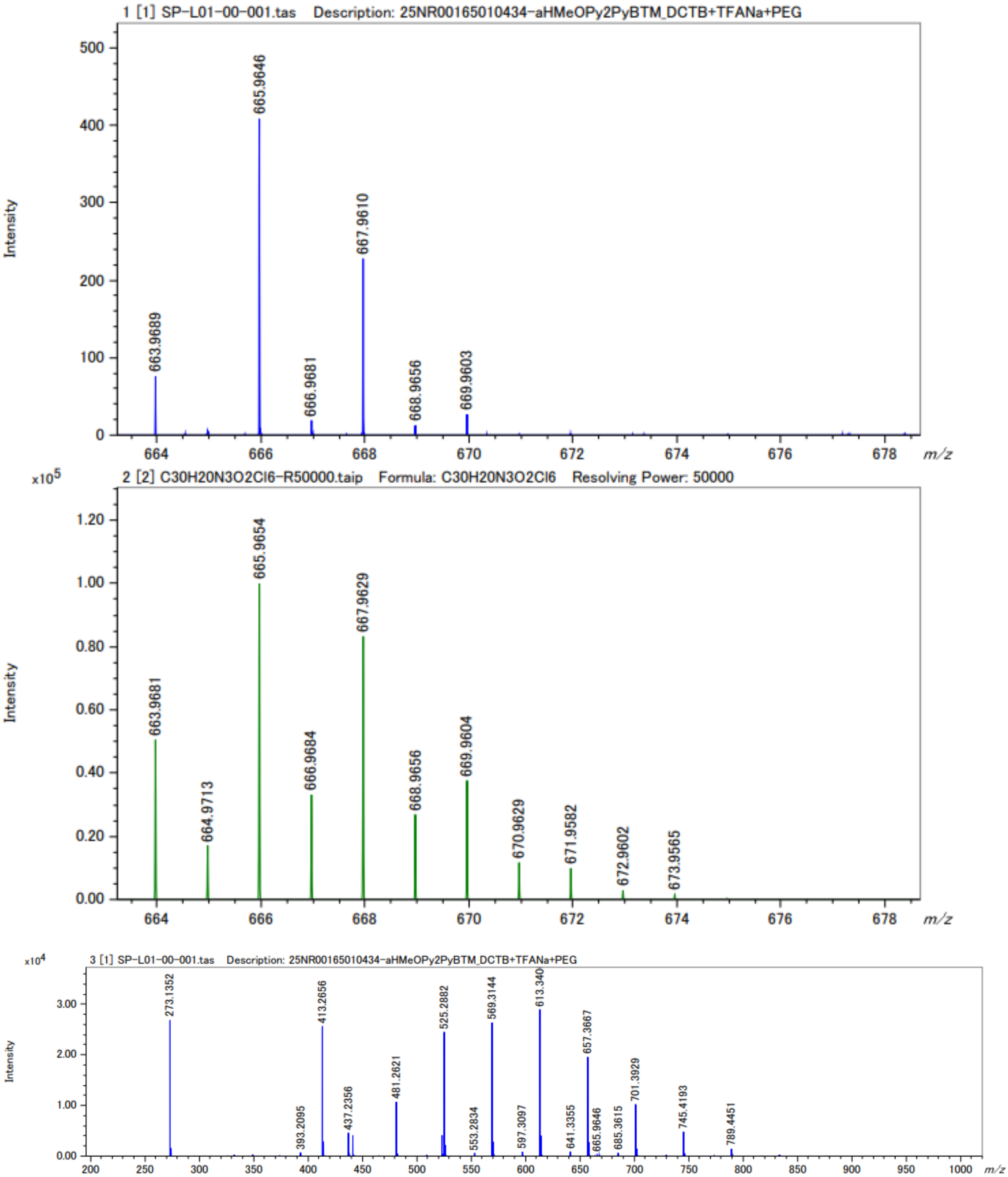

HR-MALDI-TOF mass spectrum of **4PyPyBTM**, full spectrum (bottom) and calculated isotopic pattern (center) and experimental isotopic pattern (top) of the molecular peak.

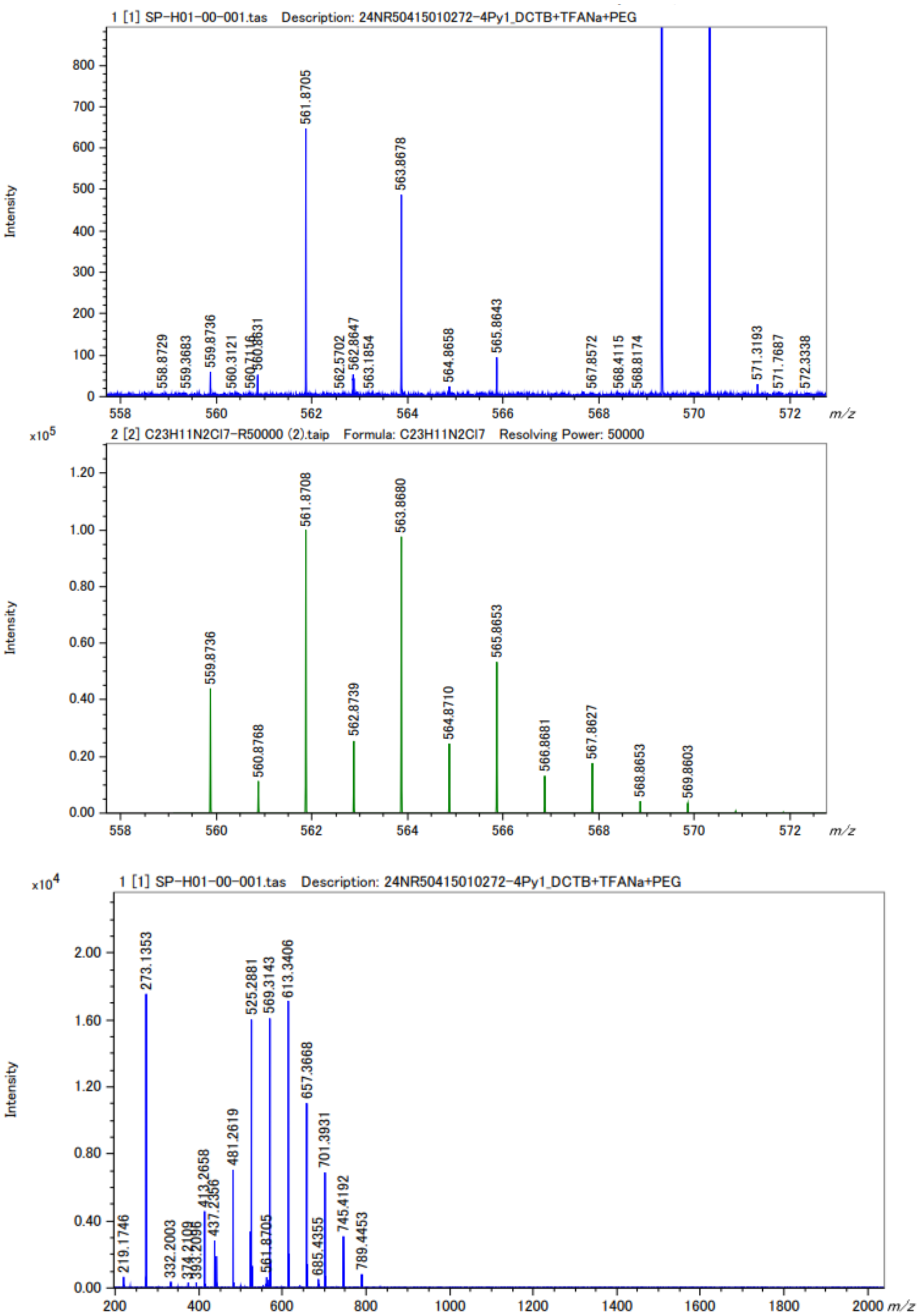

HR-MALDI-TOF mass spectrum of **4Py$_2$PyBTM**, full spectrum (bottom) and calculated isotopic pattern (center) and experimental isotopic pattern (top) of the molecular peak.

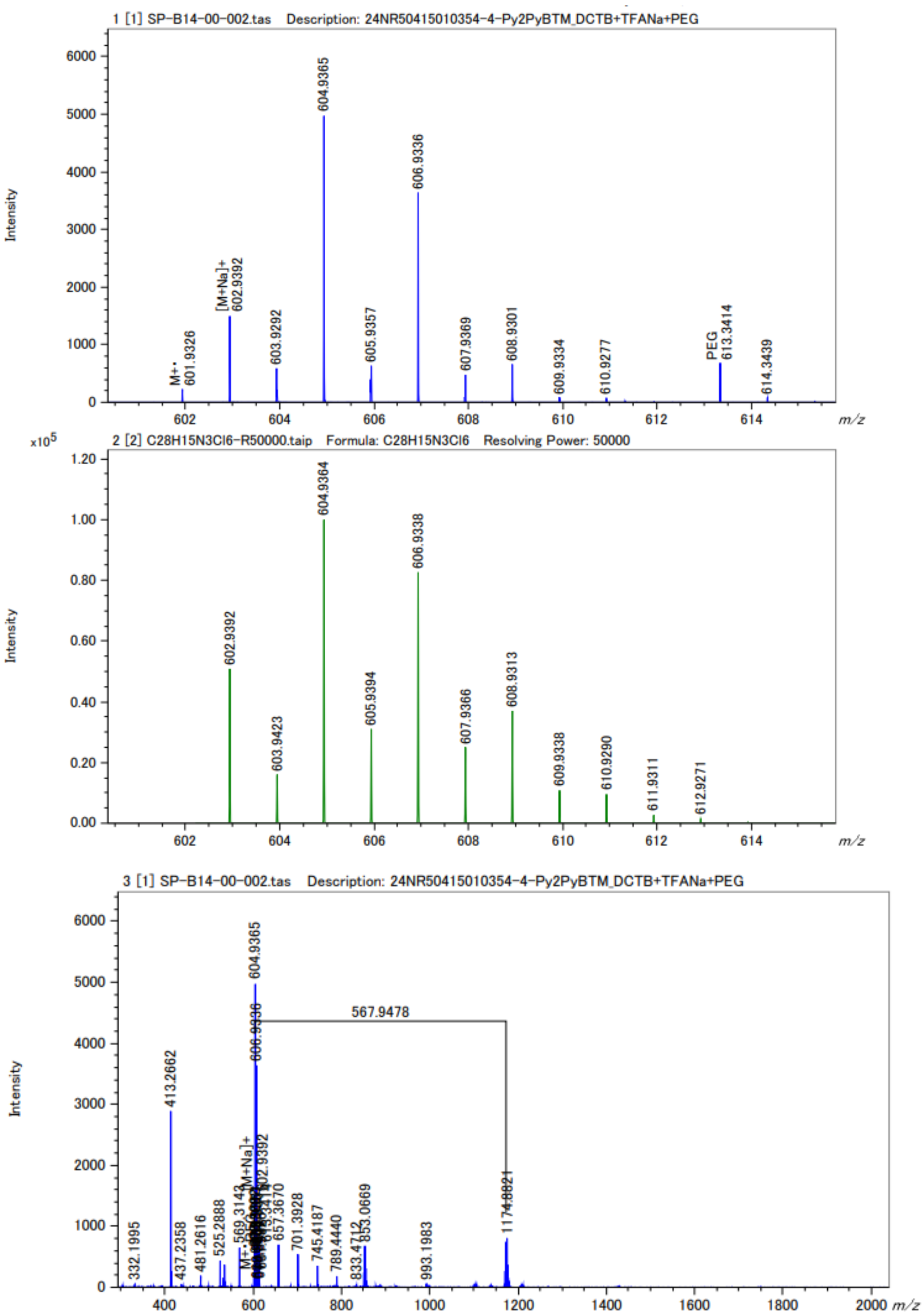

HR-MALDI-TOF mass spectrum of **3PyPyBTM**, full spectrum (bottom) and calculated isotopic pattern (center) and experimental isotopic pattern (top) of the molecular peak.

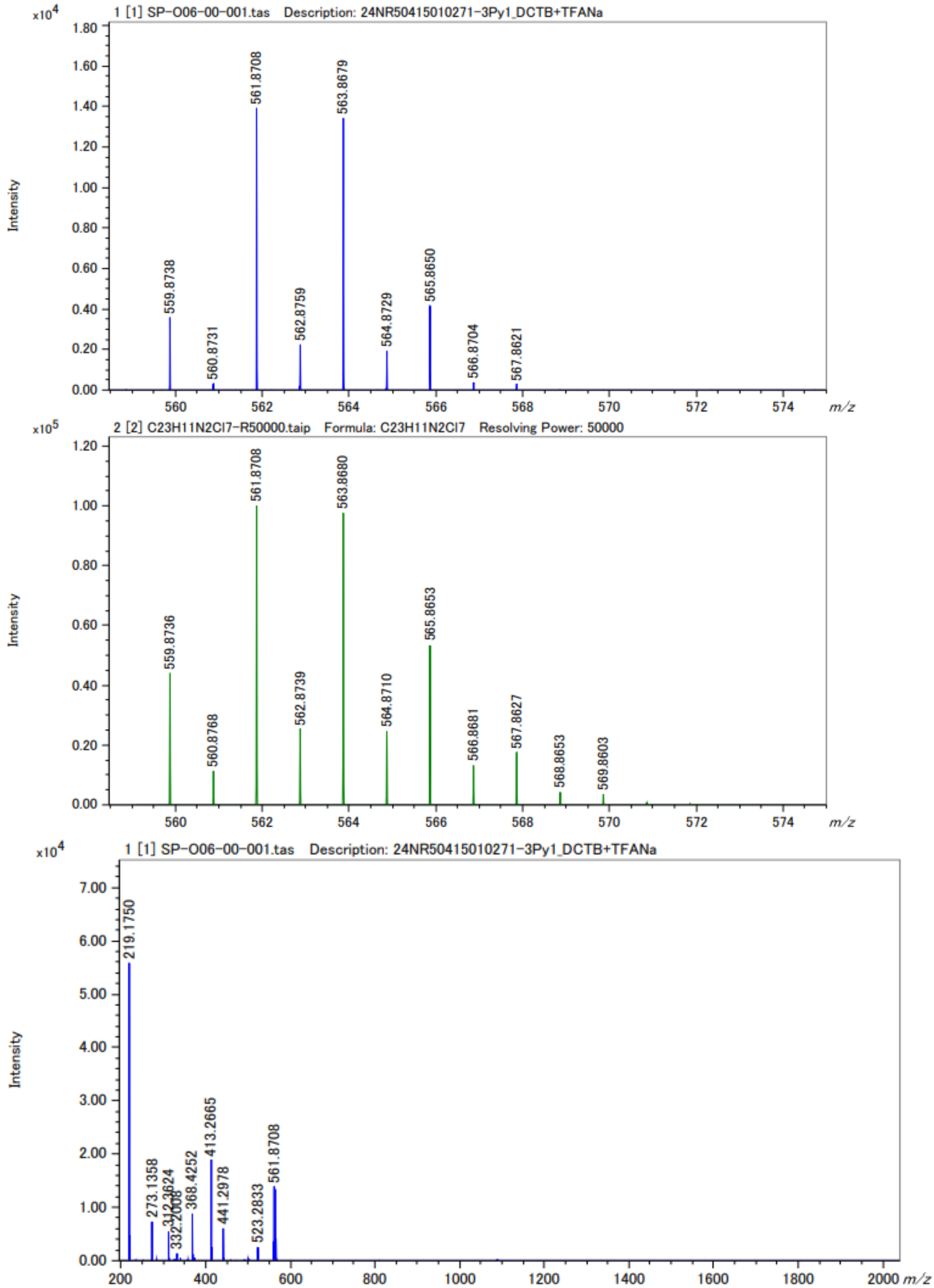

HR-MALDI-TOF mass spectrum of **3Py$_2$PyBTM**, full spectrum (bottom) and calculated isotopic pattern (center) and experimental isotopic pattern (top) of the molecular peak.

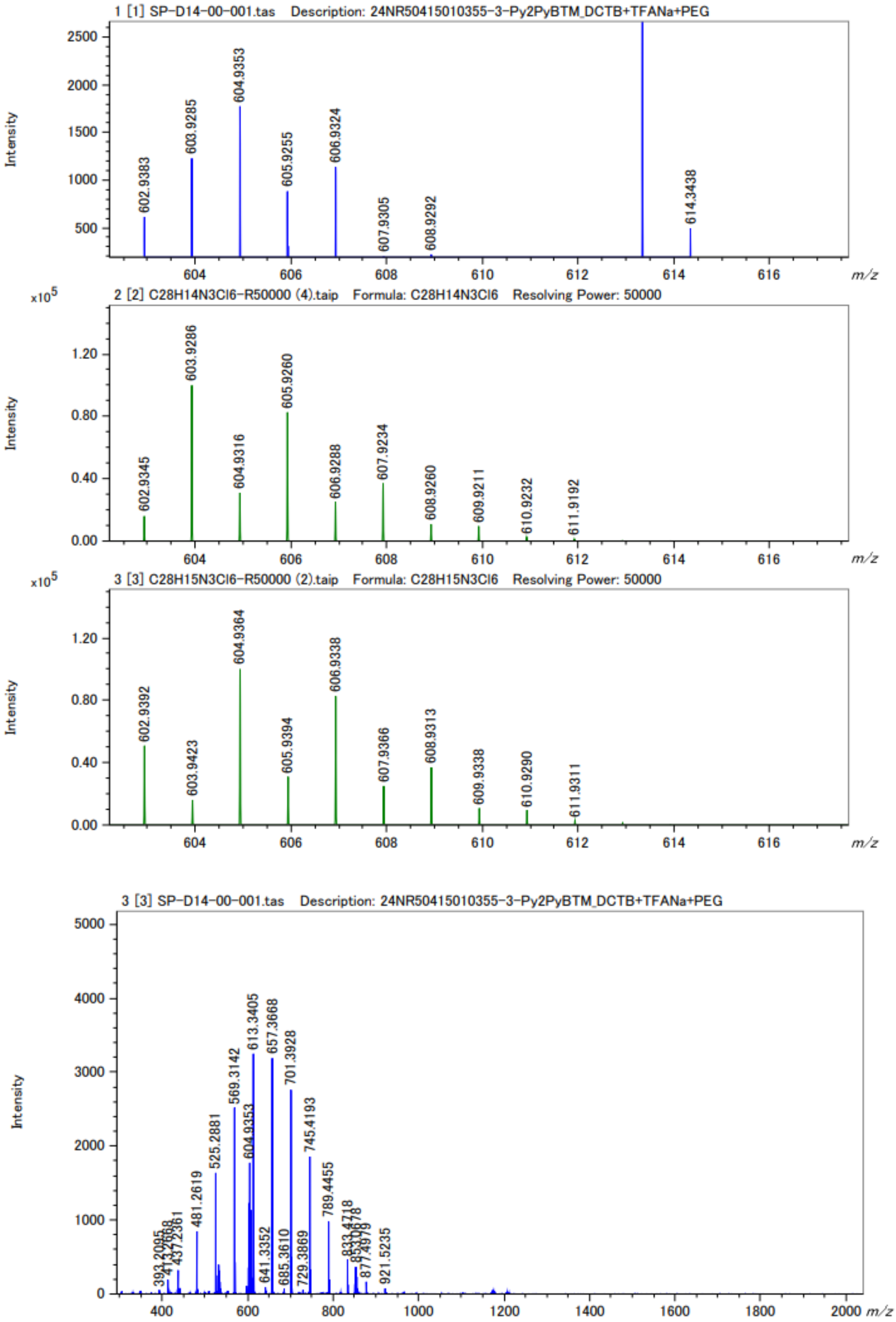

HR-MALDI-TOF mass spectrum of **MeO3PyPyBTM**, full spectrum (bottom) and calculated isotopic pattern (center) and experimental isotopic pattern (top) of the molecular peak.

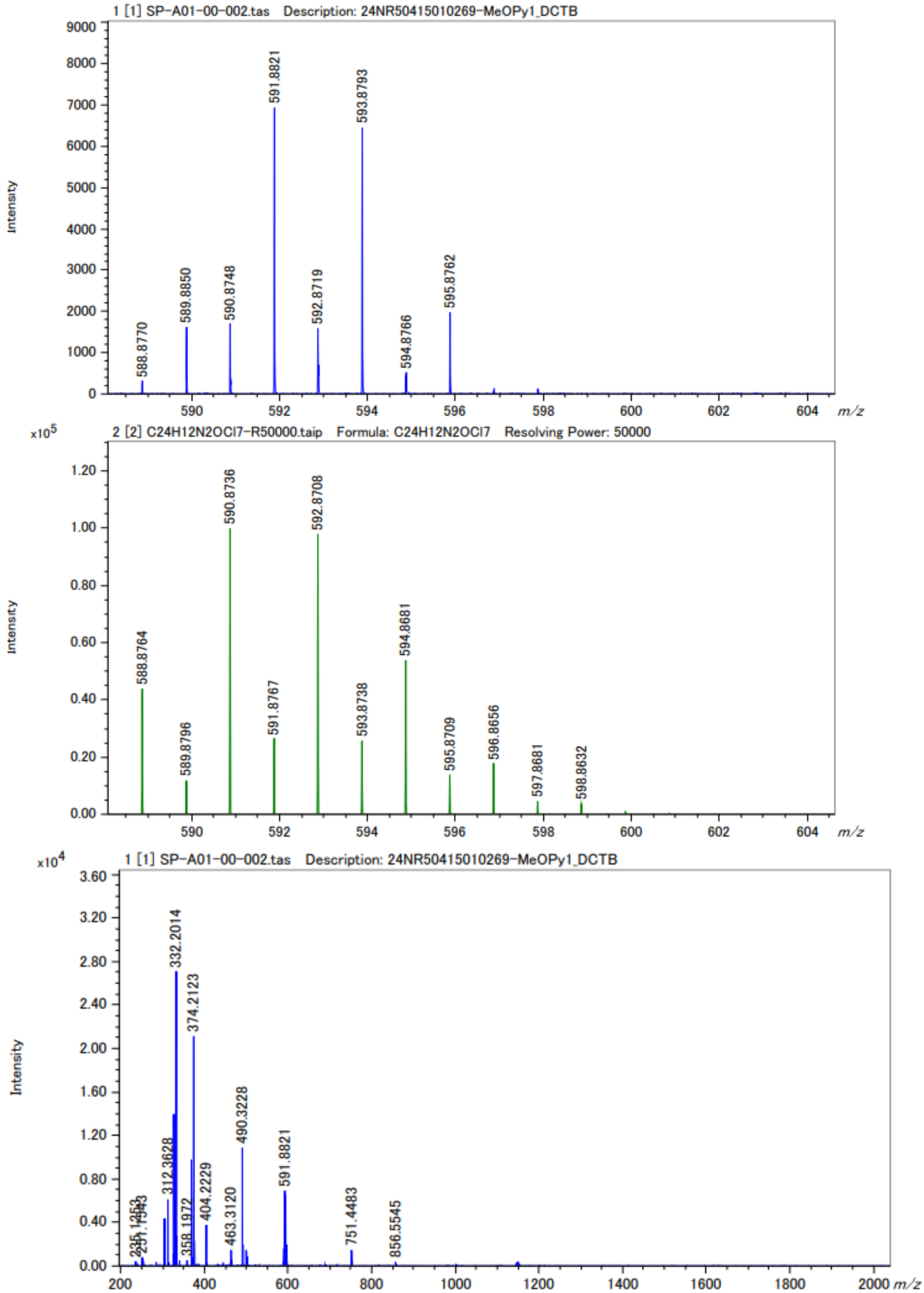

HR-MALDI-TOF mass spectrum of **MeO3Py$_2$PyBTM**, full spectrum (bottom) and calculated isotopic pattern (center) and experimental isotopic pattern (top) of the molecular peak.

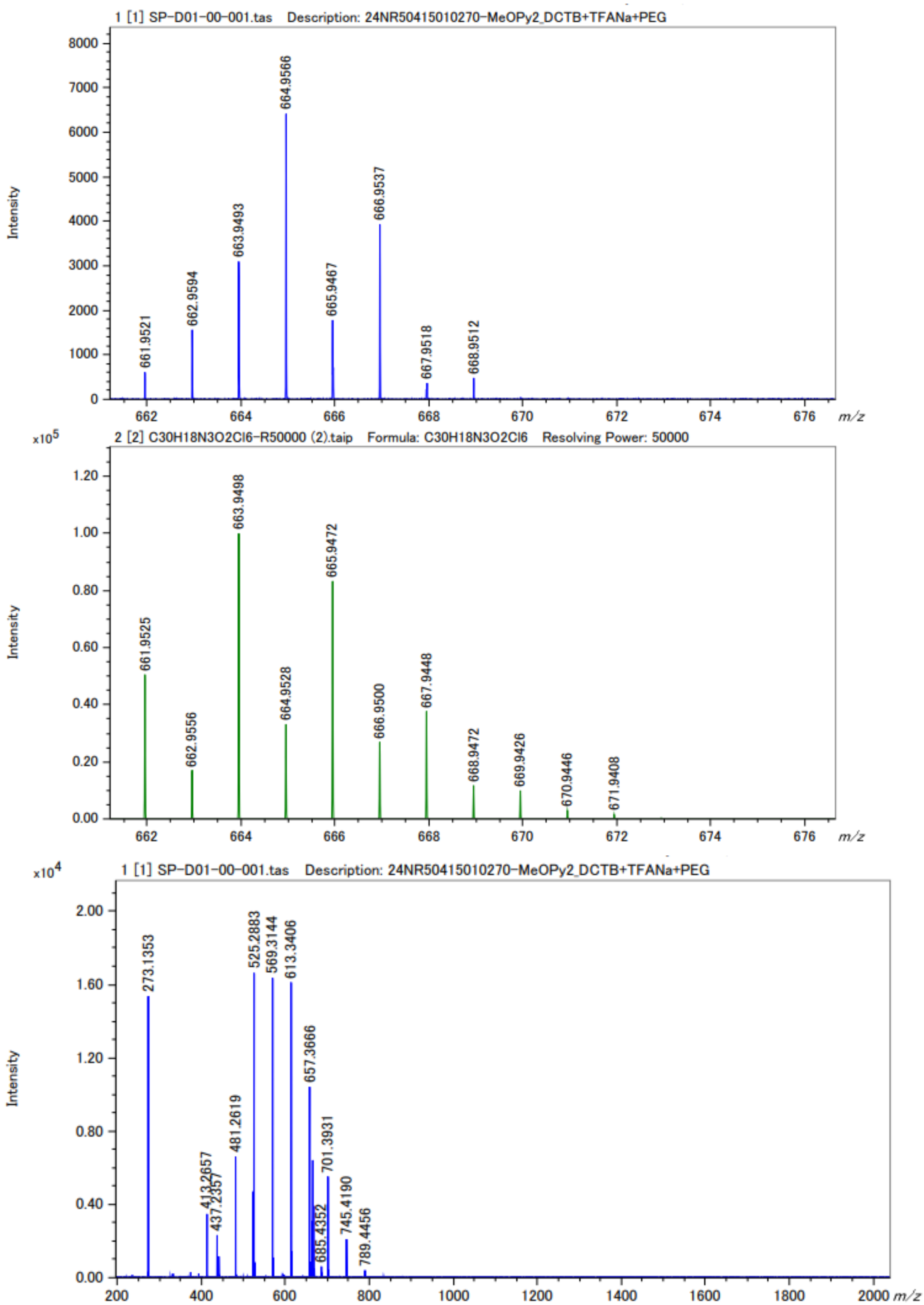

Supplement: Supplementary file 1 [file CHEM-32-e71095-s001.pdf]
